# Potential for host-symbiont communication via neurotransmitters and neuromodulators in an aneural animal, the marine sponge *Amphimedon queenslandica*

**DOI:** 10.3389/fncir.2023.1250694

**Published:** 2023-09-29

**Authors:** Xueyan Xiang, Arturo A. Vilar Gomez, Simone P. Blomberg, Huifang Yuan, Bernard M. Degnan, Sandie M. Degnan

**Affiliations:** Centre for Marine Science, School of the Environment, The University of Queensland, Brisbane, QLD, Australia

**Keywords:** *Amphimedon*, dopamine, neurotransmitter evolution, origin of nervous system, symbiosis, trace amines

## Abstract

Interkingdom signalling within a holobiont allows host and symbionts to communicate and to regulate each other’s physiological and developmental states. Here we show that a suite of signalling molecules that function as neurotransmitters and neuromodulators in most animals with nervous systems, specifically dopamine and trace amines, are produced exclusively by the bacterial symbionts of the demosponge *Amphimedon queenslandica*. Although sponges do not possess a nervous system, *A. queenslandica* expresses rhodopsin class G-protein-coupled receptors that are structurally similar to dopamine and trace amine receptors. When sponge larvae, which express these receptors, are exposed to agonists and antagonists of bilaterian dopamine and trace amine receptors, we observe marked changes in larval phototactic swimming behaviour, consistent with the sponge being competent to recognise and respond to symbiont-derived trace amine signals. These results indicate that monoamines synthesised by bacterial symbionts may be able to influence the physiology of the host sponge.

## Introduction

Some signalling molecules that are used as neurotransmitters and neuromodulators in neural animals appear to be involved in interkingdom signalling between host animals and their symbiotic bacteria. The most widely studied of these are between gut microbiota and their human hosts. Gut bacteria produce and release various signal metabolites that enable bidirectional host-symbiont communication through the microbiota-gut-brain axis, including nitric oxide, acetylcholine, serotonin, dopamine, noradrenaline, GABA, trace amines and short-chain fatty acids (Sobko et al., 2006; Collins et al., 2012; Galland, 2014; Mazzoli and Pessione, 2016; Mittal et al., 2017; de la Fuente-Nunez et al., 2018; Silva et al., 2020; Chang et al., 2021; Miri et al., 2023). These symbiont-derived neuroactive molecules can directly and indirectly affect human central and enteric nervous, endocrine and immune systems, to influence host physiology (Carabotti et al., 2014; Mazzoli and Pessione, 2016; Martin et al., 2018; Silva et al., 2020). Conversely, the host can also shape the composition of the gut microbiota *via* these same signalling molecules (Collins et al., 2012; Galland, 2014; Miri et al., 2023).

These symbiont signals appear to act largely through the host’s G-protein coupled receptors (GPCRs) (Cohen et al., 2017; Husted et al., 2017; Chen et al., 2019; Colosimo et al., 2019; Pandey et al., 2019). Gut microbiota metabolites, including phenylpropanoic acid, cadaverine, 9-10-methylenehexadecanoic acid, 12-methyltetradecanoic acid and trace amines, are ligands of specific human GPCRs and trigger distinct physiological responses in the host (Chen et al., 2019; Colosimo et al., 2019). For example, *Bacteroides thetaiotaomicron* produces the essential amino acid phenylalanine, which is an agonist for adhesion GPCRs, namely GPR56 and GPR97. Phenylalanine can be converted by another gut strain, *Morganella morganii*, into the trace amine phenethylamine, which readily crosses the blood–brain barrier to activate dopamine receptors (Chen et al., 2019). Through this signalling interaction, the symbiotic bacteria can significantly impact local and systemic host physiology (Carabotti et al., 2014; Mazzoli and Pessione, 2016; Martin et al., 2018; Silva et al., 2020).

These observed interactions between bacterial and human cells in the gut and elsewhere raises the possibility that neurotransmitter signalling was co-opted from a more ancestral animal-bacterial symbiont signalling system that predates the origin of the nerve cell (Klimovich and Bosch, 2018; O’Donnell et al., 2020; Bosch and McFall-Ngai, 2021). This hypothesis is supported by the following observations: (i) the bacterial pathways that produce metabolites, which are equivalent to animal neurotransmitters and neuromodulators, are conserved and ancient; (ii) the origin of rhodopsin class GPCRs (Rh-GPCRs) predates the origin of animals; and (iii) it is very likely that animals originated and evolved in the presence of bacteria and that the last common ancestor to all extant animals hosted symbiotic bacteria (McFall-Ngai et al., 2013; de Mendoza et al., 2014; Schretter et al., 2018; O’Donnell et al., 2020; Bosch and McFall-Ngai, 2021).

To further investigate the hypothesis that neurosignalling evolved from an ancestral animal-bacterial signalling system, here we seek evidence from sponges (phylum Porifera), one of the earliest-diverging phyletic lineages of extant animals (Simion et al., 2017; Schultz et al., 2023). Sponges are morphologically simple animals that lack a nervous system (Leys, 2015; Musser et al., 2021). They host symbiotic microbial communities, which vary in complexity and abundance depending on the species (Hentschel et al., 2012; Thomas et al., 2016; Steinert et al., 2020).

Despite lacking a nervous system, sponges can respond to a range of stimuli (Leys and Degnan, 2001; Elliott and Leys, 2010; Leys, 2015; Ueda et al., 2016; Francis et al., 2017; Mah and Leys, 2017; Leys et al., 2019; Say and Degnan, 2020; Musser et al., 2021; Wong et al., 2022; Kornder et al., 2022). Their genomes also encode a large repertoire of GPCRs similar in composition and structure to other metazoans, including many lineage-specific Rh-GPCR genes organised in clusters in the genome, and putative metabotropic glutamate (mGluRs), GABA, adrenergic, serotonin, trace amine and dopamine receptors (Srivastava et al., 2010; Riesgo et al., 2014; Krishnan et al., 2015; Francis et al., 2017; Goulty et al., 2023). Some ligands of these GPCRs can be synthesised by the sponges themselves, such as glutamate and GABA, but others in general can not; these latter include dopamine, serotonin and adrenaline (Elliott and Leys, 2010; Srivastava et al., 2010; Francis et al., 2017; Mah and Leys, 2017; Leys et al., 2019). Serotonin and serotonin-like molecules found in sponges are considered to be produced by their bacterial symbionts (Hedner et al., 2006; Leys, 2015), and the ability of nitric oxide to induce larval settlement in the demosponge *Amphimedon queenslandica* requires the production of its precursor, arginine, by its horizontally-inherited symbiotic bacteria (Song et al., 2020).

Here we provide evidence that interkingdom signalling between a marine sponge and its bacterial symbionts may be occurring via bacterial metabolites that are identical to neurotransmitters and neuromodulators. Focussing on dopamines and trace amines in the *A. queenslandica* holobiont (Srivastava et al., 2010; Fernandez-Valverde et al., 2015; Fieth et al., 2016; Gauthier et al., 2016; Xiang et al., 2022), we show these signalling molecules can only be produced by the sponge’s maternally-inherited bacterial symbionts, and that their putative receptors are developmentally expressed in the host’s swimming larval stage. Applying agonists and antagonists of bilaterian dopamine and trace amine receptors to swimming larvae, we show that these potentially symbiont-derived signalling molecules can influence the behaviour of its sponge host.

## Materials and methods

### Characterisation of dopamine and trace amine biosynthesis pathways

To identify gene models, we used previous annotations of the *A. queenslandica* Aqu2.1 genome and the genomes of its three primary symbionts *AqS1*, *AqS2* and *AqS3* (Fernandez-Valverde et al., 2015; Xiang et al., 2022). Biosynthetic and signalling pathways of *A. queenslandica*, *AqS1*, *AqS2* and *AqS3,* were reconstructed based on the KEGG annotations resulting from KEGG mapper (Kanehisa et al., 2016). Protein coding sequences that have no orthologues in the KEGG database or were missing from specific KEGG pathways were manually annotated using Blast2GO as previously described (Xiang et al., 2022).

### Identification and characterisation of putative dopamine and trace amine receptor genes

All Rh-GCPRs identified in version 1.0 of the *A. queenslandica* genome (Srivastava et al., 2010; Krishnan et al., 2015) were used to identify Rh-GPCRs in the *Aqu2.1* genome (Fernandez-Valverde et al., 2015) using BLASTP to sequences in GenBank and in Ensembl Metazoa (August 2021). Putative *A. queenslandica* Rh-GPCRs were assigned to a specific subfamily following the methods of Krishnan et al. (2015), with subfamily assignment occurring only if 45% of BLASTP hits in GenBank were to a specific subfamily.

Transmembrane (TM) domains were identified in potential dopamine (DRD) or trace amine (TAAR)-like receptor coding sequences using TMHMM Server v. 2.0 (Krogh et al., 2001). GPCR topologies were predicted and visualised using TOPO2 (Johns, 2021), and compared with bilaterian DRD or TAAR-like receptors (Civelli et al., 1992; Missale et al., 1998; Zhuang et al., 2021). Genomic sequences 1.5 kb up and downstream of gene models with less than seven TMs were translated using ExPASy DNA/RNA Translation tool (Gasteiger et al., 2003) and coding sequences assessed for TM domains using TMPred (Stoffel and Hofmann, 1993), MemBrain (Yin et al., 2017), CCTop (Dobson et al., 2015), MEMSAT (Jones et al., 1994), PredictProtein (Bernhofer et al., 2021), PSIPRED (Buchan and Jones, 2019), SPLIT4 (Juretić et al., 2002) and MEMPACK (Nugent et al., 2011). Hydrophobicity plots from ExPasy-ProtScale (Gasteiger et al., 2003) and TOPO2 (Johns, 2021) were used to corroborate predicted TM domains. Putative *A. queenslandica* DRD- or TAAR-like receptor sequences were aligned to human (*Homo sapiens*), rat (*Rattus norvegicus*) and fruit fly (*Drosophila melanogaster*) sequences using web-based Clustal Omega (Madeira et al., 2019), so that conserved amino acid residues could be identified.

### Analysis of *DRD-* and *TAAR-*like expression

The expression of *A. queenslandica DRD-* and *TAAR*-like genes were characterized using previously published developmental and cell type RNA-Seq data sets (NCBI Accession numbers PRJNA258388, PRJNA694780, PRJNA412708 and PRJNA435744) (Gaiti et al., 2015; Levin et al., 2016; Sebé-Pedrós et al., 2018; Sogabe et al., 2019; Wong et al., 2022). All raw expression counts generated using CEL-Seq2 were normalised using the Bioconductor package DESeq2 counts function and the ‘normalised = TRUE’ argument (Love et al., 2014). Raw expression counts generated using single cell MARS-Seq were normalised using the R package edgeR counts per million (cpm) function with the ‘log = TRUE’ and ‘lib.sizes = TRUE’ argument (Robinson et al., 2010). Replicate samples for the same developmental stages or cell types were averaged and Z-scores calculated to compare mean expression within transcriptomes. Box plots of all genes were generated using the ggplot2 package in R (Wickham, 2016).

### Larval phototaxis assays

Reproductive adults of *Amphimedon queenlandica* were collected from Heron Island Reef, southern Great Barrier Reef, Australia (23^o^26′ S, 151^o^55′ E) and maintained in a closed aquarium system as described in Leys et al. (2008). Larval release was induced by heating the aquarium sea water by 1–2°C and larvae were collected and maintained in 0.22 μm filtered artificial seawater (FSW) in daylight at 25°C. All larval phototactic swimming assays were performed as described in Wong et al. (2022). Briefly, ten larvae were added to the bright end of a transparent chamber (7.5 × 2.2 × 1.3 cm) that was filled with 20 mL FSW and had an ecologically relevant light gradient along the long axis of the chamber, with 950 and 80 mM photons m^−2^ s^−1^ at the bright and dark ends, respectively (Leys and Degnan, 2001; Wong et al., 2022). All FSW controls and treatments were repeated three times. The swimming behaviour of larvae in the chamber was filmed and the number of larvae appearing in each of four equally sized quartiles in the chamber (Q1–Q4 from bright end to dark end) was scored every 5 s for 30 s as previously described (Wong et al., 2022).

Six known agonists and antagonists of bilaterian dopamine and trace amine-like receptors were tested in the larval phototaxis assays (Table 1; Xu and Li, 2020). Dopamine receptor agonist (rotigotine hydrochloride) and antagonist (flupenthixol dihydrochloride) were obtained from Abcam (Melbourne, Australia) and all trace amine receptor agonists were obtained from Sigma & Aldrich (Sydney, Australia). To determine optimal assay concentrations of all agonists or antagonists, ten *A. queenslandica* larvae were subjected to 10^−3^, 10^−4^, 10^−5^, 10^−6^ or 10^−7^ M of each reagent, and larval behaviour and health was recorded over 60 min. In all cases, the highest concentration that had no effect on health, morphology or larval swimming was used for subsequent larval phototaxis assays. Each reagent was added to FSW in the chamber to a final assay concentration (Table 1) and larvae were pre-incubated in the same concentration of reagent in FSW for 3 min immediately prior to being transferred into the light chamber for the assay. Following phenethylamine and tryptamine assays, larvae were washed three times in FSW and subject to the same phototaxis assay in FSW. They were deemed normal if they displayed normal negative phototactic behaviour as in FSW controls. For comparison with previously published larval phototaxis assays in this species (Wong et al., 2022), stacked bar graphs to visualise the position of larvae and their distribution between chamber quadrats were generated.

**Table 1:** Reagents tested in larval phototaxis assay.

Reagent	Function	Solvent	Assay conc.
Rotigotine hydrochloride	DRD agonist	FSW	10 μM
Flupenthixol dihydrochloride	DRD antagonist	FSW	10 μM
Phenethylamine	Trace amine and TAAR agonist	FSW	100 μM
Tyramine	Trace amine and TAAR agonist	FSW	100 μM
Tryptamine	Trace amine and TAAR agonist	FSW	100 μM
D,L-metanephrine hydrochloride	General TAAR agonist	FSW	100 μM

### Statistical analysis

The effects of agonists and antagonists on larval phototactic swimming behaviour were analysed using Bayesian Generalised Additive Models (BGAMs), because the time course of movement through the experimental chamber was unlikely to be linear, and the smooth splines used by BGAMs can flexibly model nonlinear relationships. The response variable was the number of larvae in each quartile at each observation time, leading to a cumulative logit model. We modelled the relationship between the number of larvae in each quartile over time, for each agonist and antagonist (hereafter, drug) treatment, plus a control in which no drug was added. We fitted 4 models, which consisted of a common time course for all drug treatments versus a treatment-specific time course, and equidistant thresholds versus flexible thresholds. The hypothesis of equidistant thresholds determines whether the quartile widths, as perceived by the larvae, are all equal. As this was how the experimental chamber was constructed, we expected this to be the case. Each experimental run was treated as a random effect. All models were fitted using the brms package for R (Bürkner, 2017, 2018).

All models were run with the same default improper flat priors, 4 chains each with 4,000 iterations (1,000 warm up iterations) for a total of 12,000 post-warm up draws. The leave-one-out (LOO) information criterion was computed for all models, and posterior model weights based on the LOOIC were examined to determine the relative support for each model. For the two best models, we performed posterior-predictive plots to determine whether the models could produce data that “looked like” the observed data. Model diagnostics and plotting was conducted using brms (Bürkner, 2017, 2018).

## Results

### Only *Amphimedon* bacterial symbionts can synthesise dopamine and trace amines

**Figure 1:**
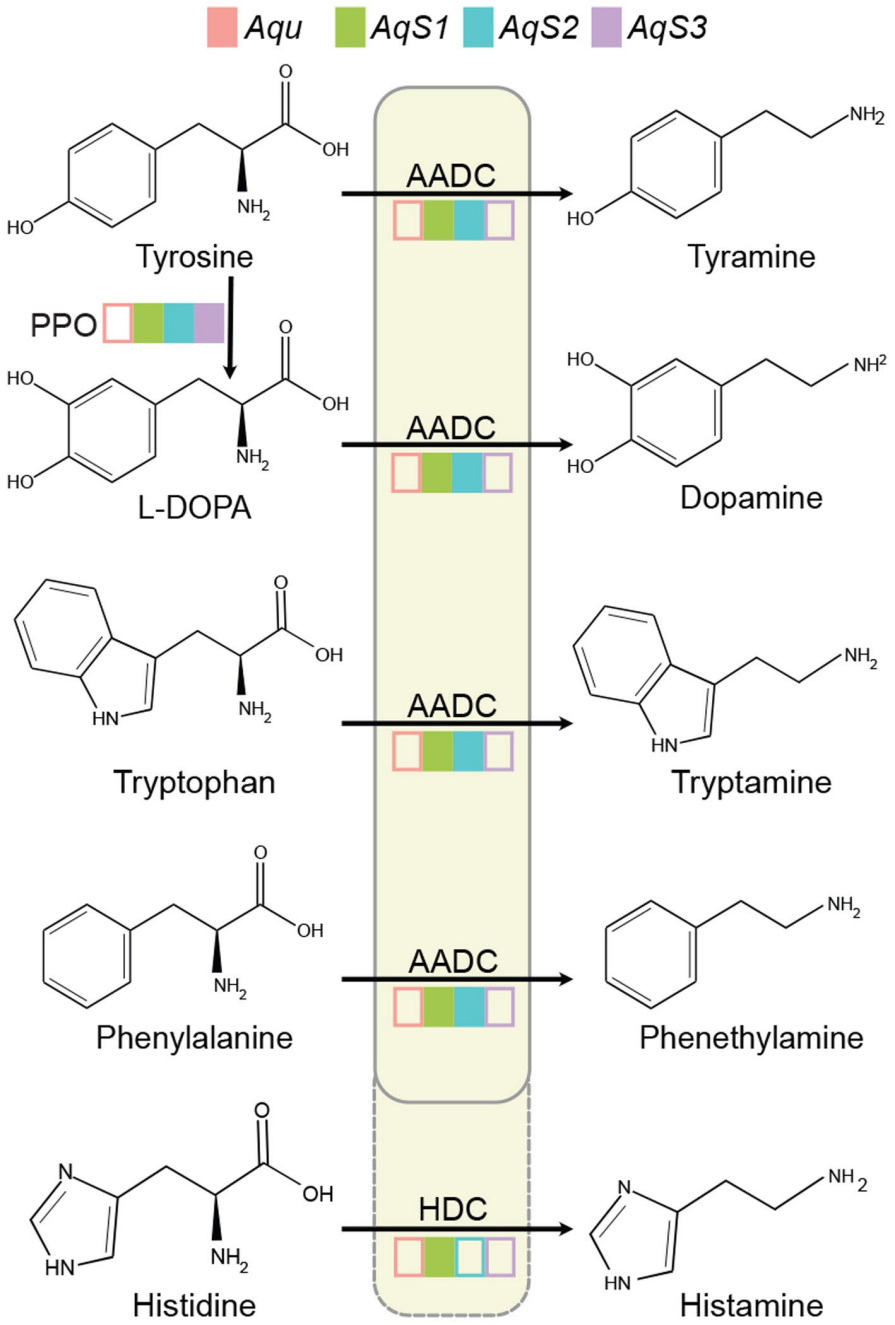
Tyramine, dopamine, tryptamine, phenethylamine and histamine synthesis in the *Amphimedon queenslandica* holobiont. Filled coloured boxes indicate the presence of the gene in the genome of sponge and bacterial symbiont species. Transcripts of all genes have also been detected in the adult sponge (Xiang et al., 2022). Empty coloured boxes indicate that the gene was not detected in the genome. Only tyrosine can be synthesised by both the sponge host and symbionts. *Aqu*, *A. queenslandica* genome; *AqS1*, *AqS2* and *AqS3*, three primary symbiont genomes (Xiang et al., 2022). AADC, aromatic L-amino acid decarboxylase (EC 4.1.1.28); HDC, histidine decarboxylase (EC 4.1.1.22); L-DOPA, L-3,4-dihydroxyphenylalanine; PPO, polyphenol oxidase (EC 1.10.3.1).

The three dominant vertically-inherited symbionts, *AqS1*, *AqS2* and *AqS3*, can comprise over 95% of total bacterial abundance in *A. queenslandica* larvae (Fieth et al., 2016; Gauthier et al., 2016; Xiang et al., 2022). Analysis of these genomes and the *A. queenslandica* genome indicate that only symbionts *AqS1* and *AqS2* have the capacity to synthesise dopamine, tyramine, tryptamine, phenethylamine and histamine (Figure 1). Dopamine can be converted from tyrosine through oxidation and decarboxylation by polyphenol oxidase (PPO, EC 1.10.3.1) and aromatic L-amino acid decarboxylase (AADC, EC 4.1.1.28), respectively. Tyramine, tryptamine, phenethylamine and histamine are derived from tyrosine, tryptophan, phenylalanine and histidine, which can also be catalysed by AADC (Komori et al., 2012). Histidine decarboxylase (HDC, EC 4.1.1.22) more likely converts histidine into histamine (Figure 1).

*AADC and HDC are members of the* larger PLP-dependent aspartate aminotransferase superfamily, and possess conserved catalytic residues Lys303 (amino acid site is based on the human protein) and His192 (Eliot and Kirsch, 2004; Liang et al., 2017). Diagnostic Ser354 in one *AqS1* and one *AqS2* gene, and Gly354 in one *AqS1* gene, support the presence of an *aadc* in *AqS1* and *AqS2*, and a *hdc* in *AqS1* (Supplementary Figure 1). Holobiont transcriptome data reveal that *AqS1* and *AqS2* have the potential to synthesise these signalling molecules, with both *aadc* and *hdc* mRNAs being above the mean transcript abundance levels in *AqS1* and *AqS2* (Xiang et al., 2022).

### Developmental and cell type expression of dopamine- and trace amine-like receptors

**Figure 2:**
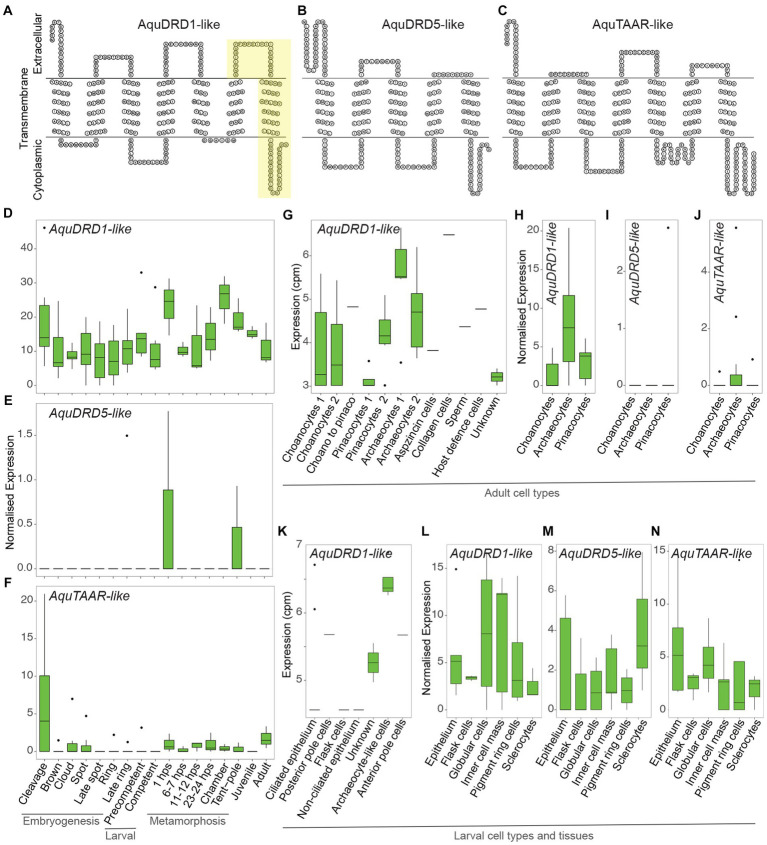
Structure and expression of AquDRD1-, AquDRD5- and AquTAAR-like. **(A–C)** Snake plots of AquDRD1-like **(A)**, AquDRD5-like **(B)** and AquTAAR-like **(C)** showing amino acid sequence (single letter code) and putative intracellular, transmembrane and extracellular regions; N-termini to the left. Yellow region in AquDRD1-like highlights the newly annotated 3rd extracellular loop and 7th TM domain missing from the Aqu2.1.30477 coding sequence (see Supplementary Figure 2). **(D–N)** Normalised gene expression levels of *AquDRD1-* **(D,G,H,K,L)**, *AquDRD5-* **(E,I,M)** and *AquTAAR-like* **(F,J,N)** (Supplementary Table 2). **(D-F)** Developmental expression through *A. queenslandica* embryogenesis (cleavage – late ring), larval development (precompetent and competent) and metamorphosis (1 hps postlarva – tent-pole postlarva), and in the juvenile and adult using CEL-Seq2 transcriptomes (normalised expression; Gaiti et al., 2015; Levin et al., 2016). **(G–K)** Cell-type specific gene expression levels using MARS-Seq **(G,K)** (counts per million, cpm; Sebé-Pedrós et al., 2018) and CEL-Seq2 transcriptomes **(H-J)** (normalised expression; Sogabe et al., 2019). **(L,M)** Gene expression levels in pooled larval cells and tissues using CEL-Seq2 transcriptomes (normalised expression; Wong et al., 2022).

We uncovered 130 putative Rh-GPCR genes in Aqu2.1 genome (Fernandez-Valverde et al., 2015), compared to the 126 genes identified in the Aqu1.0 genome (Srivastava et al., 2010; Krishnan et al., 2015); some larger gene models in Aqu1.0 were split into two genes in Aqu2.1 (Supplementary File 1). Employing a BLASTP screen as used by Krishnan et al. (2015), we identified two genes encoding putative dopamine-like receptors, namely Aqu2.1.30477 (*AquDRD1-like*) and Aqu2.1.23882 (*AquDRD5-like*) (Figures 2A,B; Supplementary Table 1) that are most similar to vertebrate D1-like D_5_ dopamine receptor and drosophilid D1-like D_1_ dopamine receptor, respectively. A single putative trace amine-associated receptor-like, Aqu2.1.16444 (AquTAAR-like) (Figure 2C; Supplementary Table 1), has high sequence identity to several subtypes of vertebrate TAAR receptors. Krishnan et al. (2015) identified these three GPCRs as potential dopamine receptors based on sequence similiarities. Inportantly, however, phylogenetic analysis in that study places them with very strong support into *A. queenslandica*-specific Rh-GPCR clades, namely Aq-Rho-A (for AquDRD5 and AquTAAR-like) and Aq-Rho-C (for AquDRD1) (see Figure 2 in Krishnan et al., 2015). This indicates they are not bilaterian orthologues.

*AquDRD1-like* (Aqu2.1.30477) encodes seven TM domains, with the 7^th^ domain being predicted based on hydrophobicity and topology of the C-terminal region from Ile-249 to Tyr-273 (Supplementary Figure 2). AquDRD1-like has high sequence identity to mammalian DRD1s and the *Drosophila* Dop1R1 dopamine receptor from TM2 to TM4, and possesses other DRD1 diagnostic features, including an Asp in TM3, two Ser in TM5 and one at the C-terminus, a Phe in TM6, a N-glycosylation site at the N-terminus, a short intracellular loop 3 and an extracellular loop 2 that is of similar length to other DRD1s (Supplementary Figure 2). Mammalian DRD1 features not present in AquDRD1-like include Cys in extracellular loops 2 and 3 and the C-terminus, and Asp in TM2. The C-terminus lacks Ser and Thr residues, and a N-glycosylation site in extracellular loop 2, although it does have two Asp residues that are important for binding dopamine (Civelli et al., 1992; Missale et al., 1998; Vallone et al., 2000; Zhuang et al., 2021). *AquDRD5-like* (Aqu2.1.23882) encodes five TM domains, and assessment of 1.5 kb flanking sequence in each direction did not uncover additional TMs. AquDRD5-like nonetheless possesses hallmarks of dopamine receptors, including Asp in TM3, two Ser in TM5, and a N-glycosylation site at the N-terminus. It is unclear whether the AquDRD5-like coding sequence is incomplete and if this gene model encodes a functional receptor. *AquTAAR-like* (Aqu2.1.16444) encodes seven TM domains, and has a sequence and structure most similar to different members of the TAAR-like receptor family.

Analysis of *AquDRD1-*, *AquDRD5-* and *AquTAAR-like* expression through development and in the adult reveals that *AquDRD1-like* is the most highly and widely expressed of the three genes (Figures 2D–N; Supplementary Table 2). *AquDRD1-like* is dynamically expressed throughout embryogenesis and metamorphosis, and in larvae, juveniles and adults, while the other two genes were detected only in a subset of developmental stages (Figures 2D–F). *AquDRD1* transcripts were the only ones detected using MARS-Seq, a single cell RNA-Seq approach with limited sequence depth, on larval and adult cells (Sebé-Pedrós et al., 2018; Figures 2G,K). Analysis of CEL-Seq2 transcriptomes made from pools of curated cells and tissues (Sogabe et al., 2019; Wong et al., 2022), which had deeper sequencing depth, detected *AquDRD5-* and *AquTAAR-like* in larval tissues and confirmed that *AquDRD1-like* is the most highly expressed.

*AquDRD1-like* is expressed at similar levels throughout most of development, with transcript abundance transiently increasing at the start of metamorphosis in 1 h post-settlement (hps) postlarvae and when choanocyte chambers begin to form later in metamorphosis (Figure 2D). In adults, *AquDRD1-like* is expressed in most cell types but is highest in archaeocytes; *AquTAAR-like* is lowly expressed in archaeocytes (Figures 2G,H,J). In larvae, *AquDRD1* was detected in a variety of cell and tissue types, while *AquDRD5-* and *AquTAAR-like* expression was detected in larval tissues but usually at a lower level (Figures 2K–N).

### Dopamine and trace amine agonists and antagonists affect larval phototaxis behaviour

**Figure 3:**
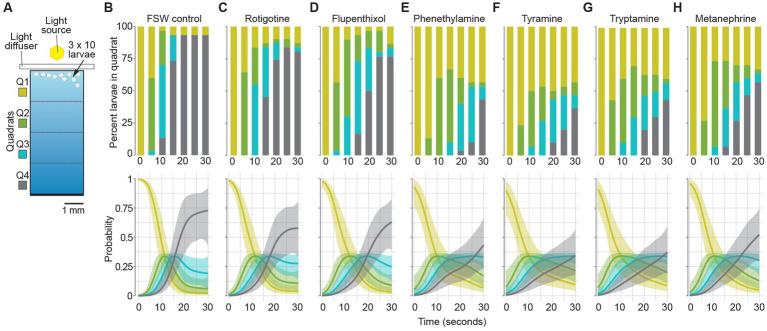
Effect of dopamine and trace amine agonists and antagonists on larval phototaxis. **(A)** Schematic of larval phototaxis assay chamber. Triplicate 30 s assays with 10 larvae loaded into Q1 (bright) quadrat were performed [see Section “Materials and methods,” and Wong et al. (2022) for details]. Prior to being placed in the assay chamber with light gradient, all larvae were incubated for 3 min in FSW with the agonist or antagonist at the concentration used in the assay (Table 1; see Section “Materials and methods”). **(B–H)** Top graphs, the percent larvae present in each quadrat (Q1–Q4) for each treatment every 5 s for 30 s. Bottom graphs, probability of larvae being found in quadrats for each treatment. Shaded areas are 95% credibility regions. **(B)** FSW positive control. **(C)** DRD agonist 10 μM rotigotine hydrochloride. **(D)** DRD antagonist 10 μM flupenthixol dihydrochloride. Trace amines and TAAR agonists: **(E)** 100 μM phenethylamine; **(F)** tyramine; and **(G)** tryptamine. **(H)** Non-specific TAAR agonist 100 μM *D,L-*metanephrine hydrochloride.

To determine if dopamine and trace amines can be detected by *A. queenslandica*, we targeted the larval stage because it is responsive to light and chemical stimuli (Leys and Degnan, 2001; Say and Degnan, 2020; Wong et al., 2022), and the three putative receptors are expressed at this stage (Figures 2K–M). *AquDRD1-like* is the most highly expressed gene in larval posterior pigment ring (pole) cells, which play a major role in larval swimming direction (Leys and Degnan, 2001; Rivera et al., 2012). Specifically, we assessed the impact of known bilaterian dopamine and trace amines agonist and antagonists on the natural negative phototaxis of the *A. queenslandica* larvae using an assay where we measure their swimming behaviour when subjected to a light gradient (see Section “Materials and methods”) (Figure 3A; Supplementary Figure 3).

We first determined the stage of larval development that has the highest sensitivity to light by subjecting three different aged cohorts (1–2, 4–6 and 10–12 h post emergence from the parental sponge; hpe) to a light gradient (Supplementary Figure 3). This analysis revealed that 4–6 hpe larvae exhibit a significantly higher level of negative phototaxis compared to both 1–2 and 10–12 hpe cohorts, with 93% of the larvae swimming to the dark end of the assay chamber (quartile 4; Q4) within 20–25 s of being placed at the bright end (Q1) (Supplementary Figure 3). Thus, all subsequent assays were performed on 4–6 hpe larvae. To confirm that larval swimming behaviour is in response to the light gradient, we dispensed 4–6 hpe larvae into (i) Q1 of the assay chamber (normally bright end) without a light gradient; and (ii) Q4 (dark end) with a light gradient (Supplementary Figure 3). These demonstrated that larvae actively swam away from the light and were not influenced by any current created by dispensing the larvae into the chamber (Supplementary Table 3).

We then compared the normal phototactic and swimming behaviours of 4–6 hpe larvae with similar aged larvae exposed to DRD and TAAR agonists and antagonists (Table 2; Figure 3; Supplementary Figure 4; Supplementary Table 3). Of the four models fitted, only 2 models had notable support. Both models allowed for different time courses for the different drugs. The only difference was the degree of flexibility in the threshold cutoffs, with the model forcing equidistant thresholds having a LOO weight of 0.527, compared to the LOO weight of 0.391 for the flexible threshold model. This indicated that the geometry of the experimental chamber corresponded to the phototactic behaviour of the larvae, as expected. Taken together, these two models provide 91.8% support for a treatment effect on the time course of the experiments, compared to models without a treatment effect. The time courses predicted from both models were almost identical, so we only present the results for the equidistant threshold model here (Figure 3; Supplementary Figure 4).

**Table 2:** Pairwise comparisons for the proportion of larvae that were in quadrat 4 at 25 s, assessed using Type II Wald chi square tests.

Comparison	Estimate	SE	*z* ratio	*p* value
FSW control – Rotigotine	−0.809	1.103	−0.733	0.9906
FSW control – Flupenthixol	−1.532	1.054	−1.454	0.7722
**FSW control** –**Phenethylamine**	**−5.210**	**1.173**	**−4.440**	**0.0002**
**FSW control** –**Tyramine**	**−4.333**	**1.082**	**−4.003**	**0.0012**
**FSW control** –**Tryptamine**	**−3.777**	**1.058**	**−3.569**	**0.0066**
FSW control – Metanephrine	−2.988	1.040	−2.874	0.0616
Rotigotine – Flupenthixol	−0.723	0.921	0.785	0.9864
**Rotigotine** –**Phenethylamine**	**−4.401**	**1.047**	**−4.206**	**0.0005**
**Rotigotine** –**Tyramine**	**−3.524**	**0.946**	**−3.724**	**0.0037**
**Rotigotine** –**Tryptamine**	**−2.968**	**0.919**	**−3.230**	**0.0211**
Rotigotine – Metanephrine	−2.179	0.900	−2.421	0.1896
**Flupenthixol** –**Phenethylamine**	**−3.678**	**0.986**	**−3.732**	**0.0036**
**Flupenthixol** –**Tyramine**	**−2.801**	**0.881**	**−3.181**	**0.0247**
Flupenthixol – Tryptamine	−2.244	0.851	−2.637	0.1149
Flupenthixol – Metanephrine	−1.456	0.833	−1.748	0.5838
Phenethylamine – Tyramine	0.877	0.973	0.901	0.9725
Phenethylamine – Tryptamine	1.433	0.948	1.512	0.7375
Phenethylamine – Metanephrine	2.222	0.944	2.353	0.2190
Tyramine – Tryptamine	−0.556	0.848	−0.656	0.9948
Tyramine – Metanephrine	−1.345	0.840	−1.600	0.6822
Tryptamine – Metanephrine	−0.788	0.810	−0.973	0.9598

Results are given on the log odds ratio (not the response) scale. All *p*-values are reported after adjustment for multiple comparisons using Tukey’s HSD criterion. Significant comparisons (*p* < 0.05) are in bold.

The time courses of larval movement between quartiles (Figure 3; Supplementary Figure 5) show that larvae started swimming quickly along the chamber until approximately 25 s into the experiment, as shown by the relatively steep positive slope of the line (Figure 3; Supplementary Figure 4). In all treatments, most larvae quickly move out of Q1 and spend little time in Q2 and Q3. The differences between treatments manifest largely in the probability of larvae being in Q4 through the course of the experiment. The results for the FSW control show that there was a low probability of remaining in Q1 and a high probability (0.75) of being in Q4 already by 25 s (Figure 3B). The largest differences in initial swimming speed were observed in response to the bilaterian trace amines and TAAR agonists, phenethylamine, tyramine and tryptamine, and the general bilaterian TAAR agonist, D,L-metanephrine hydrochloride. In all treatments, larvae moved away from the light more slowly in the first 25 s compared to the FSW control (Table 2; Figure 3; Supplementary Figure 4). Larvae in most treatments were essentially stationary by 25 s into the experiment, although there was evidence for backward movement through the chamber at later times, as shown by the slight negative slope beyond 30 s in some treatments (Supplementary Figure 4).

The DRD agonist rotigotine hydrochloride and the DRD antagonist flupenthixol dihydrochloride both had only a very mild effect on larval phototactic swimming behaviour compared to the FSW control, and indeed were similar to each other (Table 2; Figures 3B–D). The three bilaterian trace amines and TAAR-like agonists – phenethylamine, tyramine and tryptamine – had a larger effect on normal negative phototactic behaviour of larvae compared to the DRD agonist and antagonist, attenuating the phototactic response such that the probability of being observed in Q1 and Q4 is higher and lower, respectively (Table 2; Figures 3E–G). Treatment with the general bilaterian TAAR agonist D,L-metanephrine hydrochloride had a similar, albeit weaker, effect to that of the specific trace amines/TAAR agonists (Table 2; Figure 3H). Amongst the specific trace amines/TAAR agonists, phenethylamine and tyramine produced the strongest effect on larval behaviour, with both being strongly different from the FSW controls, but not from each other. Tryptamine and D,L-metanephrine hydrochloride also produced effects that were different from the FSW controls, but to a lesser extent than phenethylamine and tyramine; they were not different from each other. Larvae treated with phenethylamine and tyramine and then washed in FSW displayed normal, negative phototactic response to light (i.e., they were the same as FSW controls subjected to the same wash regime; Supplementary Figure 5).

## Discussion

Despite lacking a nervous system, the marine sponge *Amphimedon queenslandica* appears to be able to respond to bacterial-derived dopamine and trace amines, which are known to function as neurotransmitters and neuromodulators in other animals. This sponge has an estimated 130 rhodopsin class GPCRs (Rh-GPCRs), many of which comprise sponge-specific clades (Srivastava et al., 2010; Krishnan et al., 2015). Some genes in these clades are similar to bilaterian and cnidarian neurotransmitter receptors, including dopamine-like (DRD-like) and trace amine-like (TAAR-like) receptors (Krishnan et al., 2015). Consistent with this sponge being able to physiologically detect and respond to dopamine and trace amines, larval swimming behaviour is perturbed when in the presence of agonists and antagonists of these receptors.

### The potential for dopamine and trace amine signalling in the *Amphimedon* holobiont

The *A. queenslandica* genome lacks a gene encoding an aromatic L-amino acid decarboxylase (AADC) and thus appears incapable of decarboxylating L-DOPA and aromatic amino acids to produce dopamine and trace amines. The gene encoding this ancient enzyme is missing from other, but not all, demosponges (Riesgo et al., 2014; Francis et al., 2017; Kenny et al., 2020), suggesting it has been lost over the course of demosponge evolution. *A. queenslandica* also appears incapable of converting histidine into histamine via histidine decarboxylase (HDC). In contrast, two of three primary vertically-inherited bacterial symbionts in *A. queenslandica*, *AqS1* and *AqS2*, possess and express *aadc* and *hdc* genes (Fieth et al., 2016; Xiang et al., 2022), raising the possibility that these bacterially-derived monoamines can be used in signalling to their sponge host. Another neurotransmitter detected in sponges, serotonin, also appears to be produced by bacterial symbionts (Hedner et al., 2006; Leys, 2015).

The rhodopsin class GPCRs (Rh-GPCRs) appear to have been present in the shared ancestor of animals, fungi and other opisthokonts, and have independently expanded in sponges (de Mendoza et al., 2014; Krishnan et al., 2015). The *A. queenslandica* Rh-GPCR family does not include orthologues of bilaterian and cnidarian DRDs and TAARs (Srivastava et al., 2010; Krishnan et al., 2015), although, as we show here, this sponge appears to be sensitive to bilaterian dopamine and trace amine agonists and antagonists. Despite this lack of orthology, we identified two putative DRD (*AquDRD1-* and *AquDRD5-like*) and one putative TAAR-like (*AquTAAR-like*) genes in *A. queenslandica* genome based on sequence and transmembrane-loop similarities to bilaterian DRDs and TAARs. These sequence and structural similarities to bilaterian DRDs and TAARs probably evolved independently. TAARs have only been reported in vertebrates (Dieris et al., 2021), consistent with *AquTAAR-like* also not being an orthologue of the vertebrate gene. AquDRD1 is similar to the *Drosophila* Dop1R1 dopamine receptor, which is an orthologue of the vertebrate D1-like receptor (Karam et al., 2020; Silva et al., 2020). Amino acids located between TM2-TM4 are highly conserved between *A. queenslandica*, *Drosophila* and mammals, with all three having limited sequence similarities outside this region, consistent with AquDRD1-like being a functional dopamine receptor.

### DRD and TAAR-like agonists and antagonists affect larval phototaxis

To determine if *A. queenslandica* can be influenced by dopamine and trace amines potentially originating from its vertically-inherited bacterial symbionts, we subjected swimming larvae to known bilaterian agonists and antagonists of DRD and TAAR-likes. The expression of *AquDRD1*-, *AquDRD5-* and *AquTAAR-like* in posterior pole pigment ring cells and tissues, which are responsible for directing larvae away from the light (Leys and Degnan, 2001; Rivera et al., 2012), suggests that these agonists and antagonists can influence phototaxis via these receptors. Their relatively low expression levels are typical of functional GPCRs in other animals (Sriram et al., 2019), and the higher expression of *AquDRD1-like* in pigment ring cells suggests that signalling may be via this receptor. Importantly, this bioassay does not implicate dopamine and trace amines produced by the symbiotic bacteria or their putative sponge receptors in natural phototaxis. In this bioassay, modification of the stereotypic swimming behaviour only provides evidence that host sponge cells are competent to respond to these bacterial metabolites.

Bilaterian DRD and TAAR-like agonists and antagonists significantly weakened the ability of the larvae to swim away from light, consistent with the sponge being able to respond to dopamine and trace amines. The impact of both dopamine agonist and antagonist on phototaxis is markedly less than that of three trace amines, phenethylamine, tyramine and tryptamine, and of a general TAAR agonist, suggesting the *A. queenslandica* receptors expressed in the pigment ring cells are more sensitive to trace amines than dopamine. Although it is currently unknown if the signal transduction pathways are activated by these receptors in *A. queenslandica* larvae, conserved genes involved in GPCR signal transduction are significantly upregulated in these pigment ring cells compared to all other larval cell types. These genes include adenylate cyclase, phospholipase C, phosphodiesterases and guanylyl cyclase (Wong et al., 2022). In addition, agonists and antagonists of GPCR and calcium intracellular signalling pathways have similar effects as the DRD and TAAR-like agonists, consistent with these affecting sponge phototaxis through receptor-mediated pathways that are similar to bilaterian GPCR pathways (Wong et al., 2022). As further support for DRD and TAAR-like agonists and antagonists affecting *A. queenslandica* receptors, we exposed larvae to the two trace amines with the strongest affect, phenethylamine and tyramine, and then washed and re-exposed these larvae to the light gradient in FSW. These larvae exhibited normal negative phototaxis, suggesting that the trace amines are indeed interacting with a receptor, as their dilution abrogated their agonistic effects.

### An ancient role for monoamine signalling in metazoan holobionts

There are three ancient lineages of extant animals that appeared to have diverged over 700 million years ago, before the Cryogenian (Snowball Earth): the ctenophores; sponges; and parahoxozoans (bilaterians, cnidarians and placozoans) (Ryan et al., 2010; Simion et al., 2017; Erwin, 2020; Schultz et al., 2023). Minimally, their last common ancestor had a diversity of cell types that formed an integrated and homeostatic body plan with sensory cells and complex intercellular signalling to adjust cell states and physiologies to changing developmental and environmental conditions. It seems likely that this ancestor existed in a symbiotic relationship with microbes (that is, as a holobiont) and thus also had endogenous interkingdom signalling (McFall-Ngai et al., 2013).

Recognisable nervous systems exist in ctenophores and parahoxozoans, although neural cell structure and composition differs markedly between representatives of these lineages (Burkhardt and Jékely, 2021; Moroz et al., 2021; Moroz and Romanova, 2022; Burkhardt et al., 2023). Sponges have cell types that co-express proteins comprising macromolecular complexes in ctenophore and parahoxozoan synapses, and regulatory factors that have strong proneural activity in bilaterians (Sakarya et al., 2007; Richards et al., 2008; Conaco et al., 2012; Wong et al., 2019; Musser et al., 2021). Many of these proteins are also present in choanoflagellates and other holozoan relatives, although there appear to have been innovations along the bilaterian lineage in relation to monoamine signalling (Alié and Manuel, 2010; Burkhardt, 2015; Goulty et al., 2023). Together, this suggests that at least some of the chemical signalling used in neural synapses and aneural animals existed before the divergence of ctenophore, sponge and parahoxozoan lineages.

In humans and other animals, bacteria produce metabolites that are the same as neurotransmitters and neuromodulators, and can affect the host’s nervous, endocrine and immune systems (Carabotti et al., 2014; Mazzoli and Pessione, 2016; Klimovich and Bosch, 2018; Martin et al., 2018; Bathia and Bosch, 2020; Silva et al., 2020). The ability of bacterially derived dopamine and trace amines to affect *A. queenslandica* larval behaviour, potentially via Rh-GPCRs and their downstream signal transducers, indicates that these known neurotransmitters and neuromodulators can be interkingdom signals even in an animal without neurons. This raises the possibility that symbiont-host communication in stem metazoans contributed to the origin of the disparate nervous systems and aneural signalling systems present in the three basal lineages. This interkingdom signalling may have emerged as an outcome of another deeply ancient system for detecting extracellular signals, the innate immune system, which discriminates self from nonself, and symbionts from pathogens and food (Bosch, 2012; Hentschel et al., 2012; McFall-Ngai et al., 2013; Klimovich and Bosch, 2018). The origin of both innate immunity and neural signalling may trace back to early stem multicellular animals that relied on associated bacteria to regulate development and cell states, as observed in some extant choanoflagellates (Alegado and King, 2014; Woznica et al., 2016; Woznica and King, 2018).

## Data availability statement

The datasets presented in this study can be found in online repositories. The names of the repository/repositories and accession number(s) can be found in the article/Supplementary material.

## Author contributions

SD and BD conceptualized this project and the methodological strategies, except for statistical methods that were conceptualized by SB. XX and AV conducted all bioinformatic analyses and experimental assays, with assistance from HY. SB conducted all statistical analyses. AV, SD, BD, XX, and SB prepared the original draft of text and figures. BD and SD finalised the drafts. All authors read and approved the final manuscript.
