# Supplementary material for: Potential for host-symbiont communication via neurotransmitters and neuromodulators in an aneural animal, the marine sponge Amphimedon queenslandica
Source: Front Neural Circuits. 2023 Sep 29;17:1250694. doi: 10.3389/fncir.2023.1250694 (PMC10570526; doi:10.3389/fncir.2023.1250694)
Supplement: Supplementary file 4 [file Data_Sheet_1.pdf]

RHODOPSIN-LIKE GPCRS IN THE *Amphimedon queenslandica* GENOME

(Both vAq1 and vAq2.1 gene models are shown, based on Krishnan et al., 2015)

PUTATIVE DOPAMINE-LIKE RECEPTORS (BASED ON BLAST)

Aq1 >PAC:15700596 ID: 700596 Group: AqRho-E gene: Aqu1.202068

Blast Hit: D(1B) S: Mus musculus

scaffold:Aqu1:Contig5099:435:1619:-1

MILALIANGVVLVITIYQRKSWKQSSTIFFTSLILAHLVLTLYLPFSIAALAAGEWIIGDTDEEKKATCDFNGFIILC
CAYIIFMTLSLISTDRFLFIVKPHIHKRFMSPRVALVLVIIVWIVNAAFYSSGFIDGSGVVYQYVVWTFCFTRKFINN
QSMIVGESVYASKKKRLFGIFGSMLLVYGICFIPGIFFGTLLAIIDFPVVLGITALIFFFLAVVLSPVVQAYFRPEIN
SVIMLKRKPNPTTTSSSYISPSAALDEHCFHNSTLGIIVITVDHSYLSGLSPL

--> Aq2.1 Gene: Aqu2.1.03018 Scaffold Contig5099: 705-1,619

MILALIANGVVLVITIYQRKSWKQSSTIFFTSLILAHLVLTLYLPFSIAALAAGEWIIGDTDEEKKATCDFNGFIILC
CAYIIFMTLSLISTDRFLFIVKPHIHKRFMSPRVALVLVIIVWIVNAAFYSSGFIDGSGVVYQYVGKLGICYAFTTSL
GMAIFHFLLASLLLCIILVTSVWTFCFTRKFINNQSMIVGESVYASKKKRLFGIFGSMLLVYGICFIPGIFFGTLLAI
IDFPVVLGITALIFFFLAVVLSPVVQAYFRPEINSVIVNIFCRKMLKRKPNPTTTSSSYISPSAALDEHV

Aq1 >PAC:15704762 ID: 704762 Group: AqRho-E gene: Aqu1.206234

Blast Hit: D(1A) S:Didelphis virginiana

scaffold:Aqu1:Contig10729:4864:5633:-1

MSIPKEFLEAIINNILHFSHTGTLTLLYLPFNIIALAAGEWIFGSTDEEKRGFCSFTAYTLWYSIPVIVITLAAISFD
RFLFIVKPHLHKRFMRPWVALTLTIAIWILSAVTTFTPFIEGSGAVFMYEGSHGTCTVVILELQFAIVSFVGSLLVVG
IIIVTSVWTFCFARKFIHNQSEISGDNVYASKKKRLFGIFGAMLIVYGLCFTPGIINYAV

--> Aq2.1 Gene: Aqu2.1.09221 Scaffold Contig10729: 4,864-5,633

MSIPKEFLEAIINNILHFSHTGTLTLLYLPFNIIALAAGEWIFGSTDEEKRGFCSFTAYTLWYSIPVIVITLAAISFD
RFLFIVKPHLHKRFMRPWVALTLTIAIWILSAVTTFTPFIEGSGAVFMYEGSHGTCTVVILELQFAIVSFVGSLLVVG
IIIVTSVWTFCFARKFIHNQSEISGDNVYASKKKRLFGIFGAMLIVYGLCFTPGIINYAV

Aq1 >PAC:15710688 ID: 710688 Group: Between AqRho-C and D gene: Aqu1.212160

Blast Hit: D(2) S: Xenopus laevisscaffold:Aqu1:Contig12965:12342:13573:1
Dopamine-like 3

METVEAVEANFTFAQEFSSPVVAAVLIIXXXXANTFVLSITLYQRKSWKQSSTIFFTSLILANFVMVLLHFPFAVTAL
AAGEWIFGSTDEEKTGTCTFAALTFWYSSIVIILTLAAISFDRFLFIVKPLLHKRFMRPWVALTLTIVIWILSAVLSC
TPFYGLGNFGFTASIGLCIPLLVKGGFVILAFTILILSLLIIVITSLWTFFFTWWFLRKRSMVVESNIYSSKKRGLLG
IFGFMLLVYGISKGPRIIALVLVQFEVPFSSTSQIVIYFVYQLSIIGDPIVQSFFRPGLKQAMVSLFKNCKK

--> Aq2.1 Gene: Aqu2.1.18589 Scaffold Contig12965: 12,607-13,629

METVEAVEANFTFAQEFSSPVVAAVLIIXXXXANTFVLSITLYQRKSWKQSSTIFFTSLILANFVMVLLHFPFAVTAL
AAGEWIFGSTDEEKTGTCTFAALTFWYSSIVIILTLAAISFDRFLFIVKPLLHKRFMRPWVALTLTIVIWILSAVLSC
TPFYGLGNFGFTASIGLCIPLLVKGGFVILAFTILILSLLIIVITSLWTFFFTWWFLRKRSMVVESNIYSSKKRGLLG
IFGFMLLVYGISKGPRIIALVLVQFEVPFSSTSQIVIYFVYQLSIIGDPIVQSFFRPGLKQAMVSLFKNCKK

Aq1 >PAC:15710776 ID: 710766 Group: AqRho-E gene: Aqu1.212248

Blast Hit: D(1B) S: Mus musculus, Rattus norvegicus

scaffold:Aqu1:Contig12972:43001:44234:-1

MITVDCSNFSCSSSTFGELSLANEAEFSLVIERMERNYTFTGDFSPEAVAGVLSIEMILALIANGVVLVITIYQRKSW
KQSSTIFFTSLILAHLVLTLYLPFSIAALAAGEWIIGETDEDKEGTCHFNGFIILFSAYIVFMTLCLISIDRFLFIVK
PHLHKRFMSPRVALVLVIIVWIVNAAFYSAGFIDGSGIVYQYVVWTFCFTRKFINNQSMIVGESVYASKKKRLFGIFG
SMLLVYGICFIPAIFFGTLLAIIDFPVVLGITALIFFFLAVVLSPVVQAYFRPEINSVIVNIVCRKILKKKPNTLATS
STCNSSSAAVELRVSSLDV

--> Aq2.1 Gene: Aqu2.1.18711 Scaffold Contig12972: 43,001-44,234

MITVDCSNFSCSSSTFGELSLANEAEFSLVIERMERNYTFTGDFSPEAVAGVLSIEMILALIANGVVLVITIYQRKSW
KQSSTIFFTSLILAHLVLTLYLPFSIAALAAGEWIIGETDEDKEGTCHFNGFIILFSAYIVFMTLCLISIDRFLFIVK
PHLHKRFMSPRVALVLVIIVWIVNAAFYSAGFIDGSGIVYQYVVWTFCFTRKFINNQSMIVGESVYASKKKRLFGIFG
SMLLVYGICFIPAIFFGTLLAIIDFPVVLGITALIFFFLAVVLSPVVQAYFRPEINSVIVNIVCRKILKKKPNTLATS
STCNSSSAAVELRVSSLDV

Aq1 >PAC:15710944 ID: 710944 Group: AqRho-D gene:Aqu1.212416

Blast Hit: D(1) S:Drosophila melanogaster

scaffold:Aqu1:Contig12992:62266:63210:1

MESNFTFNGEFSGPAVAAVLTVVMILALIANGVVLSITLYQRKSWKQSSTIFFTSLILAHLVLNLLYLPFTIIALAAG
EWIFGSTDEEKTGTCIFVAFILWFGASVITITLAAISFDRFLFIVKPLLHKQFMRPWVALTLTIAIWILAAVLNFWPV
IHNFEHYSYNYELGYCTLVGVDIAAFIVFLVAIFFIVGTIFVTSLWTFCFTKSYFKAQSVIAGESVYASKKKRLFGVF
GSMLIVYGTSYLLTAFSFILQLFIVVPNEYYVMLTIAFPIVTIASPIIQSYFRPEIKSVLVSRCPLLFTCVCCSCVHS
IC

--> Aq2.1 Gene: Aqu2.1.18951 Scaffold Contig12992: 62,266-63,210

MESNFTFNGEFSGPAVAAVLTVVMILALIANGVVLSITLYQRKSWKQSSTIFFTSLILAHLVLNLLYLPFTIIALAAG
EWIFGSTDEEKTGTCIFVAFILWFGASVITITLAAISFDRFLFIVKPLLHKQFMRPWVALTLTIAIWILAAVLNFWPV
IHNFEHYSYNYELGYCTLVGVDIAAFIVFLVAIFFIVGTIFVTSLWTFCFTKSYFKAQSVIAGESVYASKKKRLFGVF
GSMLIVYGTSYLLTAFSFILQLFIVVPNEYYVMLTIAFPIVTIASPIIQSYFRPEIKSVLVSRCPLLFTCVCCSCVHS
IC

Aq1 >PAC:15711978 ID: 711978 Group: AqRho-B gene: Aqu1.213450

Blast Hit: D(1A)S: Xenopus laevis

scaffold:Aqu1:Contig13087:72248:73198:-1

MDVFGLNYTLAEDVNGPLLAVVLTLELIAALVTNTIVLAATLSQQKSLKLPSTILFTSLIMIHYVMAFIYILSWLISV
ISGGWIFGTSEEEKEATCNAAGFVVCYSLSVINATLTAISVDRWLFIVKPNFYKQYMKPKVTLVLVLSIWIFSGLTFI
TTFFGIGGFVFTTLGSCGPKFKDETGFTILLLAIFFPEISIVIVTSVWTYCFTKKFIREHAQLAENNVYVSKNRRLTG
IFGLMLIAYVICYVPSLIPIILNQFHDVPAMWIAFGLVCILAMTFINPIIQSFFRREVKEEIKKFCNIIKCCPHRSNA
IMAG

--> Aq2.1 Gene: Aqu2.1.20732 Scaffold Contig13087: 72,248-73,198

MDVFGLNYTLAEDVNGPLLAVVLTLELIAALVTNTIVLAATLSQQKSLKLPSTILFTSLIMIHYVMAFIYILSWLISV
ISGGWIFGTSEEEKEATCNAAGFVVCYSLSVINATLTAISVDRWLFIVKPNFYKQYMKPKVTLVLVLSIWIFSGLTFI
TTFFGIGGFVFTTLGSCGPKFKDETGFTILLLAIFFPEISIVIVTSVWTYCFTKKFIREHAQLAENNVYVSKNRRLTG
IFGLMLIAYVICYVPSLIPIILNQFHDVPAMWIAFGLVCILAMTFINPIIQSFFRREVKEEIKKFCNIIKCCPHRSNA
IMAG

Aq1 >PAC:15713944 ID: 713944 Group: AqRho-A gene: Aqu1.215416

Blast Hit: D(1B) S: Xenopus laevis

scaffold:Aqu1:Contig13214:61345:62064:-1

MLYFKACGYRLVLKNKEEESMDAVHNNFTLSPDINGPLLAAVISIEMIGGLIANSFVLILTICHIKTWKQPSTIFLTN
MLISNLLIVLFVMPFPVITSAFGEWIFGSTLDQKESVCKFTAIMSLFCVAVATEGLALLSFDRFLFIVRAFQYNRYMS
INKSLIAISISWAIAGIISIVPVLEFNVYEFAYSFWYLCSWFRGTGWFCSICFYLSSTASGEYHSNFSMDVHIYEEIS
QNKKD

--> Aq2.1 Gene: Aqu2.1.23882 Scaffold Contig13214: 61,209-62,064

MLYFKACGYRLVLKNKEEESMDAVHNNFTLSPDINGPLLAAVISIEMIGGLIANSFVLILTICHIKTWKQPSTIFLTN
MLISNLLIVLFVMPFPVITSAFGEWIFGSTLDQKESVCKFTAIMSLFCVAVATEGLALLSFDRFLFIVRAFQYNRYMS
INKSLIAISISWAIAGIISIVPVLEFNVYEFAYSFWYLCSWFRGTGWFCSICFYLSSTASGEYHSNFSMDVHIYEEIS
QNKKD

Aq1 >PAC:15713945 ID: 713945 Group: AqRho-A gene: Aqu1.215417

Blast Hit: D(5) S: Takifugu rubripes

scaffold:Aqu1:Contig13214:62301:63302:1

MEDENNFTLSGDINGPLLAAVISIEMIGGLIANSFVLILTICHIKTWKQPSTIFLTNMLISNLLIVLFVMPFAITTAA
SGEWLFGKTDKQKVKACQFTAFMFWFCVIVITEGLVLLSFDRFFYIVKSFEYERHMNRKISIIIVTLSWLLAALLTIP
PLFGLGRFGFSSSYGICVPRWEGEPGYVVYMLIVFIIFLLSIIITSSWTMIYTRRYLNMERQRLQLFDVNNDGNTNDI
YASRKRRVIGLFGMIMIVHLLCYLPSMIVALMELVTAPPPQLYATIFLLFLLLTVLSPLVQSFFRRDIRGTVVKLTGW
CSEVAKLFNKKYTRIASSSEN

--> Aq2.1 Gene: Aqu2.1.23883 Scaffold Contig13214: 62,301-63,363

MEDENNFTLSGDINGPLLAAVISIEMIGGLIANSFVLILTICHIKTWKQPSTIFLTNMLISNLLIVLFVMPFAITTAA
SGEWLFGKTDKQKVKACQFTAFMFWFCVIVITEGLVLLSFDRFFYIVKSFEYERHMNRKISIIIVTLSWLLAALLTIP
PLFGLGRFGFSSSYGICVPRWEGEPGYVVYMLIVFIIFLLSIIITSSWTMIYTRRYLNMERQRLQLFDVNNDGNTNDI
YASRKRRVIGLFGMIMIVHLLCYLPSMIVALMELVTAPPPQLYATIFLLFLLLTVLSPLVQSFFRRDIRGTVVKLTGW
CSEVAKLFNKKYTRIASSSEN

Aq1 >PAC:15714984 ID: 714984 Group: AqRho-E gene: Aqu1.216456

Blast Hit: D(1B) S: Mus musculus, Rattus norvegicus

scaffold:Aqu1:Contig13264:99897:100877:1

MERNYTFTGEFSPEAVAGVLSIEMILALIANVVVLVITFYQKKSFKHSSTIFFTSLILAHLALTLLYLPFTITSLFAG
EWIIGSTDDEKQGTCDFSAFMIITSAYIMYMTLSLISIDRFLFIVKPHLHKRFMSPRVALVLVIIVWIVNSIFFSSGF
IDESGIAYWYVAPLGICNALTISQAMAVIRFLVILILLGIIVVTSIWTFCFTRKFINNQSMIVGESVYASKKKRLFGI
FGSMLLVYGICFIPGAFLSSFLAILDAPGTLIISSLVFFILALVLSPVVQSYFRPEIKSVIVQICRKMMRKPNSTASK
NSAVTRDTNVDSVT

--> Aq2.1 Gene: Aqu2.1.25446 Scaffold Contig13264: 99,897-100,877

MERNYTFTGEFSPEAVAGVLSIEMILALIANVVVLVITFYQKKSFKHSSTIFFTSLILAHLALTLLYLPFTITSLFAG
EWIIGSTDDEKQGTCDFSAFMIITSAYIMYMTLSLISIDRFLFIVKPHLHKRFMSPRVALVLVIIVWIVNSIFFSSGF
IDESGIAYWYVAPLGICNALTISQAMAVIRFLVILILLGIIVVTSIWTFCFTRKFINNQSMIVGESVYASKKKRLFGI
FGSMLLVYGICFIPGAFLSSFLAILDAPGTLIISSLVFFILALVLSPVVQSYFRPEIKSVIVQICRKMMRKPNSTASK
NSAVTRDTNVDSVT

Aq1 >PAC:15714985 ID: 714985 Group: N/A gene: Aqu1.216457

Blast Hit: D(1B) S: Mus musculus, Rattus norvegicus; D(1)-like S: Oreochromis mossambicus

scaffold:Aqu1:Contig13264:104307:104864:1

MERNYTFTGDFSPEAVAGVLSIEMILALIANGVVLVITFYQKKSFKHSSTVFFTSLILAHLALTLLYLPFTITSFFAG
EWIIGSTDNEKQGTCDFTAFMIITSAYIMYMTLSLISIDRFLFIVKPHLHKRFMSPRVALVLVIIVWIVNSVFFSSGF

IDGSGIAYQYVAPLGVCNALTISQAMAII

--> Aq2.1 Gene: Aqu2.1.25447 Scaffold Contig13264: 104,307-104,864

MERNYTFTGDFSPEAVAGVLSIEMILALIANGVVLVITFYQKKSFKHSSTVFFTSLILAHLALTLLYLPFTITSFFAG
EWIIGSTDNEKQGTCDFTAFMIITSAYIMYMTLSLISIDRFLFIVKPHLHKRFMSPRVALVLVIIVWIVNSVFFSSGF
IDGSGIAYQYVAPLGVCNALTISQAMAII

Aq1 >PAC:15716338 ID: 716338 Group: AqRho-D gene: Aqu1.217810

Blast Hit: D(1B) S: Mus musculus, Rattus norvegicus

scaffold:Aqu1:Contig13317:148885:149805:1

MEGNFTFTGEFSGPAVAAVLTVEMILALIANGVVLSITLYQRKSLKQSSTIFFTSLILANLVLNLLCLPFNIIALAAG
EWIFGSTDEEKTATCIFAAFVYSYTIPVIFNTLAAISFDRFLFIVKPHLHKRFMRPWVALTLTIAIWILSAVYSFAPF
YGLNEYVYNEQYLLCYQHSNSIISVIMFIVFLPCVTTAIIVTSLWTFCFARSFLKDQSVIAGESVYASKKKRLFGIFG
SMLLIYGICVVPSLLLLAFQQFIDVPPELFICIRVGFFFITIASPIIQSYFRPEIKSALASCCPLLFYCVCC

--> Aq2.1 Gene: Aqu2.1.27388 Scaffold Contig13317: 148,885-149,805

MEGNFTFTGEFSGPAVAAVLTVEMILALIANGVVLSITLYQRKSLKQSSTIFFTSLILANLVLNLLCLPFNIIALAAG
EWIFGSTDEEKTATCIFAAFVYSYTIPVIFNTLAAISFDRFLFIVKPHLHKRFMRPWVALTLTIAIWILSAVYSFAPF
YGLNEYVYNEQYLLCYQHSNSIISVIMFIVFLPCVTTAIIVTSLWTFCFARSFLKDQSVIAGESVYASKKKRLFGIFG
SMLLIYGICVVPSLLLLAFQQFIDVPPELFICIRVGFFFITIASPIIQSYFRPEIKSALASCCPLLFYCVCC

Aq1 >PAC:15716428 ID: 716428 Group: AqRho-E gene: Aqu1.217900

Blast Hit: D(1B) S: Mus musculus, Rattus norvegicus

scaffold:Aqu1:Contig13321:92542:93519:-1

MKRNYTFTGDFSPEAVAGVLSIEMILALIANGVVLVITIYQKKSWKQSSTIFFTSLIMGNLLMTMSYLPFSIAALAAR
EWIIGSTDEEKQGTCDFTAFVMIYCGYVTLMTLSLISIDRFLFIVKPHLHKRFMSPRVALVLVIIVWIVNPLFFSSGF
INGSGIVFQYINNVGVCYAYSDSPIAAAFRLSLIIILLFIIVVTSVWTFCFTRKFINNQSMIVGESVYASKKKRLFGI
FGSMLLVYGICYIPGTFFNSSIAKANAPDKFNISALVLFLLALIVSPVVQSYFRPEINSAIVNIICRKMRKKRNRTTP
SNASKTNTNSFDL

--> Aq2.1 Gene: Aqu2.1.27513 Scaffold Contig13321: 92,542-93,519

MKRNYTFTGDFSPEAVAGVLSIEMILALIANGVVLVITIYQKKSWKQSSTIFFTSLIMGNLLMTMSYLPFSIAALAAR
EWIIGSTDEEKQGTCDFTAFVMIYCGYVTLMTLSLISIDRFLFIVKPHLHKRFMSPRVALVLVIIVWIVNPLFFSSGF
INGSGIVFQYINNVGVCYAYSDSPIAAAFRLSLIIILLFIIVVTSVWTFCFTRKFINNQSMIVGESVYASKKKRLFGI
FGSMLLVYGICYIPGTFFNSSIAKANAPDKFNISALVLFLLALIVSPVVQSYFRPEINSAIVNIICRKMRKKRNRTTP
SNASKTNTNSFDL

Aq1 >PAC:15716430 ID:716430 Group: AqRho-E gene: Aqu1.217902

Blast Hit: D(1B) S: Mus musculus, Rattus norvegicus; D(1)-like S: Oreochromis mossambicus

scaffold:Aqu1:Contig13321:95007:95984:-1

MERNYTFTGDFSPEAIAGVLSIEMILALIANGVVLVITIHQRKSWKQSSTIFFTSLVLGNLVMTTLYLPFTITSLAAG
EWIIGSTDEEKQGSCDFTAFVIIYCGYVMLMTLSLISIDRFLFIVKPHLHKRFMSPRVALVLVIIVWIVNAAFYSAGF
INGSGIEFQYINNIGICYAYATSPIAVVFRLSLIVILLSIILITSVWTFCFTRKFINNQSMIVGEGVYASKKKRLFGI
FGSMLLVYGICFIPGTFFSSSLAKVNAPDKLNISALVLFLLALILSPVVQSYFRPEINSVIVNIICRKIMKKRNRTTP
SNASKTNTNSFDL

--> Aq2.1 Gene: Aqu2.1.27515 Scaffold Contig13321: 95,007-95,984

MERNYTFTGDFSPEAIAGVLSIEMILALIANGVVLVITIHQRKSWKQSSTIFFTSLVLGNLVMTTLYLPFTITSLAAG
EWIIGSTDEEKQGSCDFTAFVIIYCGYVMLMTLSLISIDRFLFIVKPHLHKRFMSPRVALVLVIIVWIVNAAFYSAGF

INGSGIEFQYINNIGICYAYATSPIAVVFRLSLIVILLSIILITSVWTFCFTRKFINNQSMIVGEGVYASKKKRLFGI
FGSMLLVYGICFIPGTFFSSSLAKVNAPDKLNISALVLFLLALILSPVVQSYFRPEINSVIVNIICRKIMKKRNRTTP
SNASKTNTNSFDL

Aq1 >PAC:15716431 ID: 716431 Group: AqRho-E gene: Aqu1.217903

Blast Hit: D(1B) S: Mus musculus, Rattus norvegicus

scaffold:Aqu1:Contig13321:96385:97362:-1

MERNYTFTGDFSPEAVAGVLSIEMILALIANGVVLVITIYQRKSWKQSSTIFFTSLILGNLVMTMLYLPFSIAALAAR
EWIIGSTDEEKQGTCGLAAFVMIYCGYVMLLTLSLISIDRFLFIVKPHLHKRFMSPRVALVLVIIVWIVTAVLNSSGF
INGSGIEFQYIDNVGVCYAYPTSLIAAVFHLLLIVILLCIIVVTSVWTFCFTRKFINNQSMIVGESVYASKKKRLFGI
FGSMLLVYGICYIPGTFFTSSIAKANAPDKFNISALVLFLLALILSPIVQSYFRPEINSVIVNIICRKMRKKLDPITS
SNAIKTNTNSFDL

--> Aq2.1 Gene: Aqu2.1.27517 Scaffold Contig13321: 96,315-97,362

MERNYTFTGDFSPEAVAGVLSIEMILALIANGVVLVITIYQRKSWKQSSTIFFTSLILGNLVMTMLYLPFSIAALAAR
EWIIGSTDEEKQGTCGLAAFVMIYCGYVMLLTLSLISIDRFLFIVKPHLHKRFMSPRVALVLVIIVWIVTAVLNSSGF
INGSGIEFQYIDNVGVCYAYPTSLIAAVFHLLLIVILLCIIVVTSVWTFCFTRKFINNQSMIVGESVYASKKKRLFGI
FGSMLLVYGICYIPGTFFTSSIAKANAPDKFNISALVLFLLALILSPIVQSYFRPEINSVIVNIICRKMRKKLDPITS
SNAIKTNTNSFDL

Aq1 >PAC:15718484 ID: 718484 Group: AqRho-C gene: Aqu1.219956

Blast Hit: D(1) S: Drosophila melanogaster

scaffold:Aqu1:Contig13383:206185:207896:-1

MSFNSTDFVLTGDINSPTYAAALGIEGVIGIIANVAVLLMTLYQRKSWNQSSTIFFTSLLLSNLIIALWFLMSSIAVG
AEEWIFGNTFEEKNATCLVVAYLIWNCSIITMMTLAAISFDRFLFIVKPHLHKRFMRPRVALILIIGVWLLCSLINTT
PFYGFGVFMQDQAEIVGNNVYQSRRKRLFGIFGYMLISYLIALLPSYIDGAISMFYYLPACVRLGFVVTYGSFIISNP
IIQSYFRPEIVIIIKNLWNKIDDHGLVNFHPLDVRVDESLETLLLYIDNAIQYGEDIDVKVPKLEDDDNEDS

--> Aq2.1 Gene: Aqu2.1.30477 Scaffold Contig13383: 206,077-208,065

MSFNSTDFVLTGDINSPTYAAALGIEGVIGIIANVAVLLMTLYQRKSWNQSSTIFFTSLLLSNLIIALWFLMSSIAVG
AEEWIFGNTFEEKNATCLVVAYLIWNCSIITMMTLAAISFDRFLFIVKPHLHKRFMRPRVALILIIGVWLLCSLINTT
PFYGFGVFMQDQAEIVGNNVYQSRRKRLFGIFGYMLISYLIALLPSYIDGAISMFYYLPACVRLGFVVTYGSFIISNP
IIQSYFRPEIVIIIKNLWNKIDDHGLVNFHPLDVRVDESLETLLLYIDNAIQYGEDIDVKVPKLEDDDNEDS

Aq1 >PAC:15719539 ID: 719539 Group: AqRho-E gene: Aqu1.221011

Blast Hit: D(1B) S: Mus musculus, Rattus norvegicus, Homo sapiens

scaffold:Aqu1:Contig13412:154418:156225:1

MIDGQLNDKNNKLNKTSDLHLFTTSGDNIKHQWDLSIPSFSTACNLLRVHPYYIEQYKKTFQTEFSKDKCSFFNPEAV
AGVLSIEMILALIANAAGEWIIGNTDEEKQGTCGFTAYIILYCAYVMFMTLCLISIDRFLFIVKPHLHKRFMSPRVAL
VLVIIVWIVNALFFSSGFIDGSGVLFQYLDNSGACYAFTTSPFTAVIRFSLASIILCIIVVTSVWTFCFTLGESVYAS
KKKRLFGIFGSMLLVYGICFTPTFVNAPDKLIISAFILFFLAIILSPVVQSYFRPEINSVFVDIVCHKIMNKSNQTMS
SSRQFTIV

--> Aq2.1 Gene:Aqu2.1.31996 Scaffold Contig13412: 154,418-156,047

IDGQLNDKNNKLNKTSDLHLFTTSGDNIKHQWDLSIPSFSTACNLLRVHPYYIEQYKKTFQTEFSKDKCSFFNPEAVA
GVLSIEMILALIANAAGEWIIGNTDEEKQGTCGFTAYIILYCAYVMFMTLCLISIDRFLFIVKPHLHKRFMSPRVALV
LVIIVWIVNALFFSSGFIDGSGVLFQYLDNSGACYAFTTSPFTAVIRFSLASIILCIIVVTSVWTFCFTLGESVYASK
KKRLFGIFGSMLLVYGICFTPTFVNAPDKLIISAFILFFLAIILSPVVQSYFRPEINSVFVDIVCHKIMNKSNQTMSS
NSAVTQDLCIR SLNV

Aq1 >PAC:15723378 ID: 723378 Group: AqRho-C gene:Aqu1.224850

Blast Hit: D(1) S: Drosophila melanogaster

scaffold:Aqu1:Contig13481:358690:359643:-1

MDYNFTVTGNINGPVLAAVFAVEAVVGFIANIIVLSITLHQRKSFKQPSTIFFTSLLLSNLLDALVYLPMTTVATGAG
EWIYGSTFEVRRATCIFSGTSNWFIVFITIGVLAAISFDKCLFVTKPHFYKRFMKPWVALTVTVALWIIIAFILALPS
ALGASDNDYGFRYGACYITLEDHPAYIGFCSLIFSIFIITIIVTSIWTFCFTCKFIRDQSEIAGENVYHLKKMKLFGI
FGSMLLAYGICYGVSMTIGILSVSIAFPNEVHATSMVAFQLNIITTPLIQSYFRPDITKSMTSLKKFVQTQIKKLRYS
PSSPE

--> Aq2.1 Gene: Aqu2.1.37504 Scaffold Contig13481: 358,672-359,643

MDYNFTVTGNINGPVLAAVFAVEAVVGFIANIIVLSITLHQRKSFKQPSTIFFTSLLLSNLLDALVYLPMTTVATGAG
EWIYGSTFEVRRATCIFSGTSNWFIVFITIGVLAAISFDKCLFVTKPHFYKRFMKPWVALTVTVALWIIIAFILALPS
ALGASDNDYGFRYGACYITLEDHPAYIGFCSLIFSIFIITIIVTSIWTFCFTCKFIRDQSEIAGENVYHLKKMKLFGI
FGSMLLAYGICYGVSMTIGILSVSIAFPNEVHATSMVAFQLNIITTPLIQSYFRPDITKSMTSLKKFVQTQIKKLRYS
PSSPE

Aq1 >PAC:15728240 ID: 728240 Group: AqRho-C gene: Aqu1.229712

Blast Hit: D(1) S: Drosophila melanogaster

scaffold:Aqu1:Contig13521:7527:8483:-1

MSFNSTDFVLTGDINSTTYAAVLGIEGVIGMIVNVAVLLMTLYQRKSWNQSSTIFFTSLLLSNLIIALWYFMSSIAVG
AEEWIFGNTFEQKNATCLFIAYLFWNCSMNSMMTLAAISFDRFLFIVKPYLHKRFMRPRVALILIIGVWLLCSLINTT
PFCGFGVYRYYSPGGICSTSYETSGFFYLLFLVVVYSIIYFIIAFTSIWTFCFTRRFMQNQAELVDSSVYQSRRKRLF
GIFGYMLLSYIIAILISYITGIINMFYYLPSYVSLGVVVVYGSFIISNPIIQSYFRPEIVIIIKQWWNKIKRSSRNQD
RDLNYS

--> Aq2.1 Gene: Aqu2.1.43847 Scaffold Contig13521: 7,828-8,483

MSFNSTDFVLTGDINSTTYAAVLGIEGVIGMIVNVAVLLMTLYQRKSWNQSSTIFFTSLLLSNLIIALWYFMSSIAVG
AEEWIFGNTFEQKNATCLFIAYLFWNCSMNSMMTLAAISFDRFLFIVKPYLHKRFMRPRVALILIIGVWLLCSLINTT
PFCGFGVYRYYSPGGICSTSYETSGFFYLLFLVVHAEPG

PUTATIVE TRACE AMINE-LIKE RECEPTORS (BASED ON BLAST)

Aq1 >PAC:15700739 ID: 700739 Group: AqRho-C gene: Aqu1.202211

Blast Hits: TAAR(7e) S: Rattus norvegicus

scaffold:Aqu1:Contig5403:607:1542:1

MDYNFTATGSINGPVLAAVFAVEAVVGFIANIIVLSITLYQRKSFKQPSTIFFTSLILSDLLDVLVYLPMTTVSTGAE
EWIFGSTFEQRRATCVFSGIIFWFILYVTTGVLAAISFDKCLFITKPYFYKRFMKPWVALTITVALWIIIGLMFTLPL
ISFEDYYYYGIYFGPCYVGFLGKIIYSIMFGLWLIFIVTIIVTSVWTFCFTRRFINNQSAIGGENAYNSRKMKLFGIF
GSMLLAYTLCYAPAVIIGFISFGFALPYEVFATTTVALHFAPIVSPIIQAYFRPEITKSLVSFKNALCEKLRCSHSN

--> Aq2.1 Gene: Aqu2.1.03222 Scaffold Contig5403: 607-1,542

MDYNFTATGSINGPVLAAVFAVEAVVGFIANIIVLSITLYQRKSFKQPSTIFFTSLILSDLLDVLVYLPMTTVSTGAE
EWIFGSTFEQRRATCVFSGIIFWFILYVTTGVLAAISFDKCLFITKPYFYKRFMKPWVALTITVALWIIIGLMFTLPL
ISFEDYYYYGIYFGPCYVGFLGKIIYSIMFGLWLIFIVTIIVTSVWTFCFTRRFINNQSAIGGENAYNSRKMKLFGIF
GSMLLAYTLCYAPAVIIGFISFGFALPYEVFATTTVALHFAPIVSPIIQAYFRPEITKSLVSFKNALCEKLRCSHSN

Aq1 >PAC:15709352 ID: 709352 Group: AqRho-A gene: Aqu1.210824

Blast Hits: TAAR(7d) S: Mus musculus; TAAR(8a) S: Mus musculus; TAAR(8b) S: Mus musculus; TAAR(8c) S: Mus musculus; TAAR(8b) Rattus norvegicus

scaffold:Aqu1:Contig12760:28957:29979:-1

MDNINDNFTLSDVINGPLLAAAFATEMIGGLITNSLVLILTASHLKTWKQPTTIFLSNMLLNNLVIGICIIPFAIITA
AVGEWIFGRTEKEKETVCQVVGCIFTFTILTATESLVLLSFDRFFFITKSFQYNKYMTVNKALVIVSLSWALAVFLSI
LPFLGFGVHEFLSGVGLCVAGWNGQTAYAIVSFLVLCIFIGSIIVTSIWTLCFTRKHIKKTAIRTSIGAERSNHTQER
KVIGVFGMLIIVHLLCYAPIISFGLVESFINVLTPTAYAVAVFILFLLITLIPLVQSFFRSDIRTAIVKGSMTVIAFT
KRCRTTPHQISTVEMIRPSTPITSSSSI

--> Aq2.1 Gene: Aqu2.1.16444 Scaffold Contig12760: 28,957-29,979

MDNINDNFTLSDVINGPLLAAAFATEMIGGLITNSLVLILTASHLKTWKQPTTIFLSNMLLNNLVIGICIIPFAIITA
AVGEWIFGRTEKEKETVCQVVGCIFTFTILTATESLVLLSFDRFFFITKSFQYNKYMTVNKALVIVSLSWALAVFLSI
LPFLGFGVHEFLSGVGLCVAGWNGQTAYAIVSFLVLCIFIGSIIVTSIWTLCFTRKHIKKTAIRTSIGAERSNHTQER
KVIGVFGMLIIVHLLCYAPIISFGLVESFINVLTPTAYAVAVFILFLLITLIPLVQSFFRSDIRTAIVKGSMTVIAFT
KRCRTTPHQISTVEMIRPSTPITSSSSI

Aq1 >PAC:15712931 ID: 712931 Group: Not Clustered gene: Aqu1.214403

Blast Hit: TAAR (6) S: Pam troglodytes, Homo sapiens; Other 3 hits as 5-hydorxytryptamine receptor

scaffold:Aqu1:Contig13160:69230:70549:1

MDFFGSGDDGLNATSNYTTNYTDFDCLKSELDPNGITNRTFWSLRDTVVDGGIPSAVIQSLILAVALGWNLFIIVVFI
LKRELLKEPANILLFTLAIVDVLICLIVVPGPIVVTAANGEFVLGRNDQIRCAICDTQGFFFIFLTVLSVHTLALLSI
DRCILLSNPMKYPRYRKVGLWVLFIVLVWLLCLIISMPPAFNVGFGQWEFNRNFGLCLPRWIPPTYMGFVFLEGLIPI
TVIIITNIWTFKIILSFLKRKQVRRRESRLTSLSRKESEAEVQIKQQQKQLVQVFGALLIATIIAWVPLLTMLFVLIG
TDGEGVPDWIYLVCWFFYLINPLVHPILESLFVKELRTRIDKTKTNIRRASQSVMRLASRSTIKDIPEMSESTPYTKS
RVFSFSSNPYHSERKSDSLNGSIRNGSPPANDDNDNVFTPTSKVRFSIV

--> Aq2.1 Gene: Aqu2.1.22312 Scaffold Contig13160: 69,230-70,772

MDFFGSGDDGLNATSNYTTNYTDFDCLKSELDPNGITNRTFWSLRDTVVDGGIPSAVIQSLILAVALGWNLFIIVVFI
LKRELLKEPANILLFTLAIVDVLICLIVVPGPIVVTAANGEFVLGRNDQIRCAICDTQGFFFIFLTVLSVHTLALLSI
DRCILLSNPMKYPRYRKVGLWVLFIVLVWLLCLIISMPPAFNVGFGQWEFNRNFGLCLPRWIPPTYMGFVFLEGLIPI
TVIIITNIWTFKIILSFLKRKQVRRRESRLTSLSRKESEAEVQIKQQQKQLVQVFGALLIATIIAWVPLLTMLFVLIG
TDGEGVPDWIYLVCWFFYLINPLVHPILESLFVKELRTRIDKTKTNIRRASQSVMRLASRSTIKDIPEMSESTPYTKS
RVFSFSSNPYHSERKSDSLNGSIRNGSPPANDDNDNVFTPTSKVRFSIV

Aq1 >PAC:15713945 ID: 713945 Group: AqRho-A gene: Aqu1.215417

Blast Hit: TAAR (1) S: Homo sapiens; Other 2 hits as 5-hydorxytryptamine receptor and D(5) receptor; also recorded in Dopamine-like receptor part

scaffold:Aqu1:Contig13214:62301:63302:1

MEDENNFTLSGDINGPLLAAVISIEMIGGLIANSFVLILTICHIKTWKQPSTIFLTNMLISNLLIVLFVMPFAITTAA
SGEWLFGKTDKQKVKACQFTAFMFWFCVIVITEGLVLLSFDRFFYIVKSFEYERHMNRKISIIIVTLSWLLAALLTIP
PLFGLGRFGFSSSYGICVPRWEGEPGYVVYMLIVFIIFLLSIIITSSWTMIYTRRYLNMERQRLQLFDVNNDGNTNDI
YASRKRRVIGLFGMIMIVHLLCYLPSMIVALMELVTAPPPQLYATIFLLFLLLTVLSPLVQSFFRRDIRGTVVKLTGW
CSEVAKLFNKKYTRIASSSEN

--> Aq2.1 Gene: Aqu2.1.23883 Scaffold Contig13214: 62,301-63,363

MEDENNFTLSGDINGPLLAAVISIEMIGGLIANSFVLILTICHIKTWKQPSTIFLTNMLISNLLIVLFVMPFAITTAA
SGEWLFGKTDKQKVKACQFTAFMFWFCVIVITEGLVLLSFDRFFYIVKSFEYERHMNRKISIIIVTLSWLLAALLTIP
PLFGLGRFGFSSSYGICVPRWEGEPGYVVYMLIVFIIFLLSIIITSSWTMIYTRRYLNMERQRLQLFDVNNDGNTNDI
YASRKRRVIGLFGMIMIVHLLCYLPSMIVALMELVTAPPPQLYATIFLLFLLLTVLSPLVQSFFRRDIRGTVVKLTGW
CSEVAKLFNKKYTRIASSSEN

Aq1 >PAC:15716748 ID: 716748 Group: AqRho-C gene: Aqu1.218220

Blast Hit: TAAR(7b) S: Rattus norvegicus ; TAAR(7c) S: Rattus norvegicus

scaffold:Aqu1:Contig13334:5516:6466:-1

MSLFGNDSDGFGFTGNVNAPTYAAALAIEAVAGLVANFTVLAITLYQRNSFKQSSTIFFTSLLLANLIMVIYILITSI
TIGTEEWIVGSTHEEKTATCAFFAYMSWNSYMTMSMTIAAISFDRFLFIVKPHLHKSFMKPQVALFVTIGIWLLSGVI
NTTPFYGLGEYGYASYFGLCSSLLDRKGSNIAYLFIHAVIFMSIYASIFITSLWTFCFTRRFMQSQSEIARNSVYVSK
RKRLFGIFGTMLLAYAIALSPTYINSIVGVFYDIPLEVFVISCIVYNSITIINPIIQSYFRPEIKIVYKTIYTIIKTK
IRRQ

--> Aq2.1 Gene: Aqu2.1.28009 Scaffold Contig13334: 5,513-6,474

MSLFGNDSDGFGFTGNVNAPTYAAALAIEAVAGLVANFTVLAITLYQRNSFKQSSTIFFTSLLLANLIMVIYILITSI
TIGTEEWIVGSTHEEKTATCAFFAYMSWNSYMTMSMTIAAISFDRFLFIVKPHLHKSFMKPQVALFVTIGIWLLSGVI
NTTPFYGLGEYGYASYFGLCSSLLDRKGSNIAYLFIHAVIFMSIYASIFITSLWTFCFTRRFMQSQSEIARNSVYVSK
RKRLFGIFGTMLLAYAIALSPTYINSIVGVFYDIPLEVFVISCIVYNSITIINPIIQSYFRPEIKIVYKTIYTIIKTK
IRRQ

ALL OTHER RHODOPSIN-LIKE GPCRS

Aq1 >PAC:15699092 ID: 699092 scaffold:Aqu1:Contig1667:21:747:-1
gene:Aqu1.200564

MDDINNNFTLSEDVNGPLLAAVIGMEMLAGLITNSFVLILTACHLKNWKQPTTVFLSNMLANNLVVILFTMPLSIITT
ASGEWIFGSTVSQKESACYFAACIFIFSILTATESLVLLSFDRFFFIVKALQYKKYMTVNRAFIIVAVSWLLAAFLSM
LPFFGFGAFEFAFSYGMCVPGWRGQAGYAIFSFIVISIFIGSITVTSIWTMCFTRKYLKNAATNISTAASPGNPYAAQ
ERRVIGLF

→ Aq2.1 Gene: Aqu2.1.00826 Scaffold Contig1667: 55-747

MDDINNNFTLSEDVNGPLLAAVIGMEMLAGLITNSFVLILTACHLKNWKQPTTVFLSNMLANNLVVILFTMPLSIITT
ASGEWIFGSTVSQKESACYFAACIFIFSILTATESLVLLSFDRFFFIVKALQYKKYMTVNRAFIIVAVSWLLAAFLSM
LPFFGFGAFEFAFSYGMCVPGWRGQAGYAIFSFIVISIFIGSITVTSIWTMCFTRKYLKNAATNISTAASPGNPY

Aq1 >PAC:15699550 ID:699550 scaffold:Aqu1:Contig2858:61:666:1
gene:Aqu1.201022

MEDMDENFTLSGDINGPLLAAVISIEMIGGLIANSFVLILTICHIKTWKQPSTIFLTNMLISNILIVLFVMPFAITTA
ASDEWLFGKTYKQKMKVCQFTAFMFWFCKIVITEGLVLLSFDRFFYIVKSFEYERHMNQKISIIIVTLSWLLAALLTI
PPLFGLGRFSFSSSNGICVPHWEGESGYVVYMLIVFIIFIISLHN

→ Aq2.1 Gene: Aqu2.1.01517 Scaffold Contig2858: 61-666

MEDMDENFTLSGDINGPLLAAVISIEMIGGLIANSFVLILTICHIKTWKQPSTIFLTNMLISNILIVLFVMPFAITTA
ASDEWLFGKTYKQKMKVCQFTAFMFWFCKIVITEGLVLLSFDRFFYIVKSFEYERHMNQKISIIIVTLSWLLAALLTI
PPLFGLGRFSFSSSNGICVPHWEGESGYVVYMLIVFIIFIISLHN

Aq1 >PAC:15701137 ID:701137 scaffold:Aqu1:Contig6166:26:808:1
gene:Aqu1.202609

LAAEEWIFGSTDEEKRGTCYFGAFIFWWIVLVISLTLDAISLDRFLFIVKPHLHKRFMRPWVALTLTIAIWILSAVLS
IVHLFGFRLFIDICISSTANIGFVVYIILLSAIVLGIIFITSLWTFCFTRRFFKDQSVIAGESVYASKKKRLFGIFGS
MLLIYGICFVPGVLYFLLYLFIDVPLWLNIAATICFHFITVTNPIMQSYFRPEIKSIYIVLY

→ Aq2.1 Gene: Aqu2.1.03831 Scaffold Contig6166: 26-808

LAAEEWIFGSTDEEKRGTCYFGAFIFWWIVLVISLTLDAISLDRFLFIVKPHLHKRFMRPWVALTLTIAIWILSAVLS

IVHLFGFRLFIDICISSTANIGFVVYIILLSAIVLGIIFITSLWTFCFTRRFFKDQSVIAGESVYASKKKRLFGIFGS
MLLIYGICFVPGVLYFLLYLFIDVPLWLNIAATICFHFITVTNPIMQSYFRPEIKSIYIVLY

Aq1 >PAC:15701199 ID:701199 scaffold:Aqu1:Contig6273:29:625:1
gene:Aqu1.202671

MVLVISITLATISFDRFLFIVKPHLHKWVMRPWVALTLTIAIWILSAVLSIVHLFGFHLYIDICISSTADIGFVVYII
LLSAVVLGIIFITSLWTFCFTRRFFKDQSVIAGESVYASKKKRLFGIFGSMLLVYGICFVPAVLYFLLHLFIDVPLWL
NLAATICFLFITVANPVMQSYFRP EIKSVLFRCCPLKIYVCC

→ Aq2.1 Gene: Aqu2.1.03917 Scaffold Contig6273: 29-625

MVLVISITLATISFDRFLFIVKPHLHKWVMRPWVALTLTIAIWILSAVLSIVHLFGFHLYIDICISSTADIGFVVYII
LLSAVVLGIIFITSLWTFCFTRRFFKDQSVIAGESVYASKKKRLFGIFGSMLLVYGICFVPAVLYFLLHLFIDVPLWL
NLAATICFLFITVANPVMQSYFRP EIKSVLFRCCPLKIYVCC

Aq1 >PAC:15702185 ID:702185 scaffold:Aqu1:Contig7896:57:1878:-1
gene:Aqu1.203657

MERNYTFTGDFSPEAVAGVLSIEMILALIANGVVLVITIYQRKSWKQSSTIFFTSLILAHLALTLYLPFSIAALAARE
WVVGDTDEEKQGTCGFTAFVILFGIYVMLMTLSLISIDRFLFIVKPHLHKRFMSPRVALVLVIIVWSFDLLFFSTGFI
DGSGVEFRYIDYLGVCYAFTTSPVMAIFRFFFASLIFIIIIITSVWTFCFTRKFINNQSMIVGESVYASKKKRLFGIF
GLMMLVYGICVSPGIFFSTLLAIVDSPGVFTMTALMLFFLAVVLSPVIQAYFRPEINSVIVNIVCRNMLKKKPNPAAT
SIVVLSPVVQAYFRPEINSVIVNIAYHNILKKKPNSATTSSSSSAAVD LRVSSCT

→ Aq2.1 Gene: Aqu2.1.05392 Scaffold Contig7896: 57-1,878

MERNYTFTGDFSPEAVAGVLSIEMILALIANGVVLVITIYQRKSWKQSSTIFFTSLILAHLALTLYLPFSIAALAARE
WVVGDTDEEKQGTCGFTAFVILFGIYVMLMTLSLISIDRFLFIVKPHLHKRFMSPRVALVLVIIVWSFDLLFFSTGFI
DGSGVEFRYIDYLGVCYAFTTSPVMAIFRFFFASLIFIIIIITSVWTFCFTRKFINNQSMIVGESVYASKKKRLFGIF
GLMMLVYGICVSPGIFFSTLLAIVDSPGVFTMTALMLFFLAVVLSPVIQAYFRPEINSVIVNIVCRNMLKKKPNPAAT
SIVVLSPVVQAYFRPEINSVIVNIAYHNILKKKPNSATTSSSSSAAVD LRVSSCT

Aq1 >PAC:15702669 ID:702669 scaffold:Aqu1:Contig8595:983:2091:1
gene:Aqu1.204141

MEGNFTFTGEFSGPAVAAVLTVEMILALIANGVVLSITLFYQRKSWKQSSTIFFTSLILAHLVLNLLHLPFTITALAA
GEWIFGNTDEEKSGFCLFIAYTVWYIIFVISITLAAISFDRFLFIVKPHLHKRFMRPWVALTLTIAIWILSAVLCTGP
FIDNGRYSYDGELGFCTLIGFDIASLSLILPTIFLVVGTIIVTSFWTFCFTRRFFKAQSVIAGESVYASKKKRLIGIF
GSILLI YGTPVLLSSHTSGLRSKIGLVLLVPL

→ Aq2.1 Gene: Aqu2.1.06129 Scaffold Contig8595: 983-1,927

MEGNFTFTGEFSGPAVAAVLTVEMILALIANGVVLSITLFYQRKSWKQSSTIFFTSLILAHLVLNLLHLPFTITALAA
GEWIFGNTDEEKSGFCLFIAYTVWYIIFVISITLAAISFDRFLFIVKPHLHKRFMRPWVALTLTIAIWILSAVLCTGP
FIDNGRYSYDGELGFCTLIGFDIASLSLILPTIFLVVGTIIVTSFWTFCFTRRFFKAQSVIAGESVYASKKKRLIGIF
GSILLIYGTVYLFVALSLLLLFFITVPYEVYVTGYIIFCFVTIASPIIQSYFRPEIKSVLVSRCPL
LFSCVCCSCVHALC

Aq1 >PAC:15702875 ID:702875 scaffold:Aqu1:Contig8875:1923:2651:-1
gene:Aqu1.204347

MDFHTDGNDYFPDDFNGPLIAAALTVEFIGALVANGIVLIATLIQYKSFKVPSTMLFTSLIIIHLIISVLFVLLWIIS
AASEEWIYGTTIEQKIAACTFHGCVLNYGIWIIFGTITAISVDRCIFIVKPNFYKKFMKPKVTLTLIALIWVVALILD
TASLYGFGEVAYGEYGACVPRFEGEIIYVVITITLVIMKICIIIVTSIWTYCFTRKFIQEHSQLADDVYVSRNRRLIG
IFCAMLIG

→ Aq2.1 Gene: Aqu2.1.06430 Scaffold Contig8875: 1,923-2,651

MDFHTDGNDYFPDDFNGPLIAAALTVEFIGALVANGIVLIATLIQYKSFKVPSTMLFTSLIIIHLIISVLFVLLWIIS
AASEEWIYGTTIEQKIAACTFHGCVLNYGIWIIFGTITAISVDRCIFIVKPNFYKKFMKPKVTLTLIALIWVVALILD
TASLYGFGEVAYGEYGACVPRFEGEIIYVVITITLVIMKICIIIVTSIWTYCFTRKFIQEHSQLADDVYVSRNRRLIG
IFCAMLIG

Aq1 >PAC:15703005 ID:703005 scaffold:Aqu1:Contig9021:79:1038:1
gene:Aqu1.204477 →

MDIMSFNSTDFVLTGDINSPTYAAVLGIEGVIGIIVNVAVLLMTLYQRKSWNQSSTIFFTSLLLSNLIIALWYLISSI
AVGAEEWIFGNTFEEKNATCMLVAYIIWYGIMDISATLAAISFDRFLFIVKPYLHKRFMRPRVALILITGVWLLCSLI
NTIPFYSTGGYRYFPHSGICSFLNRTSAYFVVLIVVYAIIYSIIAFTSIWTFLFTRSFIKRQGRSVDRGVYQSKNKRL
FGIFGSMLLFYIIALLPSCIIIFSVFFDLPDGLYAFSICTYGLVAIINPLIQSYFRPEVKATLVLI
ASKIGLKSNKIGTSQATAG

→ Aq2.1 Gene: Aqu2.1.06625 Scaffold Contig9021: 79-1,038

MDIMSFNSTDFVLTGDINSPTYAAVLGIEGVIGIIVNVAVLLMTLYQRKSWNQSSTIFFTSLLLSNLIIALWYLISSI
AVGAEEWIFGNTFEEKNATCMLVAYIIWYGIMDISATLAAISFDRFLFIVKPYLHKRFMRPRVALILITGVWLLCSLI
NTIPFYSTGGYRYFPHSGICSFLNRTSAYFVVLIVVYAIIYSIIAFTSIWTFLFTRSFIKRQGRSVDRGVYQSKNKRL
FGIFGSMLLFYIIALLPSCIIIFSVFFDLPDGLYAFSICTYGLVAIINPLIQSYFRPEVKATLVLI
ASKIGLKSNKIGTSQATAG

Aq1 >PAC:15704497 ID:704497 scaffold:Aqu1:Contig10507:5186:5849:1
gene:Aqu1.205969

MILALIANGMVLSITLYQRKSWKQPSTIFFTSLILAHLVLNLLYLPFTIIALAAGEWIFGSTDEEKRGTCTFATFVFS
YTIPVIFNTLAAISFDPFLFIVKPHLHKQFMRSWIALTLTIDIWMFSALLNSTPFFGLNDEQFLLCYQHPNSIINLII
FCFLSMCYKSFFKDQSVIAAESVYASKKKRLFGVFGSMLLIYGICVVPI

→ Aq2.1 Gene: Aqu2.1.08851 Scaffold Contig10507: 5,186-5,725

MILALIANGMVLSITLYQRKSWKQPSTIFFTSLILAHLVLNLLYLPFTIIALAAGEWIFGSTDEEKRGTCTFATFVFS
YTIPVIFNTLAAISFDPFLFIVKPHLHKQFMRSWIALTLTIDIWMFSALLNSTPFFGLNDEQFLLCYQHPNSIINLII
FCFLSMCYKYIYHCDIIMDLLFH

Aq1 >PAC:15704842 ID:704842 scaffold:Aqu1:Contig10790:381:1325:-1
gene:Aqu1.206314

MEGNFSYVGDFNAPAVAAVLTVEMILALIANGVVLSITLYQRKSWKQSSTIFFTSLILAHLVLNLLYLPFTIIALAAG
EWIFGSTDEEKRGFCSFNAYTLSYSGFVISMTLAAVSFDLFLFIVKPHLHKRFMRPRWVALTLVIAIWILSAVLGAGP
FLDIGHYSYDDKSGFCSLIGFDFAAFVVILVIISLVVGTIFVTSLWTFCFTRSFFKAQSVIAGESVYASKKKRLIGIF
GSMLLIYGTCSLFVASGYLLQFFIFPPYEIYVTGYIVSYFVTVASPIIQSYFRPEIKSVLVSCCPL
LFTCVCCSFVHAVC

→ Aq2.1 Gene: Aqu2.1.09317 Scaffold Contig10790: 381-1,325

MEGNFSYVGDFNAPAVAAVLTVEMILALIANGVVLSITLYQRKSWKQSSTIFFTSLILAHLVLNLLYLPFTIIALAAG
EWIFGSTDEEKRGFCSFNAYTLSYSGFVISMTLAAVSFDLFLFIVKPHLHKRFMRPRWVALTLVIAIWILSAVLGAGP
FLDIGHYSYDDKSGFCSLIGFDFAAFVVILVIISLVVGTIFVTSLWTFCFTRSFFKAQSVIAGESVYASKKKRLIGIF
GSMLLIYGTCSLFVASGYLLQFFIFPPYEIYVTGYIVSYFVTVASPIIQSYFRPEIKSVLVSCCPL
LFTCVCCSFVHAVC

Aq1 >PAC:15704843 ID:704843 scaffold:Aqu1:Contig10790:4493:5437:-1
gene:Aqu1.206315

MEGNFTFTGDFNTPAVAAVFTVEMILALIANGVVLSITLYQRKSWKQSSTIFFTSLILAHLVLNLLCLPFTIIALAAG
EWIFGSTDEEKRWTCIFSAFILWFGAYVITITLAAISFDRFLFIVKPHLHKRFMRPWVALTLTIAIWTLSVSVFGSLQ
FIGIGHYSYDDELGYCTLIDVDVASFVVILVIIFLVVVTIFITSLWTFCFTRSFFKDQSVIAGESVYASKKKRLFGIF
GSMLLIYGTSYLFLALGFILQALIFLPYELYITYYIVFCFVTIASPIIQSYFRPEIKSVLVSRCPL
LFTCVCCSCVHAVC

→ Aq2.1 Gene: Aqu2.1.09318 Scaffold Contig10790: 773-5,437

MEGNFTFTGDFNTPAVAAVFTVEMILALIANGVVLSITLYQRKSWKQSSTIFFTSLILAHLVLNLLCLPFTIIALAAG
EWIFGSTDEEKRWTCIFSAFILWFGAYVITITLAAISFDRFLFIVKPHLHKRFMRPWVALTLTIAIWTLSVSVFGSLQ
FIGIGHYSYDDELGYCTLIDVDVASFVPLL

Aq1 >PAC:15705622 ID:705622 scaffold:Aqu1:Contig11404:790:1518:1
gene:Aqu1.207094

MEGNFTFTGDFNTLAVAAVLTVEMILALIANGVVLSITLYQRKSWKQSSTIFFTSLILAHLVLNLLYLPFAIIALAAG
EWIFGSTDEEKIGTCTSAAFVYSFTIPVIFMTLAAISFDRFLFIVKPHLHKRFMRPWVALALTIVIWILSAVFSSAPL
YGINEYVYEDQFLLCYQRPNGTMSLIIIFVFLPCVTAVIIVVTSLWTFCFARSFFKDQSVVAGESVYEALGSTDPHHC
YVAVCSCI

→ Aq2.1 Gene: Aqu2.1.10549 Scaffold Contig11404: 790-1,583

MEGNFTFTGDFNTLAVAAVLTVEMILALIANGVVLSITLYQRKSWKQSSTIFFTSLILAHLVLNLLYLPFAIIALAAG
EWIFGSTDEEKIGTCTSAAFVYSFTIPVIFMTLAAISFDRFLFIVKPHLHKRFMRPWVALALTIVIWILSAVFSSAPL
YGINEYLYLALYISLHSTLTLILTCVICYR

Aq1 >PAC:15706124 ID:706124 scaffold:Aqu1:Contig11695:9419:10501:1
gene:Aqu1.207596

MSISSENNFTINDDVNGPVLAAVFATEMILALIANGAVLLITITQRNSWKQSSTIFFTSLILAHLVPNLLYLPFTIIS
LAAGEWIFGSTDEEKRGFCSFIAYTLWYSIPVITITLAAISFDRFLFIVKPHLHKRFMRPWVALTLTLAIWILSAVTT
FTPFIEGSGAVFMYEGSHGTCTVVILELHFAIFSFVGSLLVVGIITVTSVWTFCFTRKFIHNQSEIAGDHVYASKKKR
LFGIFGAMLIVYGLCFTPGIINYVVVRAIVVADEIYLAVVISFIFVTVLSPIVQSYFRPEIKTKIVFISRKILQKPPQ
RDSIDYLGNSNNDSSGSKTEINQIRTSTQLKISSDHVLNTEHLSTTVV

→ Aq2.1 Gene: Aqu2.1.11330 Scaffold Contig11695: 9,419-10,501

MSISSENNFTINDDVNGPVLAAVFATEMILALIANGAVLLITITQRNSWKQSSTIFFTSLILAHLVPNLLYLPFTIIS
LAAGEWIFGSTDEEKRGFCSFIAYTLWYSIPVITITLAAISFDRFLFIVKPHLHKRFMRPWVALTLTLAIWILSAVTT
FTPFIEGSGAVFMYEGSHGTCTVVILELHFAIFSFVGSLLVVGIITVTSVWTFCFTRKFIHNQSEIAGDHVYASKKKR
LFGIFGAMLIVYGLCFTPGIINYVVVRAIVVADEIYLAVVISFIFVTVLSPIVQSYFRPEIKTKIVFISRKILQKPPQ
RDSIDYLGNSNNDSSGSKTEINQIRTSTQLKISSDHVLNTEHLSTTVV

Aq1 >PAC:15706178 ID:706178 scaffold:Aqu1:Contig11720:1222:2157:1
gene:Aqu1.207650

EGNFTFTGEFSGPGVAAVLIVEMILALIANGVVLSITLYQRKSWKQSSTIFFTSLILAHLVLNLLYLPFAIIALAAGE
WIFGSTDEEKRGTCTSAAFVYSFTIPVIFMTLAAISFDRFLFIVKPHLHKRFMRPWVALTLTIAIWILSAVFGTLPFF
DIGQYSYGDELGYCTLIGVDIAAFVVILVIIFLVVGTIIVTSLWTFCFTRKFFKAQSVIAGESLYASKKKRLIGIFGS
MLLIYGTVYLIVALNFLLRIFIFLPYEFNVTGYIVFCFVTIASPIIQSYFRPEIKSVLVSRCPLLF TCACCSCVHAV

→ Aq2.1 Gene: Aqu2.1.11417 Scaffold Contig11720: 1,288-8,725

MILALIANGVVLSITLYQRKSWKQSSTIFFTSLILAHLVLNLLYLPFAIIALAAGEWIFGSTDEEKRGTCTSAAFVYS
FTIPVIFMTLAAISFDRFLFIVKPHLHKRFMRPWVALTLTIAIWILSAVFGTLPFLDFGHYSYDDELGYCTLVGVDIP
AFVAILVIVFLVVGTIFVTSLWTFCFTRSYFKAHYQYDWKLEEAQKNILQTHTTAVSTRMLYKLGQQMGKEFTPVEYF
SIDRVFRNKTLDATHLAKFHQIEGVVADYNLTLGDLMGVLYTFFSKMVIAKC

Aq1 >PAC:15706179 ID: 706179 scaffold:Aqu1:Contig11720:4382:5323:1 gene:Aqu1.207651

MAENFTFTGEFSSPAVAAVLTVVMILALIANGVVLSITLYQRKSWKQSSTIFFTSLILAHLVLNLLYLPFTIIALAAG
EWIFGSTDEEKTATCYFAAWMQWSGGSVLALTLAAISFDRFLFIVKPHLHKRFMRPWVALTLTIAIWILSAVIGTLPF
LDFGHYSYDDELGYCTLVGVDIPAFVAILVIVFLVVGTIFVTSLWTFCFTRSYFKAQSVIAGESVYASKKKRLFGVFG
SMLLIYGTVYFSTALNILLQIFIFLPFEFNVTNYIVYFMVTIASAIIQSYFRPEIKSVLVSHCPLL
FTCVCCSCVHAVC

→ Aq2.1 Gene: Aqu2.1.11418 Scaffold Contig11720: 4,382-5,323

MAENFTFTGEFSSPAVAAVLTVVMILALIANGVVLSITLYQRKSWKQSSTIFFTSLILAHLVLNLLYLPFTIIALAAG
EWIFGSTDEEKTATCYFAAWMQWSGGSVLALTLAAISFDRFLFIVKPHLHKRFMRPWVALTLTIAIWILSAVIGTLPF
LDFGHYSYDDELGYCTLVGVDIPAFVAILVIVFLVVGTIFVTSLWTFCFTRSYFKAQSVIAGESVYASKKKRLFGVFG
SMLLIYGTVYFSTALNILLQIFIFLPFEFNVTNYIVYFMVTIASAIIQSYFRPEIKSVLVSHCPLL
FTCVCCSCVHAVC

Aq1 >PAC:15709198 ID:709198 scaffold:Aqu1:Contig12731:6409:7389:-1 gene:Aqu1.210670

MDYNFTATDLDNFTATGSINGPVLAAVFAVEAVVGFIANIIVLSITLYQRKSFRQPSTIFFTSLILSNLLDALVYLPM
TTVATGAEEWIFGSTFEVRRATCVFSGIINWFIIFITVAVLAAISFDKCLFIVKPHFYKRFMKPWVTLIITVALWLIV
ALIFILPSLGFGEYDYHFEYGPCYITFKKNIAFVLFYIATVLIFIIVIIVTSIWTFCFTRRFIRDQSAIAGENVYHLK
KMKLFGIFGSMLLAYGICYGVSFTIGIVSLGVTFPHEVYATGVFVFQFSIIATPLIQSYFRPDIVD
SLKSFKNFVYTQMKKLQYSLPTPSQT

→ Aq2.1 Gene: Aqu2.1.16173 Scaffold Contig12731: 6,323-7,426

MDYNFTATDLDNFTATGSINGPVLAAVFAVEAVVGFIANIIVLSITLYQRKSFRQPSTIFFTSLILSNLLDALVYLPM
TTVATGAEEWIFGSTFEVRRATCVFSGIINWFIIFITVAVLAAISFDKCLFIVKPHFYKRFMKPWVTLIITVALWLIV
ALIFILPSLGFGEYDYHFEYGPCYITFKKNIAFVLFYIATVLIFIIVIIVTSIWTFCFTRRFIRDQSAIAGENVYHLK
KMKLFGIFGSMLLAYGICYGVSFTIGIVSLGVTFPHEVYATGVFVFQFSIIATPLIQSYFRPDIVD
SLKSFKNFVYTQMKKLQYSLPTPSQT

Aq1 >PAC:15709199 ID:709199 scaffold:Aqu1:Contig12731:7869:8867:-1 gene:Aqu1.210671

MDYNFTATDLDNFTATGSINGPVLAAVFALEAVVGFIANIIVLSITLYQRKPFKQPSTIFFTSLILSNLLDALVYLPM
TTVATGAEEWIFGSTFEQRKATCLFSGVIFWFILYVTVAVLAAISFDRCLFIVKPYFYKRFMKPWVALIIAVIPWMII
ALIMMLPFVGFGEYGYQFDYGPCYITFENNMGFVIFRFATLFTFIIVIIVTSIWTFCFTRRFIRDQSAIAGESVYHVK
KMKLFGIFGSMLLAYVICYGVSITIGIISAAVTFPHEVYATGMFVFQFSIIATPLIQSYFRPDIVD
SLKSFKNFVYTQIKRQRYSPPSPIRLQTQHKF

→ Aq2.1 Gene: Aqu2.1.16174 Scaffold Contig12731: 7,747-8,867

MDYNFTATDLDNFTATGSINGPVLAAVFALEAVVGFIANIIVLSITLYQRKPFKQPSTIFFTSLILSNLLDALVYLPM
TTVATGAEEWIFGSTFEQRKATCLFSGVIFWFILYVTVAVLAAISFDRCLFIVKPYFYKRFMKPWVALIIAVIPWMII
ALIMMLPFVGFGEYGYQFDYGPCYITFENNMGFVIFRFATLFTFIIVIIVTSIWTFCFTRRFIRDQSAIAGESVYHVK
KMKLFGIFGSMLLAYVICYGVSITIGIISAAVTFPHEVYATGMFVFQFSIIATPLIQSYFRPDIVD
SLKSFKNFVYTQIKRQRYSPPSPIRLQTQHKF

Aq1 >PAC:15709200 ID:709200 scaffold:Aqu1:Contig12731:9312:10292:-1 gene:Aqu1.210672

MDYNFPAIDFDNFTATGSVNGPVLAAVFAVEAVVGFIANIIVLSITLYQRKSFRQPSTIFFTSLILSNLLDALVYLPM
TTVATGAEEWIFGSTFEQKKATCVFSGIIFWFILYVTAAMLAAISFDKCLFIVKPYFYKRFMKPWVALIITVIPWMIT
TLIMILPLVGFGEYGYHFAYGPCYATFEKNMVFVIFRFATLFIFIIVIIVTSIWTFCFTRRFIRDQSAIAGENVYHLK
KMKLFGIFGSMLLAYGTCYGASFTIGIISLTVTFSEEVHATGVFILQLNIIATPLIQSYFRPDIVN
SLKSFKNSVYTQMKKLQYSPPTPSPT

→ Aq2.1 Gene: Aqu2.1.16175 Scaffold Contig12731: 9,312-10,292

MDYNFPAIDFDNFTATGSVNGPVLAAVFAVEAVVGFIANIIVLSITLYQRKSFRQPSTIFFTSLILSNLLDALVYLPM
TTVATGAEEWIFGSTFEQKKATCVFSGIIFWFILYVTAAMLAAISFDKCLFIVKPYFYKRFMKPWVALIITVIPWMIT
TLIMILPLVGFGEYGYHFAYGPCYATFEKNMVFVIFRFATLFIFIIVIIVTSIWTFCFTRRFIRDQSAIAGENVYHLK
KMKLFGIFGSMLLAYGTCYGASFTIGIISLTVTFSEEVHATGVFILQLNIIATPLIQSYFRPDIVN
SLKSFKNSVYTQMKKLQYSPPTPSPT

Aq1 >PAC:15710365 ID:710365 scaffold:Aqu1:Contig12925:43645:44373:-1
gene:Aqu1.211837

MHFHTDGNYSFPEDFNGPLIAAALTVEFIGALVANGIVLIATLIQYKSLKVLSTMLFTSLIIIHLIISVLFILLWIIS
AASEEWIYGTTIEQKIAACTFHGCVLNYGIWIIFGTITAMSVDHCIFIVKPNFYKRFMKPKVTLTLIALIWVVALILD
TAPLYGFGEVAYEEYGACVPRFEGEITYVVITMTLVIMQICIIIVTSTWIYCFTHKFIQEHSQLADDVYVSRNRRLVG
IFCAMLIG

→ Aq2.1 Gene: Aqu2.1.18080 Scaffold Contig12925: 43,361-44,373

MHFHTDGNYSFPEDFNGPLIAAALTVEFIGALVANGIVLIATLIQYKSLKVLSTMLFTSLIIIHLIISVLFILLWIIS
AASEEWIYGTTIEQKIAACTFHGCVLNYGIWIIFGTITAMSVDHCIFIVKPNFYKRFMKPKVTLTLIALIWVVALILD
TAPLYGFGEVAYEEYGACVPRFEGEITYVVITMTLVIMQICIIIVTSTWIYCFTHKFIQEHSQLADDVYVSRNRRLVG
IFCAMLIG

Aq1 >PAC:15710682 ID:710682 scaffold:Aqu1:Contig12965:2:839:1
gene:Aqu1.212154

IANGVVLSITLYQRKSWKQSSTIFFTSLILAHLVLNLLYFPFTIIALAAGEWIFGSTDEEKRGTCSFCSFTALCILLI
IGMTVAAISFDRFLFIVKPHLHKRFMRPWVALTLTIAIWTLSAVLSSTPFYGLGNFSFDELLGFCFPLWISVDFIVYS
LVIILLLFSVIIVTSVWTLCFTHRFITQQSQLSNDNIYASKKKWLLGIFGSMLLVYGIYFMPGVIVVILIPLIDVPIA
LIVASIICSFFITVANPIVQSYFRPEIKSAFLSLFKKPSPFVSV

→ Aq2.1 Gene: Aqu2.1.18582 Scaffold Contig12965: 243-1,893

MTVAAISFDRFLFIVKPHLHKRFMRPWVALTLTIAIWTLSAVLSSTPFYGLGNFSFDELLGFCFPLWISVDFIVYSLV
IILLLFSVIIVTSVWTLCFTHRFITQQSQLSNDNIYASKKKWLLGIFGSMLLVYGIYFMPVANPIVQSYFRPEIKSVV
YFYGPMHLNNFKKDKVKRVWIAINCNFTGSFNCPITIAILSITTLAVLIANSVVLFITLYQRKSLKQSSTIFFTSLIL
VRLVLNLLYLPFTIIALAAAFVITITLAAKSFDRFPFIVKPHLYKRFMRPWVALTLTIAIWILSAV
LEQYLSLQRLIGPFVSLEGSSKLSQ

Aq1 >PAC:15710683 ID:710683 scaffold:Aqu1:Contig12965:2968:3888:1
gene:Aqu1.212155

MENKFTFTGEFSGPAVAAVLTVEMILALIANGVVLSITLYQRKSWKQSSTIFFTSLILAHLVLNVLYLPFTIIALAAG
EWIFGSTDDEKTRTCSFCSFTALCILLIIVMTVAAISFDRFLFIVKPHLHKQFMRPWVALTLTIAIWTLSAVLSSTPF
YGLGNFNFDELLGFCFPLWISVDFIVYSLIIALLLFSVIIVTSVWTLCFTHRFITQQSQLSNDDTYGSKKKWLLGIFG
SMLLVYGICFLPSVIIVILIPLIDVPIALVISSIICSLFITVANPIVQSYFRPEIKSAFLSLFKRPSPFVSV

→ Aq2.1 Gene: Aqu2.1.18583 Scaffold Contig12965: 2,968-4,777

MENKFTFTGEFSGPAVAAVLTVEMILALIANGVVLSITLYQRKSWKQSSTIFFTSLILAHLVLNVLYLPFTIIALAAG
EWIFGSTDDEKTRTCSFCSFTALCILLIIVMTVAAISFDRFLFIVKPHLHKQFMRPWVALTLTIAIWTLSAVLSSTPF
YGLGNFNFDELLGFCFPLWISVDFIVYSLIIALLLFSVIIVTSVWTLCFTHRFITQQSQLSNDDTYGSKKKWLLGIFG
SMLLVYGICFLPIANPIVQSYFRPEIKTALIANSVVLFITLYQKKSWKQSSTIFFTSLILAHLVLNLLLWFGASVITI
TLAAISFDQFFFIVKPHLHKQFMRPWVALTLTIAIWILSAVLSSGPFS VYIK

Aq1 >PAC:15710684 ID:710684 scaffold:Aqu1:Contig12965:6737:7678:1

gene:Aqu1.212156

METVEAVEANFTFAQEFSSPVVAAVLIIQVILALIANGVVLSITLYQRKSWKQSSTIFFTSLILAHLVLNLLYLPFTI
IALAAGEWIFGSTDEEKRGTCTFAAFVFVSSILAIFMTLAAISFDRFLFIVKPHLHKRFMRPWVALTLTIAIWTLSAV
LSFTPFFGFGHFLYFSRIGSCFPIWATLEYELYTLALVATNLLIIAVTSVLTFCFTRRFINNQSLITPNVYSCEKKRL
FGIFGAMLLSYVVCFIPSVILIFLFLFPFTMPVEYIVTCFVSVLFVTTVNPLIQSYFRPEVKAIIN
SMFFQKLSKKILV

→ Aq2.1 Gene: Aqu2.1.18584 Scaffold Contig12965: 6,579-7,719

METVEAVEANFTFAQEFSSPVVAAVLIIQVILALIANGVVLSITLYQRKSWKQSSTIFFTSLILAHLVLNLLYLPFTI
IALAAGEWIFGSTDEEKRGTCTFAAFVFVSSILAIFMTLAAISFDRFLFIVKPHLHKRFMRPWVALTLTIAIWTLSAV
LSFTPFFGFGHFLYFSRIGSCFPIWATLEYELYTLALVATNLLIIAVTSVLTFCFTRRFINNQSLITPNVYSCEKKRL
FGIFGAMLLSYVVCFIPSVILIFLFLFPFTMPVEYIVTCFVSVLFVTTVNPLIQSYFRPEVKAIIN
SMFFQKLSKKILV

Aq1 >PAC:15710685 ID:710685 scaffold:Aqu1:Contig12965:7829:8776:1
gene:Aqu1.212157

MEDNFTFTGNFSTPAVAAVLSVETVAGLVANFIILSITLYQRKSLKQPSTIFFTSLILALLVLLLVYLPLSIIAIAAE
EWIFGSSFEEKSATCSFAAYIFWYCLMVITLTLDAISFDRFLFIVKPRLHKRFMRPWVALTLTIAIWILSAILNSAPF
FGLGVFRYESWFGSCIPVWISIDFVAYSLTIIFFLLSVIVITSLWTFCFTRQFMNEQSLSTTPDAYTSQKKRLFGIFG
SMLIVYGICFTPGTIMIILIPIVDSPAELFVTSYVCILFITVANPIVQSYFRPEIKSTLLALCQRK
QANQFQSLELSKHSM

→ Aq2.1 Gene: Aqu2.1.18585 Scaffold Contig12965: 7,829-8,827

MEDNFTFTGNFSTPAVAAVLSVETVAGLVANFIILSITLYQRKSLKQPSTIFFTSLILALLVLLLVYLPLSIIAIAAE
EWIFGSSFEEKSATCSFAAYIFWYCLMVITLTLDAISFDRFLFIVKPRLHKRFMRPWVALTLTIAIWILSAILNSAPF
FGLGVFRYESWFGSCIPVWISIDFVAYSLTIIFFLLSVIVITSLWTFCFTRQFMNEQSLSTTPDAYTSQKKRLFGIFG
SMLIVYGICFTPGTIMIILIPIVDSPAELFVTSYVCILFITVANPIVQSYFRPEIKSTLLALCQRK
QANQFQSLELSKHSM

Aq1 >PAC:15710686 ID:710686 scaffold:Aqu1:Contig12965:9018:9935:1
gene:Aqu1.212158

MNNFTYNGDFNNVAVGAALAVEGVSGFLANTFVLSITLYQRKSWKQSSTIFFTSLILANFVMVLLHFPFAVTALAAGE
WIFGSTDEEKTGTCTFAALTFWYSSIVIILTLAAISFDRFLFIVKPHLHKQFMRPWVALTLTIAIWTLSAVLSSTPFY
GLGNFSFDELLGFCFPLWISVDFIVYSLVIILLLFSVIIVTSVWTLCFTHRFITQQSQLSNDDTYGSKKKWLLGIFGS
MLLVYGICFMPAVIAIILIPVIEVPIALVISSIICSLFITVANPIVQSCFRPEIKSAFLSLFKKPSPFVSV

→ Aq2.1 Gene: Aqu2.1.18586 Scaffold Contig12965: 9,018-9,935

MNNFTYNGDFNNVAVGAALAVEGVSGFLANTFVLSITLYQRKSWKQSSTIFFTSLILANFVMVLLHFPFAVTALAAGE
WIFGSTDEEKTGTCTFAALTFWYSSIVIILTLAAISFDRFLFIVKPHLHKQFMRPWVALTLTIAIWTLSAVLSSTPFY
GLGNFSFDELLGFCFPLWISVDFIVYSLVIILLLFSVIIVTSVWTLCFTHRFITQQSQLSNDDTYGSKKKWLLGIFGS
MLLVYGICFMPAVIAIILIPVIEVPIALVISSIICSLFITVANPIVQSCFRPEIKSAFLSLFKKPSPFVSV

Aq1 >PAC:15710687 ID:710687 scaffold:Aqu1:Contig12965:10196:11158:1
gene:Aqu1.212159

MESNFSFTGSFNRPVAITVLSITTLAALIANSVVLFITLYQRKSWKQSSTIFFTSLILSNFILNLLYLPFTIIALAVG
EWIFGSTDEEKRGTCAFAAFIVCYTVLVQSFTLAAISFDRFLFIVKPHLHKRFMRPWVALTLTIAIWILSALFGSTPF
YGLGKFAYLPNVGTCSPAFTEYFFVGYSAAIEVAVFLFIIITSVWTFCFTYKFIRDQSAIGEESVYLSRKKRLVGIFG
AMLLVYVICLLPIFTVAILFPVVDISSHVVASVVFVLHLFTVINPLVQSYFRPGFKDLILTVVMKW
PKRRLTNAMLRLNRMPNQSN

→ Aq2.1 Gene: Aqu2.1.18588 Scaffold Contig12965: 10,196-11,158

MESNFSFTGSFNRPVAITVLSITTLAALIANSVVLFITLYQRKSWKQSSTIFFTSLILSNFILNLLYLPFTIIALAVG
EWIFGSTDEEKRGTCAFAAFIVCYTVLVQSFTLAAISFDRFLFIVKPHLHKRFMRPWVALTLTIAIWILSALFGSTPF
YGLGKFAYLPNVGTCSPAFTEYFFVGYSAAIEVAVFLFIIITSVWTFCFTYKFIRDQSAIGEESVYLSRKKRLVGIFG
AMLLVYVICLLPIFTVAILFPVVDISSHVVASVVFVLHLFTVINPLVQSYFRPGFKDLILTVVMKW
PKRRLTNAMLRLNRMPNQSN

Aq1 >PAC:15710689 ID:710689 scaffold:Aqu1:Contig12965:15638:23368:1
gene:Aqu1.212161

MSNFTYNGDFSNVAAGAALAVEGVSGFLANTFVLSITLYQWKSWKQSSTIFFTSLILANFVMVLLHFPFAVIAIAAGE
WIFGSTDEEKTGTCTLAALTFWYSSLVLVITLAAISFDRFLFIVKPHLHKRFMRPWVALTLTIAIWILSAVLGSTPFY
GLGGFGFNTSIGMCLPFLIKDIILAFLILIISLLLIIVITSLWTFFFTWWFLHRRSMVVESNIYLSKKRGLFGIFGAM
LLVYGISKGPRIISLVLVQFEVPFSSTAEIAIYFVYQLSIIADPIVQSFFRPGFKQAMVSLFKKCKNNVAVGAALAVE
GVSGFIANTFVLSITLYQRKSWKQSSTIFFTSLILANFVMVVLHFPFTIIALAAGEWIFGSTDEEKRGTCTFAAFTFW
YSSLIIVMTIAAISFDRFFFIVKPHLHKRFMRPWVALTLTIAIWILSAVLSSTPFYGLGDFGFIASIGLCLSFLIKDG
SVILAFIILILSLLIIVITSLWTFFFTWWFLHRRSMVVESNIYSSKKRGLFGIFGAVLLVYGISKGPRIIALVLVQFE
VPFSSTALIMIYLVYQLSIIADPVVQSFFRPGFKQILGRPIAMYITVISLLQQQTIANAASSHNSALLVAEHHKRVVH
LACLEEAALIMNNFTYNGDFSNVAVGAALAVEGVSGFIANTFVLSITLYQWKSLKQSSTIFFTSLILANFVMVVLHFP
FAVTALAAGEWIFGSTDEEKIGTCTFAAFTFLYSSVVIILTLAAISFDRFFFIVKPLLHKQFMRPWVALTLTIVIWIA
AAVLSSTPFYGLGDFGFVASIGLCIPLLVKDGFVILAFTILILSLLIIIITSVWTFFFTWWFLRKRSMVVESNIYLSK
KRGLFGIFGAMLLVYGISKGPRIILLVLVQFEVPFSGTALIVIYFVYQLCIIADPIAQSFFRPGFKQAMLAWKKLLVM
NNFTYNGDFSNVAVGAALAVEGVSGFIANTFVLSVTLYQWKSLKQSSTIFFTSLILANLLMVLLHFPFTIIALAAGEW
IFGSTDEEKTGTCAFAAFTFWYSSLVLVMTVAAISFDRFLFIVKPHLHKRFMRPWVALTLTIAIWILSAVLSSTPLYG
LGGFGFNASIGMCLPFLIKDASVISAFLILILSLLLIIVITSLWTFFFTWWFLHKRSMVVESNIYSSKKRGLIGIFGA
MLLVYGISKGPRIISLVLVQFDVPFSSTAEIVIYFVFQLSIIADPVVQSFFRPGFKQVLVSLFKKCKK

→ Aq2.1 Gene: Aqu2.1.18590 Scaffold Contig12965: 15,638-26,285

MSNFTYNGDFSNVAAGAALAVEGVSGFLANTFVLSITLYQWKSWKQSSTIFFTSLILANFVMVLLHFPFAVIAIAAGE
WIFGSTDEEKTGTCTLAALTFWYSSLVLVITLAAISFDRFLFIVKPHLHKRFMRPWVALTLTIAIWILSAVLGSTPFY
GLGGFGFNTSIGMCLPFLIKDIILAFLILIISLLLIIVITSLWTFFFTWWFLHRRSMVVESNIYLSKKRGLFGIFGAM
LLVYGISKGPRIISLVLVQFEVPFSSTAEIAIYFVYQLSIIADPIVQSFFRPGFKQAMVSLFKKCKNNVAVGAALAVE
GVSGFIANTFVLSITLYQRKSWKQSSTIFFTSLILANFVMVVLHFPFTIIALAAGEWIFGSTDEEKRGTCTFAAFTFW
YSSLIIVMTIAAISFDRFFFIVKPHLHKRFMRPWVALTLTIAIWILSAVLSSTPFYGLGDFGFIASIGLCLSFLIKDG
SVILAFIILILSLLIIVITSLWTFFFTWWFLHRRSMVVESNIYSSKKRGLFGIFGAVLLVYGISKGPRIIALVLVQFE
VPFSSTALIMIYLVYQLSIIADPVVQSFFRPGFKQILGRPIAMYITVISLLQQQTIANAASSHNSALLVAEHHKRVVH
LACLEEAALIMNNFTYNGDFSNVAVGAALAVEGVSGFIANTFVLSITLYQWKSLKQSSTIFFTSLILANFVMVVLHFP
FAVTALAAGEWIFGSTDEEKIGTCTFAAFTFLYSSVVIILTLAAISFDRFFFIVKPLLHKQFMRPWVALTLTIVIWIA
AAVLSSTPFYGLGDFGFVASIGLCIPLLVKDGFVILAFTILILSLLIIIITSVWTFFFTWWFLRKRSMVVESNIYLSK
KRGLFGIFGAMLLVYGISKGPRIILLVLVQFEVPFSGTALIVIYFVYQLCIIADPIAQSFFRPGFKQAMLAWKKLLVM
NNFTYNGDFSNVAVGAALAVEGVSGFIANTFVLSVTLYQWKSLKQSSTIFFTSLILANLLMVLLHFPFTIIALAAGEW
IFGSTDEEKTGTCAFAAFTFWYSSLVLVMTVAAISFDRFLFIVKPHLHKRFMRPWVALTLTIAIWILSAVLSSTPLYG
LGGFGFNASIGMCLPFLIKDASVISAFLILILSLLLIIVITSLWTFFFTWWFLHKRSMVVESNIYSSKKRGLIGIFGA
MLLVYGISKGPRIISLVLVQFDVPFSSTAEIVIYFVFQLSIIADPVVQSFFRPGFKQVLVSLFKKCIEKLKFKPAYNP
YTEPSMEVFSYHSGLKKWVEIGNSGIFRPEMLLPMGLPKDVSVIAWGLSLERPTMIKYKLNNIRELVGHKTYIAQDIN
GPLLAAVFATEMVLALIANGVVLLITITQRNSSRNSWKQPSTVFFTSLILAHLVMNLLYLPLTVIAMASEEWIFGSTD
EEKRLTCTFAAFTVWYIVIVISMTLAAISFDRFLFIVKPHLHKRFMRPWVALTVAITIWLLAAVINSTPFIGVGEFGY
ATSYGTCVPFWEENIVYVFFMLAVFALITGVIAVTSLWTFCFTRKFLHDQSLVAGSESIYNSTRKKLFGIFGAMLIIY
TICLSPGFIIGSLSQLVSLPGSAYAFVIVCFVALTIGNPLVQSYFRLDVKSCLSCMRHKI
KEHHSRAGRYHHETESCRV

Aq1 >PAC:15710691 ID:710691 scaffold:Aqu1:Contig12965:26889:27827:1
gene:Aqu1.212163

MDEVNDTLYTFTGSVNTYVLAAVFVLEFIAGFIFNTIVIGITIYKGSWKKQGTIFFTSLILANLLLVILYIPFLVIGL
AARKWIFGNTVAEKLASCWFVGFALWYSFIIILMTLAAISLDRFLFVIKPHFHKWFMRPWVTLTLTIAIWILSAVLSS
TPFYGLGTYAYGRWYGSCVPLWVEIGFIIYSVTISLLVIAVIVITSVWVFCFTRIFIKVHPEVEVPADPDTVICLSKK
KRLFGIYGSMLLAYTVCFAPTLISSIISSSIDIPGEVFFLNVITFFLITVVSPLIQSYFRKDIKEV
IVLCYKKITKKQ

→ Aq2.1 Gene: Aqu2.1.18592 Scaffold Contig12965: 26,889-27,925

MDEVNDTLYTFTGSVNTYVLAAVFVLEFIAGFIFNTIVIGITIYKGSWKKQGTIFFTSLILANLLLVILYIPFLVIGL
AARKWIFGNTVAEKLASCWFVGFALWYSFIIILMTLAAISLDRFLFVIKPHFHKWFMRPWVTLTLTIAIWILSAVLSS
TPFYGLGTYAYGRWYGSCVPLWVEIGFIIYSVTISLLVIAVIVITSVWVFCFTRIFIKVHPEVEVPADPDTVICLSKK
KRLFGIYGSMLLAYTVCFAPTLISSIISSSIDIPGEVFFLNVITFFLITVVSPLIQSYFRKDIKEV
IVLCYKKITKKQ

Aq1 >PAC:15710692 ID:710692 scaffold:Aqu1:Contig12965:28337:29296:1
gene:Aqu1.212164

MEYHNFTATGDVNGPVLAAVLAVEAVVAFIANIIVLSITLYQRKSWKQSSTIFFTSLILSNLLAVVGYLPMNIVAIGA
EEWIFGSTFEERSITCLISALIIQLNIAVTTEILAAISFDRCLFVVKPHLHKRFMRPWVALTLTIAIWIFCILLLMLP
LLGFGKYSYEFSYGPCIPMLIKSPVYSFVHAALHIISFAVIIITSIWTCCFTQRFINDQSEIVGDNSVYASRKKKLFG
IFGSMFLAYSFCYGPSYITLMLYPVVEIDEIVYAADMVTFQFITVASPLIQAYFRPDIKNALIWFKKSIVSQIGKRLY
SPPQNEQ

→ Aq2.1 Gene: Aqu2.1.18593 Scaffold Contig12965: 28,337-29,382

MEYHNFTATGDVNGPVLAAVLAVEAVVAFIANIIVLSITLYQRKSWKQSSTIFFTSLILSNLLAVVGYLPMNIVAIGA
EEWIFGSTFEERSITCLISALIIQLNIAVTTEILAAISFDRCLFVVKPHLHKRFMRPWVALTLTIAIWIFCILLLMLP
LLGFGKYSYEFSYGPCIPMLIKSPVYSFVHAALHIISFAVIIITSIWTCCFTQRFINDQSEIVGDNSVYASRKKKLFG
IFGSMFLAYSFCYGPSYITLMLYPVVEIDEIVYAADMVTFQFITVASPLIQAYFRPDIKNALIWFK
KSIVSQIGKRLYSPPQNEQ

Aq1 >PAC:15710693 ID:710693 scaffold:Aqu1:Contig12965:30227:31309:1
gene:Aqu1.212165

MSIQSENNFTLNDDVNGPVLAAVFATEMILALIANGVVLLITITQRNSWKQSSTIFFTSLILAHLVLNLLYLPFTIIA
LAAGEWIFGSTDEEKRETCDFVAYTLWYSIPVITITLAAISFDRFLFIVKPHLHKQFMRPWVALTLTLAIWILSAVVT
FTPFIKGSGAAFIYEGSHGTCTIILLELEFAIVCFLGSLLVVSIIVVTSVWTFCFTRKFIHNQSEIAGDNVYASRKKR
LFGIFGAMLIVYGLCFTPGIINYAVVRVIFVDDRIYLAIVISFIFVTVLSPIVQSYFRPEIKNKIVFICRKILQKPPQ
RDRVNYPVSSNNNSSGSKTEINRIGTSTQLTMSLDNALNTEHSSTTVV

→ Aq2.1 Gene: Aqu2.1.18594 Scaffold Contig12965: 30,227-31,852

MSIQSENNFTLNDDVNGPVLAAVFATEMILALIANGVVLLITITQRNSWKQSSTIFFTSLILAHLVLNLLYLPFTIIA
LAAGEWIFGSTDEEKRETCDFVAYTLWYSIPVITITLAAISFDRFLFIVKPHLHKQFMRPWVALTLTLAIWILSAVVT
FTPFIKGSGAAFIYEGSHVWTFCFTRKFIHNQSEIAGDNVYASRKKRLFGIFGAMLIVYGLCFTPGIINYAVVRPPQR
DRVNYPVSSNNNSSGSKTEINRIGTSTQLTMSLDNALNTEHSSTTVNKENHFKIRVMTLAVPKLVKLKLVTVTVNKCK
VYFAIDVNLFPV

Aq1 >PAC:15710694 ID:710694 scaffold:Aqu1:Contig12965:44866:45948:1
gene:Aqu1.212166

MSISSENNFTINDDVNGPVLAAVFATEMILALIANGAVLLITITQRNSWKQSSTIFFTSLILAHLVLTLLYLPFNIIA
LAAGEWIFGSTDEEKRGFCSFISYTLWYSIPVITITLAAISFDRFLFIVKPHLHKRFMRPWVALTLTLAIWILSAVTT
FTPFIEGSGAVFMYEGSHGTCTVVILELHFAIFSFVGSLLVVGIITVTSVWTFCFARKFIHNQSEIAGDHVYASRKKR
LFGIFGAMLIVYGLCFTPGIINYVVVRAIVVADEIYLAVVISFIFVTVLSPIVQSYFRPEIKNKIVCICRKILQKPPQ
RDSVDYLGNSNNDSSGSKTETNQIRTSAQLTMSLDCVLNTEHLSTTVV

→ Aq2.1 Gene: Aqu2.1.18595 Scaffold Contig12965: 44,866-45,948

MSISSENNFTINDDVNGPVLAAVFATEMILALIANGAVLLITITQRNSWKQSSTIFFTSLILAHLVLTLLYLPFNIIA
LAAGEWIFGSTDEEKRGFCSFISYTLWYSIPVITITLAAISFDRFLFIVKPHLHKRFMRPWVALTLTLAIWILSAVTT
FTPFIEGSGAVFMYEGSHGTCTVVILELHFAIFSFVGSLLVVGIITVTSVWTFCFARKFIHNQSEIAGDHVYASRKKR
LFGIFGAMLIVYGLCFTPGIINYVVVRAIVVADEIYLAVVISFIFVTVLSPIVQSYFRPEIKNKIVCICRKILQKPPQ
RDSVDYLGNSNNDSSGSKTETNQIRTSAQLTMSLDCVLNTEHLSTTVV

Aq1 >PAC:15710695 ID:710695 scaffold:Aqu1:Contig12965:48640:49623:1
gene:Aqu1.212167

MEGNFTFTGDFNTPAVAAVLTVEMILALIANGVVLSITLYQRKSWKQSSTIFFTSLILAHLVLNLLYLPFTVIGFAAG
EWIFGSTDQVKRGFCKFNAYILWYSILNIIMMLAAISVDRFLFIVKPHLHKRFMSQPKVALTLTIAIWLLAAVLSSTP
FFGLGEFGYETWFGGCLPLWTTIEFTVYTIIVCLVIIFIISLTSIWTFCFARRFLTDQSRLTTENNVYYSSKKQRLIG
IFGAMSLVYLLCFSPGIISAFLTPLTLVPYEVIVLVIVTIELLTIAGPLVQSYFRPDINKVLVSLYTKMRRLFRRNMQ
SVCSNRELDTKISYV

→ Aq2.1 Gene: Aqu2.1.18598 Scaffold Contig12965: 48,380-49,718

MEGNFTFTGDFNTPAVAAVLTVEMILALIANGVVLSITLYQRKSWKQSSTIFFTSLILAHLVLNLLYLPFTVIGFAAG
EWIFGSTDQVKRGFCKFNAYILWYSILNIIMMLAAISVDRFLFIVKPHLHKRFMSQPKVALTLTIAIWLLAAVLSSTP
FFGLGEFGYETWFGGCLPLWTTIEFTVYTIIVCLVIIFIISLTSIWTFCFARRFLTDQSRLTTENNVYYSSKKQRLIG
IFGAMSLVYLLCFSPGIISAFLTPLTLVPYEVIVLVIVTIELLTIAGPLVQSYFRPDINKVLVSLYTKMRRLFRRNMQ
SVCTNVEFVL

Aq1 >PAC:15710773 ID:710773 scaffold:Aqu1:Contig12972:28306:29304:-1
gene:Aqu1.212245

MERNYTFTGDFSPEAVAGVLSIEMILALIANGVVLVITIYQRKSWKQSSTIFFTSLILAHLVLTLYLPFFIAALAAGE
WIIGSTDGEKKATCDFNGFIIIHSGYVMLMTLSLISIDRFLFIVKPHLHKRFMSPRVALVLVIIVWIVTALFFSLGFI
DGSGVVFRYIDNVGGCYVYTTSPVTSIIRYLFASLIFCTIVVTSIWTFCFTRMFMNNQSMIVGESVYASKKKRLFGIF
GSMMVVYCIFFTPAIFFSALLAIVDSPGVLSMTALIFFFLVVVLNPVVQAYFRPEINSVIVNIVCCKILKKKPNPATT
LSTFNSLSATLEFSVSSLDV

→ Aq2.1 Gene: Aqu2.1.18708 Scaffold Contig12972: 28,306-29,304

MERNYTFTGDFSPEAVAGVLSIEMILALIANGVVLVITIYQRKSWKQSSTIFFTSLILAHLVLTLYLPFFIAALAAGE
WIIGSTDGEKKATCDFNGFIIIHSGYVMLMTLSLISIDRFLFIVKPHLHKRFMSPRVALVLVIIVWIVTALFFSLGFI
DGSGVVFRYIDNVGGCYVYTTSPVTSIIRYLFASLIFCTIVVTSIWTFCFTRMFMNNQSMIVGESVYASKKKRLFGIF
GSMMVVYCIFFTPAIFFSALLAIVDSPGVLSMTALIFFFLVVVLNPVVQAYFRPEINSVIVNIVCCKILKKKPNPATT
LSTFNSLSATLEFSVSSLDV

Aq1 >PAC:15710774 ID:710774 scaffold:Aqu1:Contig12972:34323:35321:-1
gene:Aqu1.212246

MERNYTFTGDFSPEAVAGVLSIEMILALIANGVVLVITIYQRKSWKQSSTIFFTSLILAHLALTLYLPFTIAALAAHE
WVVGSTDGEKQGTCSFTAFVILLSVYVMLMTLSLISIDRFLSIVKPHLHKRFMSPRVALVLVIIVWSFDLLFFSTGFI
EDSGVEFRYIDYLGICYAFTTSPVMAIFRFFFASLIFIIIIVTSVWTFCFTRKFINNQSMIVGESIYASKKKRLFGIF
GSMLLVYGIFFTPSIFFSALLAIVDSPGVLTISALMFFFLVVVLSPVVQAYFRPEINSVIVNIVCRKILKKKHNPAAT
SSTCNSSSAAVELRVSSLDV

→ Aq2.1 Gene: Aqu2.1.18710 Scaffold Contig12972: 34,323-35,321

MERNYTFTGDFSPEAVAGVLSIEMILALIANGVVLVITIYQRKSWKQSSTIFFTSLILAHLALTLYLPFTIAALAAHE
WVVGSTDGEKQGTCSFTAFVILLSVYVMLMTLSLISIDRFLSIVKPHLHKRFMSPRVALVLVIIVWSFDLLFFSTGFI
EDSGVEFRYIDYLGICYAFTTSPVMAIFRFFFASLIFIIIIVTSVWTFCFTRKFINNQSMIVGESIYASKKKRLFGIF
GSMLLVYGIFFTPSIFFSALLAIVDSPGVLTISALMFFFLVVVLSPVVQAYFRPEINSVIVNIVCRKILKKKHNPAAT
SSTCNSSSAAVELRVSSLDV

Aq1 >PAC:15710775 ID:710775 scaffold:Aqu1:Contig12972:36940:38446:-1
gene:Aqu1.212247

METNYTFTGDFSPEAVAGVLSIEMILALIANGVVLVITIYQRKSWKQSSTIFFTSLILAHLVLTLFLPFFIAALASGE
WIIGDTDEEKKATCGFTAFVMIYCGYVMLMTLSLISIDRFLFIVKPHLHKRFMSPRVALVLVIIVWIVTAVLNSSGFI
NGSGVFGRFVSLSMIVGESVYASKKKRLFGIFGSMLLVYGICVSPIIFFSALLAIIDSPGVLGITAVIFFFLVVVLSP
VVQAYFRPEINTVIANVIVNIACHKMSKKKPNSATTSSSSSAAVELRVSSLDFSLVIERMERNYTFTGDFSPEAVAGV
LSIEMILALIANGVVLVITIYQRKSWK

→ Aq2.1 Gene: Aqu2.1.18709 Scaffold Contig12972: 31,434-38,446

METNYTFTGDFSPEAVAGVLSIEMILALIANGVVLVITIYQRKSWKQSSTIFFTSLILAHLVLTLFLPFFIAALASGE
WIIGDTDEEKKATCGFTAFVMIYCGYVMLMTLSLISIDRFLFIVKPHLHKRFMSPRVALVLVIIVWIVTAVLNSSGFI
NGSGVFGRFVSLSMIVGESIYASKKKRLFGIFGSMLLVYGIFFTPSIFFSALLAIVDSPGVLTISALMFFFLVVVLSP
VVQAYFRPEINSVIVNIVCRKILKKKHNPAATSSTCNSSSAAVELRVSSLDYYKFSHNVSIIALLFSLVIERMERNYT
FTGDFSPEAVAGVLSIEMILALIANGVILVITIYQRKSWKQSSTIFFTSLILAHLVLTLYLPFTITSLAAGEWIIGNT
DEEKKPHLHKRFMSPRVALVLVIIVWIVPAAFFSSGFINGSGAEFRYIDYLGVCSAFTTSPVMAIFRFFFALLMRLIC
NAATYIIRPFKTGPRGTGLPGSTVALLFSLVKERMERNYTFTGDFSPEAVAGILCLEMILSFLANGVVLVITIYKRKS
WKQSSTIFFTSLILAHLVLTLYLPFTFAALAAREWVVGDTDEEKQGTCGFTVYIILFSCYVMLMTLSLVSIDRFLSIV
KPHLHKRFMSPRVALVLVIIVWMINAVLFSSGFMNGSGVVYRYIDYLGICYAFTTSPVMAIFRFLLASSLLCIIVVTS
IWTFCFTRKFINNQSMIVGESVYASKKKKLFGIFGSMMLVYCIFFTPSIFFGTLLAIVDSPGVLTISALMFCFLVVVL
SPVVQAYFRPEINSVIVNIVCRKILKKKPHPVTTSSTCNSSSAAVELRVSSLDV

Aq1 >PAC:15710777 ID:710777 scaffold:Aqu1:Contig12972:48557:49546:-1
gene:Aqu1.212249

MERNYTFNGSFSPEAVAGVLSIEMVLALIANGVVLVVTIYQRKSWKQSSTIFFTSLILAHLVLTLSLPFSIAALAARE
WVVGDTDEEKQGTCGFTAFMILFSVYVMLMTLSLISIDRFLFIVKPHLHKRFMSPRVALVLVIIVWIVPAAFFSSGFI
NGSGAEFRYIDYLGVCSAFTTSPVMAIFRFFFASLMFGRFASLSMIVEESVYASKKKRLFGSMLLVYGICFTPSIFFS
TLVAIVDSPGALTLTALMLFFFAVIPSPVIQAYFRPEINSVIVDIVCRKMLKKKPNKLATSSTCDSSSAAVELRVSSL
DP

→ Aq2.1 Gene: Aqu2.1.18712 Scaffold Contig12972: 43,224-51,853

MVLALIANAAREWVVGDTDEEKQGTCGFTAFMILFSVYVMLMTLSLISIDRFLFIVKPHLHKRFMSPRVALVLVIIVW
IVPAAFFSSGFINGSGAEFRYIDYLGVCSAFTTSPVMAIFRFFFASLMVIIIIITSVWTFCFTLEESVYASKKKRLFG
SMLLVYGICFTPSIFFSTLVAIVDSPGALTLTALMLFFFAVIPSPVIQAYFRPEINSVIVDIVCRKMLKKKPNKLATS
STCDSSSAAVELRECLCFKEEEVVWYIWVNATGIWDMFHSCYILWYIISHY

Aq1 >PAC:15710836 ID:710836 scaffold:Aqu1:Contig12980:17612:18616:-1
gene:Aqu1.212308

MESNYTYTGDFSPEAVAAVLSIEMILALIANGVVLVITIYQRKSLKQPSTIFFTSLILGHLVMILYLPFSITALAAGE
WIIGSTDEERQGSCDFSAFIILYCVYVMLMTLSLISIDRFLFIVKPHLHKRFISPRVALVLVIIVWVFNAAFFSSGFI
DGSGVVYQYIPDLGVCYAYTISTIAAVIRFLLTLVLLCIIVVTSVWTFCFTRKFINDQSEIVGDCVYASRKKRLFGIF
GSMLLIYGVCFIPSAVLSSFLAIADPPGALVVSALILFFLAIVLSPVVQSYFRPEINSVIVNIICH
KIMKKPHTTTSSMCNNSSANRGTNIDSLDINLHA

→ Aq2.1 Gene: Aqu2.1.18789 Scaffold Contig12980: 17,612-18,616

MESNYTYTGDFSPEAVAAVLSIEMILALIANGVVLVITIYQRKSLKQPSTIFFTSLILGHLVMILYLPFSITALAAGE
WIIGSTDEERQGSCDFSAFIILYCVYVMLMTLSLISIDRFLFIVKPHLHKRFISPRVALVLVIIVWVFNAAFFSSGFI
DGSGVVYQYIPDLGVCYAYTISTIAAVIRFLLTLVLLCIIVVTSVWTFCFTRKFINDQSEIVGDCVYASRKKRLFGIF
GSMLLIYGVCFIPSAVLSSFLAIADPPGALVVSALILFFLAIVLSPVVQSYFRPEINSVIVNIICH
KIMKKPHTTTSSMCNNSSANRGTNIDSLDINLHA

Aq1 >PAC:15710930 ID:710930 scaffold:Aqu1:Contig12992:13727:14362:1
gene:Aqu1.212402

MNNSSTTSTREELHAAVIAAVLTVEMILAVIANGVVLSITLYERKSWKQSSTIFFTSLILAHLVLILLYLPFTIIALA
AGKWIFGSTDEEKTTTCTFVFYLYWYIIAVISMTLAAISFDRFLFIVKPHLHKRFMRPWVAQTLTIAIWLLSAIXXXX
XXXXXXXXXXXFFVFIAVANPVVQSYFRPDIKTFCFSHCPLLSTCLCCSCLHNVF

→ Aq2.1 Gene: Aqu2.1.18925 Scaffold Contig12992: 13,727-14,362

MNNSSTTSTREELHAAVIAAVLTVEMILAVIANGVVLSITLYERKSWKQSSTIFFTSLILAHLVLILLYLPFTIIALA

AGKWIFGSTDEEKTTTCTFVFYLYWYIIAVISMTLAAISFDRFLFIVKPHLHKRFMRPWVAQTLTIAIWLLSAIXXXX
XXXXXXXXXXXFFVFIAVANPVVQSYFRPDIKTFCFSHCPLLSTCLCCSCLHNVF

Aq1 >PAC:15710932 ID:710932 scaffold:Aqu1:Contig12992:24170:25111:1
gene:Aqu1.212404

MEGNFTFTGEFSGPAVAAVLTVEMILALIANGVVLSITLYQRKSWKQSSTIFFTSLILAHLVSNLLNLPFTIIALAAG
EWIFGSTDEEKTGTCDFAAWMLWFGASCMSMTLAALSFDRFLFIVKPHLHKWFMRPCVALTLTIAIWILSAVLCALPF
IDIGNYSYDNDIGICTIVGVDIAFFILILVIIFLIVGTIFVTSLWTFCFTRSYFKAQSVIAGESVYASKKKRLFGVFG
SMLLIYGISHLLVALWVLLQIFIFLPYEFFVIVYIVYFFVTIASPIIQSYFRPEIKSVLVSRCPLLFTCVCCSYVHAV
C

→ Aq2.1 Gene: Aqu2.1.18926 Scaffold Contig12992: 24,170-25,111

MEGNFTFTGEFSGPAVAAVLTVEMILALIANGVVLSITLYQRKSWKQSSTIFFTSLILAHLVSNLLNLPFTIIALAAG
EWIFGSTDEEKTGTCDFAAWMLWFGASCMSMTLAALSFDRFLFIVKPHLHKWFMRPCVALTLTIAIWILSAVLCALPF
IDIGNYSYDNDIGICTIVGVDIAFFILILVIIFLIVGTIFVTSLWTFCFTRSYFKAQSVIAGESVYASKKKRLFGVFG
SMLLIYGISHLLVALWVLLQIFIFLPYEFFVIVYIVYFFVTIASPIIQSYFRPEIKSVLVSRCPLLFTCVCCSYVHAV
C

Aq1 >PAC:15710934 ID:710934 scaffold:Aqu1:Contig12992:27942:28865:1
gene:Aqu1.212406

MEGNFTFTGEFSGPAVAAVLTVEMILALIANGVVLSITLYQRKSWKQPSTIFFTSLILAHLVLNLLYLPFSIIAFAAG
EWIFGSTDEEKKTTCTFTSFLYSYTMRIIFMTLAAISFDRFLFIVKPHLHKRFMRPWVALTLTIAIWILAAVLSSVPF
YGLNEYVYDDQLLLCYQRPNSTISLIIFNVLFPCVTTAIIIVTSLWTFCFARSYFKAQSLIAGESVYASKKKRLFGVF
GSMLLIYGMGIVPGLVLLSLQHFVDVPPELFISFVIFLFFITIASSIIQSYFRPEIKSVLASRCPLLFYCACC

→ Aq2.1 Gene: Aqu2.1.18928 Scaffold Contig12992: 27,942-28,865

MEGNFTFTGEFSGPAVAAVLTVEMILALIANGVVLSITLYQRKSWKQPSTIFFTSLILAHLVLNLLYLPFSIIAFAAG
EWIFGSTDEEKKTTCTFTSFLYSYTMRIIFMTLAAISFDRFLFIVKPHLHKRFMRPWVALTLTIAIWILAAVLSSVPF
YGLNEYVYDDQLLLCYQRPNSTISLIIFNVLFPCVTTAIIIVTSLWTFCFARSYFKAQSLIAGESVYASKKKRLFGVF
GSMLLIYGMGIVPGLVLLSLQHFVDVPPELFISFVIFLFFITIASSIIQSYFRPEIKSVLASRCPLLFYCACC

Aq1 >PAC:15710937 ID:710937 scaffold:Aqu1:Contig12992:44064:45005:1
gene:Aqu1.212409

MEGNFTFTGEFSGPAVAAVLTVEMILALIANGVVLSITLYQRKSLKQSSTIFFTSLILAHLVSNLLNLPFAIIALAAG
EWIFGSTDEEKRGTCIFAAFILWFGASFISMTLAAISFDRFLFIIKPHLHKRFMRPWVALTLTIAIWILSTVLCALPF
IDIGHYSYHDDLGICTIDGVDIAFFVVILVSIFLIVGTIFVTSLWTFCFTRSYFKDQSVIAGESVYAFKKKRLIGVFG
SMLLIYGISHLLVALWVLLQIFILLPSEFSVTSYIVYFFVTIASPIIQSYFRPEIKSVLVSRCPLLFTCVCCSCVHAV
C

→ Aq2.1 Gene: Aqu2.1.18931 Scaffold Contig12992: 44,064-45,005

MEGNFTFTGEFSGPAVAAVLTVEMILALIANGVVLSITLYQRKSLKQSSTIFFTSLILAHLVSNLLNLPFAIIALAAG
EWIFGSTDEEKRGTCIFAAFILWFGASFISMTLAAISFDRFLFIIKPHLHKRFMRPWVALTLTIAIWILSTVLCALPF
IDIGHYSYHDDLGICTIDGVDIAFFVVILVSIFLIVGTIFVTSLWTFCFTRSYFKDQSVIAGESVYAFKKKRLIGVFG
SMLLIYGISHLLVALWVLLQIFILLPSEFSVTSYIVYFFVTIASPIIQSYFRPEIKSVLVSRCPLLFTCVCCSCVHAV
C

Aq1 >PAC:15710938 ID:710938 scaffold:Aqu1:Contig12992:45265:46197:1
gene:Aqu1.212410

MEGNFTFTGQFSGPAVAAVLTFEMILALIANGLVLSITLYQRKSLKQSSTIFFTSLILAHLVLNLLYLPFTIIALAAG
EWIFGNTDEEKIATCTFTAFLYSYTIRIIFMTLAAISFDRFLFIVKPYLHKWFMRPWVALTLTIAIWILSAVLSSAPF
YGLNEYVYDDQLLLCYQRPNSTISFIFYVLFPCVTTAIIIVTSLWTFCFAGSFFKDQSVIAGESVYASKKKRLFGVFG

SMLFIYGICIVPGLVLRSLQEFIDVPPKLSISSMTCFLFITIASPIIQSYFRPEIKSVLASRCPLLFTCACCKFIH

→ Aq2.1 Gene: Aqu2.1.18932 Scaffold Contig12992: 45,265-46,197

MEGNFTFTGQFSGPAVAAVLTFEMILALIANGLVLSITLYQRKSLKQSSTIFFTSLILAHLVLNLLYLPFTIIALAAG
EWIFGNTDEEKIATCTFTAFLYSYTIRIIFMTLAAISFDRFLFIVKPYLHKWFMRPWVALTLTIAIWILSAVLSSAPF
YGLNEYVYDDQLLLCYQRPNSTISFIFYVLFPCVTTAIIIVTSLWTFCFAGSFFKDQSVIAGESVYASKKKRLFGVFG
SMLFIYGICIVPGLVLRSLQEFIDVPPKLSISSMTCFLFITIASPIIQSYFRPEIKSVLASRCPLLFTCACCKFIH

Aq1 >PAC:15710939 ID:710939 scaffold:Aqu1:Contig12992:46555:47452:1
gene:Aqu1.212411

MAKNFTFTGEFSGPAVAAVLTVEMILALIANGVVLSITLCQRKSLKQPSTIFFTSLILSHLVFNLLYLPFTIIALAAG
EWIFGSTDEEKTATCTFTAFIYSYTIPVIFMTLAAISFDRFLFIVKPHLHKRFMRPWVALTLTIAIWILSAVLSSTPF
YGLNEYVYDDHQSYYPVIL

→ Aq2.1 Gene: Aqu2.1.18933 Scaffold Contig12992: 46,270-46,786

MAKNFTFTGEFSGPAVAAVLTVEMILALIANGVVLSITLCQRKSLKQPSTIFFTSLILSHLVFNLLYLPFTIIALAA

Aq1 >PAC:15710940 ID:710940 scaffold:Aqu1:Contig12992:51121:52032:1
gene:Aqu1.212412

MESNFTFTGEFSGPAVAAVLTVEMILALIANGVVLSITLYQRKSWKQSSTIFFTSLILAHLVLNLLYLPFTIIALAAG
EWIFGSTDEQKTGTCYFNLFIFWWMVVTIFLMLTTISFDRFLFIVKPHLHKRFMRPWVALTLTIIVWLLAAVSNIIYL
LTFHSDIDPDLCYLLPINVNRSSVFAIVSTVIVLIIMFVTSLWTFCFTKSYFKAQSVIAGESVYASKKKRLFGVFGSM
LIIYGICFVPAVVYLIAFLFVDVPVWYSFTAFICYLFITVANPVIQSYFRPEIKSFFVSHSLHHFHICC

→ Aq2.1 Gene: Aqu2.1.18936 Scaffold Contig12992: 51,524-52,298

MFVTSLWTFCFTKSYFKAQSVIAGESVYASKKKRLFGVFGSMLIIYGICFVPAVVYLIAFLFVDVPVWYSFTAFICYL
FITVANPVIQSYFRPEIKSFFVSHSLHHFHICC

Aq1 >PAC:15710941 ID:710941 scaffold:Aqu1:Contig12992:54553:55494:1
gene:Aqu1.212413

MESNFTFTGDFSGPAVAAVFTVEMILGLITNGTVLFITLYQRKSWKQSSTIFFTSLILSHIVINLYLSTTIVALAAGG
WIFGSTDEEKTWTCIFAAWLLWSALAVLSYTLVALSFDRFLFIVKPLLHKRFMTPRVALTITIVIWIAAAVLNLWPVL
HNFEHYGYLNDLGSCSIIGVDLAAFVVFLISNFIIFGSIAVTSLWTFCFTRSYFKAQSVIAGESVYASKKKRLIGIFG
CMLIVYGLFYLQSVVSFLLQLFIVVPNQFFLALSVTFSLVTIASPIIQSYFRPEIKSVLVSRCPLLFTCVCCSCVHSI
C

→ Aq2.1 Gene: Aqu2.1.18940 Scaffold Contig12992: 54,989-55,494

AAVLNLWPVLHNFEHYGYLNDLGSCSIIGVDLAAFVVFLISNFIIFGSIAVTSLWTFCFTRSYFKAQSVIAGESVYAS
KKKRLIGIFGCMLIVYGLFYLQSVVSFLLQLFIVVPNQFFLALSVTFSLVTIASPIIQSYFRPEIKSVLVSRCPLLFT
CVCCSCVHSIC

Aq1 >PAC:15710942 ID:710942 scaffold:Aqu1:Contig12992:57178:58116:1
gene:Aqu1.212414

MESNFTFTGEFSGPAVAAVLTVEMILGLITNGTVLFITLYQRKSWKQSSTIFFTSLILSHIVLNLQLSFSIIALAAGG
WIFGSTDEEKTWTCIFAAWLLWSALAVLSFTLVALSFDRFLFIVKPHLHKRFMIPRVSLTITIVIWIAAVILNLWPIL
HNFEHYGYLNDLGGCSIVGVDIAAFVVFLVSNFLVFGSITITSLWTFCFTRSYFKAQSVIAGESVYASKKKRLIGIFG
CMLIVYGLFYLQSVVSFFLQLFIVVPNEFFLALFLTFSFVTIASPIIQSYFRPEIKSVLVSCCPLLFTCVCCSCVHAV

→ Aq2.1 Gene: Aqu2.1.18941 Scaffold Contig12992: 57,178-58,116

MESNFTFTGEFSGPAVAAVLTVEMILGLITNGTVLFITLYQRKSWKQSSTIFFTSLILSHIVLNLQLSFSIIALAAGG
WIFGSTDEEKTWTCIFAAWLLWSALAVLSFTLVALSFDRFLFIVKPHLHKRFMIPRVSLTITIVIWIAAVILNLWPIL
HNFEHYGYLNDLGGCSIVGVDIAAFVVFLVSNFLVFGSITITSLWTFCFTRSYFKAQSVIAGESVYASKKKRLIGIFG
CMLIVYGLFYLQSVVSFFLQLFIVVPNEFFLALFLTFSFVTIASPIIQSYFRPEIKSVLVSCCPLLFTCVCCSCVHAV

Aq1 >PAC:15710943 ID:710943 scaffold:Aqu1:Contig12992:60240:61184:1
gene:Aqu1.212415

MESNFTFTGKLSGPAVAAVHTVALILALIANGIVLSITLYQRKSWKQSSTIFFTSLILAHLVLNLLYLPFTIIALAAG
EWIFGSTDEEKTGTCAFAAFILWFGACVISMTLAAISFDRFLFIVKPHLHKQFMRPWVALTLTIAIWILSASVFGSLP
FLDIGHYSYDDELGFCILIDVKIASFVVILVIVLLVVGTIFVTSLWTFCFTKSYFKAQSVIAGESVYASKKKRLFGVF
GSMLLIYGTAYLFVALGILLQIFISPPYELYISYYIVFFFVTIASPIIQSYFRPEIKSVLVSRCPLLFTCVCCSCVHA
VC

→ Aq2.1 Gene: Aqu2.1.18942 Scaffold Contig12992: 60,240-61,184

MESNFTFTGKLSGPAVAAVHTVALILALIANGIVLSITLYQRKSWKQSSTIFFTSLILAHLVLNLLYLPFTIIALAAG
EWIFGSTDEEKTGTCAFAAFILWFGACVISMTLAAISFDRFLFIVKPHLHKQFMRPWVALTLTIAIWILSASVFGSLP
FLDIGHYSYDDELGFCILIDVKIASFVVILVIVLLVVGTIFVTSLWTFCFTKSYFKAQSVIAGESVYASKKKRLFGVF
GSMLLIYGTAYLFVALGILLQIFISPPYELYISYYIVFFFVTIASPIIQSYFRPEIKSVLVSRCPLLFTCVCCSCVHA
VC

Aq1 >PAC:15711746 ID:711746 scaffold:Aqu1:Contig13069:63737:64684:1
gene:Aqu1.213218

MDIHGLNYTLGEDVNGPLLAAALAVEMIAALIVNTFVLVATFSQCKSLKLPSTILFTSLIMIHYVMALIYIPSWLISA
AYGEWIFGNTMQVKEATCNFAGFILNYNITFINATLAAISVDRWLFIVKPIFYKQYMKAKVALIVVASTWITSTLLNV
PPFFEMGKYVFSPFGSCEVKFENEASFSFLLLVVFFTEASIIIVSSIWTCCFTRRFIREHAQLADESAYVSKNRRIIG
IFGAMLVAYGVCYTPAVIVIVTSLFHDVPASYIAASLVFLLTVIIINPIIQSIFRPDVKKVIVKLCTICRKHQYSPEE
PQP

→ Aq2.1 Gene: Aqu2.1.20368 Scaffold Contig13069: 63,528-77,525

MDIHGLNYTLGEDVNGPLLAAALAVEMIAALIVNTFVLVATFSQCKSLKLPSTILFTSLIMIHYVMALIYIPSWLISA
AYGEWIFGNTMQVKEATCNFAGFILNYNITFINATLAAISVDRWLFIVKPIFYKQYMKAKVALIVVASTWITSTLLNV
PPFFEMGKYVFSPFGSCEVKFENEASFSFLLLVVFFTEASIIIVSSIWTCCFTRRFIREHAQLADESAYVSKNRRIIG
IFGAMLVAYGEEYQLQAVEESRSDEPGAVVVSEQSPVGLTISPSSSSVLPELEAVHGIIDAVTLKRIMKKRPNVKIIP
LLICFAGLPESAKKEALQEIFKTPITAGFSSHHILATNRSNYEIACVYSTSMLYNFGVQSCQFARSSEEILYKVPTEQ
PLRIYKDALLNKHMKELLAHLHQYSSRFCDKQNLDFVFTITRGATLINIWDVTLNTNVFYFLRAFSGHLINSRMWLFA
DLDKVSDNSNKKANEKEGKLQIWQKPLDYLLRCMRLCKGVTTHKRPCTVFASHSGEDSDKEIETKMKIFEEDLQRVAR
QLNVTEFKDQTLPFNKKVQGNYFLRFLKNFLGNVYRRTHVPVTWLFFRGALEHKEGMFMRKAELRQLAEECDMNDEDF
KEFCELFTSFGSIFDLSLVDDTSDIIIIKPNEFLSNLNKSFDNPPDSKVYTENGIITETTAREMFGANQGETFMSVLA
LVGMVAVVPGGKYAQDETHEVCYYMPCARKHKQERFIDKEAVRLLRNNRRPINFVNFEVAFTNCMLKHSFAQLQSSTD
ENRTIIKCTDNNSIITMTFRGDETEIKVMPPSKKHTLYVVQAFKEIAEIIDKKKGRGRFSYAFAIMCSKNEKEYHRLP
HDVKLCDECKSKAEYSDWIEALTEEPIPEKFKFVTDIEFDDVIFVTKELVACCDQEMLTDLFKKCFDADYKESLPPWL
NVLNQLTNWITQDLSNVPNSSATKAELAAKLNEWSSTKDGKIKALINNNGLLSFYAAHNEYTSDPFPLPQTLIAPFWA
DVDTRGGAGTVYCRETAASAIVSKVAQDVRLAFPNQPPFTAKSVVIVTWYIVGYYEENDNKLNTFQCVLATDGGRSYV
LFLYLDDGINWVTGDASGGSNGIGGTEAYVGFNFGGQNATYFAVQGSRTLAVIDIETTSNVEVPGLWIFQVNEVQIVT
STIGSCGTPDQLYANATILSYNTTTVNSTATYSCQNGYNLVGISVRTCLSSGNWSGSPPYCQIVNCTELMVDSSGALS
VSYSSNSYSYNSKATYSCQNGYILVGAAIRTCQSTGSWSGNAPYCQIVNCTELMVELSEGLSVSYSSNSYSYNSTITY
SCQNGYNLVGAAIRTCQSTGSWSGNEPYCQIVNCSELMVESDAVGGLSISYSSNSRSYNSTATYSCEDGYSLTGVSVR
TCLSDGNWSGDPPYCQSINCSELMVESNAYGGLSVSLSSTNVNSTATYSCTYEGYKLVGNGVRICQANGRWIAVFTNK
LGPFSDNPFYISTHLTAPTRPVFDLKINSSSPTSFMISWTPPMLDYLNGPNITYVILVTSANSSFNYTSNTNYATIVR
LQPFTTYWCRVAVRNNAGIGPYSLSIEIKTLEDVPSSAPVNVIFLFINARTLFIQWGLIPLSNRNVIVGEKTYIYKTS
DRNITIENLQPSYTYNISVAAFTNGRGPFSEPISITMPDDVPSGRPQSLNLASAGATSLSISWNPPDEEDRNGNINEY
IVNVTNTDTLVTTQFTTANNDFSIVDLNPDTTYSLSVAARNANGTGPFTQSLLTKTGQSAPSQPRHLKAIAISSSSIL
LAWESPSNPNGDITEYYINITDESTSTVVMQETSNITYVIITDLSPNNTYRCHVAAATTYIGQVSEYVFVNTGEDVPT
AAPLSVLPIAIDSTIISISWSPPNINTTNGVIRHYKVSIINMATGSEQTLTTANSHLTILMLTPFTTYSCQVMHQKTL
MPPPMDPTLILKWSPPNTPNGIIRYYQITLLDQTNSTVFHFNTTEFEFTVNELLSFTRYTCYVSAFTIAAGPAAVLTI
MTQESAPSNPPTSIMLKSVNYTSINVSWSPPAVPNGLIHLYIIRISNGLLVIDYNSTEEYMLITGLMPNQNYTVSLAA

VTIDVGPFSTNLVITLQEKVATDPPTGLCNELNGSNLTLHWLPPPDGSVDGIISGYRINCTGENEHFISKTVSMTRAS
VDLGKDTYYTCMVCILTSAGCGPSGVNYINTYSGFPVGPPQSLSVNATDLSILIDWEAPFDPFNIITSYAITYQLLDT
PVSLAVPRPVATVSGITDTMYLLELVLSASSYNITVYANTSNGTGIESEPIAITTQKSGSSTLLNFMCSAASSTDIDC
SWKNLSNDDYQLHYSILPSFNYYTNNEGTINEGRIHDLKPYVGYVISHQLSNTNLMSTVVLTGPEIPTASVEDISFNV
LSPDSFTLSWKLPPKNDKRSAGNNITSLRPKDVEITVAPHANNPDPSLAMEPFAREEVIVSNLEPDFEYSLTISIDNA
AGRGPSSSPLNISMPEAAPSGPPLNLVLSNVQQTSFTATWLPPNLIERNGRIISYMVQVTRVNLLQTRSFETQDTRIF
LQGYYPYEHIMIKVAARTSVGLGPYSSVLAFNTNENVPSVPVSFSGTVIGSDQVILSWDMPQTTDGVISQYQVIYSGY
TIASNIPVIGPIQINVPVNRLQLFRLRNGLVYHFSVRAKNTAGYGPNATTIIVLHDSIIGELGGATRDVSGVDGRMIA
VSVLAGLFGLLLCLFLSIGLICCLCARMRQKKREK

Aq1 >PAC:15712451 ID:712451 scaffold:Aqu1:Contig13121:67635:68712:1
gene:Aqu1.213923

MDDINNNFTLSQDVNGPLLAAVIGMEMLAGLITNSFVLILTACHLKNWKQPTTVFLSNMLANNLVVILFTMPLSIITT
ASGEWIFGSTVSQKESVCYFAAFIFIFSILTATESLVLLSFDRFFFIVKALHYKKYMTVNRAFIIVAVSWLLAAFLSM
LPFFGFGAFEFASSYGMCVPGWTGQAGYAIFSFIVISIFIGSITVTSLWTMCFTRKYLKNAATNISTAASPAAYAVAF
VVLLLLTVLIPLVQSFFRRDIRDAIVKGHRTFFRSIKQEKTYHQSSSTQTVKPSNATTSSYTI

→ Aq2.1 Gene: Aqu2.1.21488 Scaffold Contig13121: 67,521-69,022

MDDINNNFTLSQDVNGPLLAAVIGMEMLAGLITNSFVLILTACHLKNWKQPTTVFLSNMLANNLVVILFTMPLSIITT
ASGEWIFGSTVSQKESVCYFAAFIFIFSILTATESLVLLSFDRFFFIVKALHYKKYMTVNRAFIIVAVSWLLAAFLSM
LPFFGFGAFEFASSYGMCVPGWTGQAGYAIFSFIVISIFIGSITVTSLWTMCFTRKYLKNAATNISTAASPGNPYAAQ
ERRVIGLFGMLIIVHILCYAPIVSFGLIEIFTDVLTSAAYAVAFVVLLLLTVLIPLVQSFFRRDIRDAIVKGHRTFFR
SIKQEKTYHQSSSTQTIFYFLY

Aq1 >PAC:15712452 ID:712452 scaffold:Aqu1:Contig13121:72652:76703:1
gene:Aqu1.213924

MDDINNNFTLSEDVNGPLLAAVIGIEMLAGLTTNSFVLILTACHLKNWKQPTTVFLSNMLANNLVVILFTMPLSIITT
ATGEWIFGSTVSQKESVCYFAACIFIFSILTATESLVLLSFDRFFFIVKALQYKKYMTVNKAFIIAAVSWLLAAFLSM
LPFFGFGAFEFSFSYVISIFIGSITVTSIWTMCFTRKYLKNAATNISTAASPGNPYAAQERRVIGLFAAYAVAFVVLL
LLTVLIPLVQSFFRRDIKDAIVKGHRTLSRSIKRGKTYHQSSSAQTLRSACGLFNHKASHLSFLNSIMSLSGESDKRV
DPTTNKVVLVGDETVGKTSMFLRFKTDRFIETTSSTRYLAEHYKEWTVRGTKVQMTLYDTAGMERYTSTIPPTYFRHA
RAVILVYAVDNQESIGNIRNWSESFSKHRIGDTVDSLKVLLVGNKVDLNRTVSANRVNEVAEFCGIDNSLKYEISTKN
NDGGFDELFDDLAYLLSDTPIERRKTIRATSSPDDEEMKKKKALCSKCS

→ Aq2.1 Gene: Aqu2.1.21489 Scaffold Contig13121: 72,586-74,098

MDDINNNFTLSEDVNGPLLAAVIGIEMLAGLTTNSFVLILTACHLKNWKQPTTVFLSNMLANNLVVILFTMPLSIITT
ATGEWIFGSTVSQKESVCYFAACIFIFSILTATESLVLLSFDRFFFIVKALQYKKYMTVNKAFIIAAVSWLLAAFLSM
LPFFGFGAFEFSFSYVISIFIGSITVTSIWTMCFTRKYLKNAATNISTAASPGNPYAAQERRVIGLFAAYAVAFVVLL
LLTVLIPLVQSFFRRDIKDAIVKGHRTLSRSIKRGKTYHQSSSAQTILFHVHIENTIYCVHNDVHITTLQIFNICRDK
PQLMNWVLG

→ AND Aq2.1 Gene: Aqu2.1.21490 Scaffold Contig13121: 74,426-76,734

MSLSGESDKRVDPTTNKVVLVGDETVGKTSMFLRFKTDRFIETTSSTRYLAEHYKEWTVRGTKVQMTLYDTAGMERYT
STIPPTYFRHARAVILVYAVDNQESIGNIRNWSESFSKHRIGDTVDSLKVLLVGNKVDLNRTVSANRVNEVAEFCGID
NSLKYEISTKNNDGGFDELFDDLAYLLSDTPIERRKTIRATSSPDDEEMKKKKALCSKCS

Aq1 >PAC:15712786 ID:712786 scaffold:Aqu1:Contig13148:95889:96875:1
gene:Aqu1.214258

MSDVFYNFTESNEGVYGPAVAVILGLEMVAGLFANAVVLCITLMQRKSLSQPSTIFFTSLTAAHLLMVLLYLPVSVIA
IGAEEWIFGSNFEEKKITCSISAFVFWYSVLLMTINLAVISFDRFLFIVKPHFYKQFMRPTVALILTIAVWILSAALN
STPLYGLGAYQYGPSYGSCIPTWRNVPGYLSFMAAIFLIVLAFIIVTSLWTFCFTRKFMHEQEPSQQSNVYQSKERRL
FGIFGAMLIVYGVCFLPSVITGIMTIFVILPGQVYAMDMILFQLITSGSPLVQSYFRPDIKERLTFIYKRSLQMMKNI
FYSRKKNNLSQDEQLS

→ Aq2.1 Gene: Aqu2.1.22066 Scaffold Contig13148: 95,870-96,875

MSDVFYNFTESNEGVYGPAVAVILGLEMVAGLFANAVVLCITLMQRKSLSQPSTIFFTSLTAAHLLMVLLYLPVSVIA
IGAEEWIFGSNFEEKKITCSISAFVFWYSVLLMTINLAVISFDRFLFIVKPHFYKQFMRPTVALILTIAVWILSAALN
STPLYGLGAYQYGPSYGSCIPTWRNVPGYLSFMAAIFLIVLAFIIVTSLWTFCFTRKFMHEQEPSQQSNVYQSKERRL
FGIFGAMLIVYGVCFLPSVITGIMTIFVILPGQVYAMDMILFQLITSGSPLVQSYFRPDIKERLTF
IYKRSLQMMKNIFYSRKKNNLSQDEQLS

Aq1 >PAC:15712925 ID:712925 scaffold:Aqu1:Contig13160:52203:53790:1
gene:Aqu1.214397

MDDDLMSGSGNGLINETEFDFSCRTSVLSTTNVTDQEFWTFRGNLGSGTIAIAFFESIIFIVALSWNLFIFVTYLLKY
RLLKEPANIMLFTLSIVDLLVCILIIPFPIIVVAANGEYIFGNSDVVRCIICQVQGYFFILSTELSLHLLAILSIDRC
ILLSNPLKYKDIKKVWTTVVGILVIWVFCFLLALPPAFGFGEWEFNRSFGVCIPRWTPFRNSLYMILLMIEGLIPIIT
LAVTNVWTFKIVNRFLKKNLERKKSFRATREEVAVEKSTHRSQQNQLVKVFGALFIANIISWTPLLSLTFAIAATDGD
GIPAWLLIVGWFMFLLNPTVHPILESFFIKELRTRVNRASNSVKQQVRRASRSILKMATLDSFKDIPTMNDEDESEKS
RRVFGLKRKSTSQSIGNASVNTSMTDASPPDSPMGITRANTLTNSGRFPHRNRRSSSPKDNIIGTEILPPSPLLTPVS
AALPRISEDSQLALTDIAKSQNNVKPKASSITDLSGKKKKRHISITVPGEKDVYRPEDRDS

→ Aq2.1 Gene: Aqu2.1.22306 Scaffold Contig13160: 50,640-54,289

MDDDLMSGSGNGLINETEFDFSCRTSVLSTTNVTDQEFWTFRGNLGSGTIAIAFFESIIFIVALSWNLFIFVTYLLKY
RLLKEPANIMLFTLSIVDLLVCILIIPFPIIVVAANGEYIFGNSDVVRCIICQVQGYFFILSTELSLHLLAILSIDRC
ILLSNPLKYKDIKKVWTTVVGILVIWVFCFLLALPPAFGFGEWEFNRSFGVCIPRWTPFRNSLYMILLMIEGLIPIIT
LAVTNVWTFKIVNRFLKKNLERKKSFRATREEVAVEKSTHRSQQNQLVKVFGALFIANIISWTPLLSLTFAIAATDGD
GIPAWLLIVGWFMFLLNPTVHPILESFFIKELRTRVNRASNSVKQQVRRASRSILKMATLDSFKDIPTMNDEDESEKS
RRVFGLKRKSTSQSIGNASVNTSMTDASPPDSPMGITRANTLTNSGRFPHRNRRSSSPKDNIIGTEILPPSPLLTPVS
AALPRISEDSQLALTDIAKSQNNVKPKASSITDLSGKKKKRHISITVPGEKDVYRPEDRDSGTVSRSPSPSPSSSNDS
AIVSGNENDNNNNNDSHHETTANNANNDDDTTTATTTANNKISSPLQSNGVNSDNGPCNLISIKAAGEIAIDIDQTVS
DSD

Aq1 >PAC:15713478 ID:713478scaffold:Aqu1:Contig13191:66817:68024:1
gene:Aqu1.214950

MENANFTLSDEINGPLLAGAIGIVTVVALLTNSFVLLLTLCHFKAWKQPSNIFLTNMLLSNLLISVFLMPLCVVTCAT
GEWITGATDAQKLKTCQATAYIFLYNLIVETESLTLISLDRLFFIVKSMEYHKYMSTKKALLIVLLSWLLAAILSTPP
FYGLGSFEFAESYGICVPGFEGQLGFSIYLFLILLSLGGIIVISSTWTFCYTRNFLRRRNSRKTARNSVYLSQKRKLI
GLFGTLVVIHILCYSLMLSLAVFGPFFTISPPWYAAAFFMLLLMTNLSPLAQSYFRYEVRNFVHSLFIKIGIVKLRAS
TLVQQERTATTELPSPTETRRDTIHSTNCQFLNGESEIDKHVV

→ Aq2.1 Gene: Aqu2.1.23215 Scaffold Contig13191: 66,192-68,058

MENANFTLSDEINGPLLAGAIGIVTVVALLTNSFVLLLTLCHFKAWKQPSNIFLTNMLLSNLLISVFLMPLCVVTCAT
GEWITGATDAQKLKTCQATAYIFLYNLIVETESLTLISLDRLFFIVKSMEYHKYMSTKKALLIVLLSWLLAAILSTPP
FYGLGSFEFAESYGICVPGFEGQLGFSIYLFLILLSLGGIIVISSTWTFCYTRNFLRRRNSRKTARNSVYLSQKRKLI
GLFGTLVVIHILCYSLMLSLAVFGPFFTISPPWYAAAFFMLLLMTNLSPLAQSYFRYEVRNFVHSLFIKIGIVKLRAS
TLVQQERTATTELPSPTETRRDTIHSTNCQFLNGESEIDKHVV

Aq1 >PAC:15713938 ID:713938 scaffold:Aqu1:Contig13214:46818:48119:1
gene:Aqu1.215410

MDSLDDDQVLSANENFTISPSISGPAIAFFLSLEMGISLAINSTILGIIFLHPKPRSLIKAPSNIYLTSMLLVNLLAT
LTVMPMIIIASASGEWIFGGTLEEKVSSCRFAGYMYWYVIFLLIVTLAIISVDRWMFIVKSTLYKKMMTTPVAIGVVI
TAWLLTAVLNTTPLYGFGMFAYSSSPGSCFPVWKDQKAYLAFFVLVFLILISIIAASSIWTFCFSRRFIRRMSLRNID
ASGEAASVYTFRWRKLIGIFGTLSIVYVVCFTPSLLYLLIRLATPIPLQILPAILIFFLLITILNPLVQIFFRRDIKN
VILDFLAKTPCYTKEEVDYEHRSRTRSNFELQHALSLTAMGKDVANGGSALDVSPDEGHSSDPKSSGKVVVKFEDSES
VPVQANGATSVGGDATNDSASSESVSNGVSKTNGGDILGSTAV

→ Aq2.1 Gene: Aqu2.1.23876 Scaffold Contig13214: 46,783-48,365

MDSLDDDQVLSANENFTISPSISGPAIAFFLSLEMGISLAINSTILGIIFLHPKPRSLIKAPSNIYLTSMLLVNLLAT
LTVMPMIIIASASGEWIFGGTLEEKVSSCRFAGYMYWYVIFLLIVTLAIISVDRWMFIVKSTLYKKMMTTPVAIGVVI
TAWLLTAVLNTTPLYGFGMFAYSSSPGSCFPVWKDQKAYLAFFVLVFLILISIIAASSIWTFCFSRRFIRRMSLRNID
ASGEAASVYTFRWRKLIGIFGTLSIVYVVCFTPSLLYLLIRLATPIPLQILPAILIFFLLITILNPLVQIFFRRDIKN
VILDFLAKTPCYTKEEVDYEHRSRTRSNFELQHALSLTAMGKDVANGGSALDVSPDEGHSSDPKSSGKVVVKFEDSES
VPVQANGATSVGGDATNDSASSESVSNGVSKTNGGDILGSTAV

Aq1 >PAC:15713939 ID:713939 scaffold:Aqu1:Contig13214:48793:49833:1
gene:Aqu1.215411

MDYEDDETLFILEENYNFTLNPLINGPALASIVGLELFAGFITNSLLLALTVFQWKNWKLPSTVFLTNLLLCNLLLVL
LVMPFTIITGATGEWVFGRNPVEKRTVCRIIAFIFWYGITVFTFGLVILSFDRFFYVVKAVAYKNRMTIKKAVTIVIA
TWVAGGALNITPFFGMGKYSFIACCGVCVPRWAGEPEYIVYILIIYVTCILSIVVTSIWTCIFTRRFLGKDERRTRFV
SQNTRSHLYATKNQRLLGLFGMTVLVHLLCYLPGISSSFLELFIPVPVQLYTTMYVAFLMAATLGPLVQSFFRRDIRE
TLSQLCGEKFKVFAVNRSFISSSSIKVNTNSTCL

→ Aq2.1 Gene: Aqu2.1.23877 Scaffold Contig13214: 48,545-50,010

MDYEDDETLFILEENYNFTLNPLINGPALASIVGLELFAGFITNSLLLALTVFQWKNWKLPSTVFLTNLLLCNLLLVL
LVMPFTIITGATGEWVFGRNPVEKRTVCRIIAFIFWYGITVFTFGLVILSFDRFFYVVKAVAYKNRMTIKKAVTIVIA
TWVAGGALNITPFFGMGKYSFIACCGVCVPRWAGEPEYIVYILIIYVTCILSIVVTSIWTCIFTRRFLGKDERRTRFV
SQNTRSHLYATKNQRLLGLFGMTVLVHLLCYLPGISSSFLELFIPVPVQLYTTMYVAFLMAATLGPLVQSFFRRDIRE
TLSQLCGEKFKVFAVNRSFISSSSIKVNTNSTCL

Aq1 >PAC:15713940 ID:713940 scaffold:Aqu1:Contig13214:53879:54397:1
gene:Aqu1.215412

MIGGLIANSFVLILTICHIKTWKQPSTLFLTNMLISNLLIVLFVRPFSITTAASGEWLFGKTYKQKVKVCQFTAFIFW
FCVIVITEGLVLLSFDRFFYIVKSFEYERHMNRKISIIIVTLSWLLAALLTIPPLFGLGRFGFSSSYGICVPHWEGES
GYVVYMLMVFIIFIKS

→ Aq2.1 Gene: Aqu2.1.23879 Scaffold Contig13214: 51,883-54,397

MDDNFTLSADINGPLLAAVISIEMIGGLIANSFVLILTICHIKTWKQPSTLFLTNMLISNLLIVLFVRPFSITTAASG
EWLFGKTYKQKVKVCQFTAFIFWFCVIVITEGLVLLSFDRFFYIVKSFEYERHMNRKISIIIVTLSWLLAALLTIPPL
FGLGRFGFSSSYGICVPHWEGESGYVVYMLMVFIIFIKS

Aq1 >PAC:15713942 ID:713942 scaffold:Aqu1:Contig13214:57845:58461:1
gene:Aqu1.215414

LRKMEDMDENFTLSGDINGPLLAAVISIEMIAGLIANSFVLILTICHIKTWKQLSTIFLTNMLISNLLIVLFVMLFSI
TTAASGEWLFGRTYKQKMKVCQFTGFMFWFCVIVITEGLVLLSFDRFFYIVKSFEYERHMNQKISIIIVTLSWLLAAL
LTIPPLFGLGRFSFSSSYGICVPHWEGESGYVVYMLIVFIIFIISLHN

→ Aq2.1 Gene: Aqu2.1.23880 Scaffold Contig13214: 57,856-58,461

MEDMDENFTLSGDINGPLLAAVISIEMIAGLIANSFVLILTICHIKTWKQLSTIFLTNMLISNLLIVLFVMLFSITTA
ASGEWLFGRTYKQKMKVCQFTGFMFWFCVIVITEGLVLLSFDRFFYIVKSFEYERHMNQKISIIIVTLSWLLAALLTI
PPLFGLGRFSFSSSYGICVPHWEGESGYVVYMLIVFIIFIISLHN

Aq1 >PAC:15713946 ID:713946 scaffold:Aqu1:Contig13214:63443:64519:1
gene:Aqu1.215418

MRNSNEYLKYFLFRPKSSSSMDDIIELNHNFTLSGDINGPLLAAVISVEMIGGLIANSFVLILTICHIKTWKQPSTIF
LTNMLISNLLIVLFVMPFPITTCSNGKWMLGETEAEQVKYCYFAGYLYWYSVLLITQSLVILSFDRFFYIVKSFMYER
YMTGKRSLSIVAFSWLIASILNITPFIGLGSFGFINSYGSCGPLWEEEMGHVIYTFIIFIFYIGSIVTTTLWTYCYTR

RFLQSERLRTEFISRIPQQCSVYISKEKRLIGLFGMMMVVYLTCYAPGLVAIVAVIFTPLPQPVYASVYVLFLLITVL
SPLVQIIFRRDMREAVSKIIKCQRKEGASKCERQNSRSFMLTHLHT

→ Aq2.1 Gene: Aqu2.1.23884 Scaffold Contig13214: 63,443-64,519

MRNSNEYLKYFLFRPKSSSSMDDIIELNHNFTLSGDINGPLLAAVISVEMIGGLIANSFVLILTICHIKTWKQPSTIF
LTNMLISNLLIVLFVMPFPITTCSNGKWMLGETEAEQVKYCYFAGYLYWYSVLLITQSLVILSFDRFFYIVKSFMYER
YMTGKRSLSIVAFSWLIASILNITPFIGLGSFGFINSYGSCGPLWEEEMGHVIYTFIIFIFYIGSIVTTTLWTYCYTR
RFLQSERLRTEFISRIPQQCSVYISKEKRLIGLFGMMMVVYLTCYAPGLVAIVAVIFTPLPQPVYASVYVLFLLITVL
SPLVQIIFRRDMREAVSKIIKCQRKEGASKCERQNSRSFMLTHLHT

Aq1 >PAC:15713947 ID:713947 scaffold:Aqu1:Contig13214:64616:65617:1
gene:Aqu1.215419

MDDEQQDVLLRNYNFTLSADINGPLLAAVISIEMIAGLIANSFVLIITICHCSSGTWKKPSTLFLTNMLICNLFTVLL
VMPLNIISLASRGWIFGVTVDQKLITCNLAGYIYWNSVLLISLSLMLLSIDRWIYIVQAMRYDSLVTPRRALLAIIIT
WSLAIVLNVTPFFGFGTYNFLESYAGCSPIWEGHVGYVVYMLIIFVSIVGTIVVTSCWTFCYLFMYIRRRKERSSIGP
GRKRSIDQYVAARRKLIVLFGVLLFIHFISYLPGIITAGVVIFVSLPKEVYATSYILFLSITTLSPLAQSCFREDIRS
KLCKKKEEPPGTVDSSPFTDN

→ Aq2.1 Gene: Aqu2.1.23885 Scaffold Contig13214: 64,588-65,730

MDDEQQDVLLRNYNFTLSADINGPLLAAVISIEMIAGLIANSFVLIITICHCSSGTWKKPSTLFLTNMLICNLFTVLL
VMPLNIISLASRGWIFGVTVDQKLITCNLAGYIYWNSVLLISLSLMLLSIDRWIYIVQAMRYDSLVTPRRALLAIIIT
WSLAIVLNVTPFFGFGTYNFLESYAGCSPIWEGHVGYVVYMLIIFVSIVGTIVVTSCWTFCYLFMYIRRRKERSSIGP
GRKRSIDQYVAARRKLIVLFGVLLFIHFISYLPGIITAGVVIFVSLPKEVYATSYILFLSITTLSPLAQSCFREDIRS
KLCKKKEEPPGTVDSSPFTDN

Aq1 >PAC:15714975 ID:714975 scaffold:Aqu1:Contig13264:50611:51555:1
gene:Aqu1.216447

MEMDGNFTLSDRRDGIALAVVLTIEVILAAIANGAVLCITIYQRKHWRQPSTIFFTSLILAHLVMMLLYLPFTITALA
AGEWSIGRTNEQRIGTCFFSIFLYMYTVLIVLMTLAAISFDRFLFIVKPHLHKRFMKLWVAVSLAVAIWILSAVLNVI
PLFSFEELTYDDTFGPCYPSYLNTLAYALFIGIIFVTMIAIIVVTCTWTFCFTRKFLKDQSIIAGESVYSAKKRRLFG
IFGSMLLVYIICFTPGGIHLALIPIIDTPFGLYATSLASYHFITIANPLVQSYFRPEIKSVFVSAYHKMSPVSVTVND
TV

→ Aq2.1 Gene: Aqu2.1.25432 Scaffold Contig13264: 50,611-51,555

MEMDGNFTLSDRRDGIALAVVLTIEVILAAIANGAVLCITIYQRKHWRQPSTIFFTSLILAHLVMMLLYLPFTITALA
AGEWSIGRTNEQRIGTCFFSIFLYMYTVLIVLMTLAAISFDRFLFIVKPHLHKRFMKLWVAVSLAVAIWILSAVLNVI
PLFSFEELTYDDTFGPCYPSYLNTLAYALFIGIIFVTMIAIIVVTCTWTFCFTRKFLKDQSIIAGESVYSAKKRRLFG
IFGSMLLVYIICFTPGGIHLALIPIIDTPFGLYATSLASYHFITIANPLVQSYFRPEIKSVFVSAYHKMSPVSVTVND
TV

Aq1 >PAC:15716328 ID:716328 scaffold:Aqu1:Contig13317:77929:78861:1
gene:Aqu1.217800

MEGNFTFTGEFSGPAVAAVFTVEMILALIANGVVLSITLYQWKSFKQSSIIFFTSLILAHLVLNLLNLPFTMIPLAAG
EWIFGITNEEKRGTCHFAAWMNWSGSYVLSFTLAAISFDRFLFIVKPHLHTRFMRPCVALTLTIAIWILSAVLGTLPF
FDIGHYSYFDKLGLCSLVGVDIAAFVVILVIIFLVVGTIFITSLWTFFFTFKAQSVIAGENVYTSKKKRLFGIFGSML
IIYGTGYLIAALGFLLRIFIFLPYEFFITSHIVYSFVTVASPIIQSYFRPEIKSVLVSRCPLLFTCVCCSCIHAVC

→ Aq2.1 Gene: Aqu2.1.27374 Scaffold Contig13317: 77,929-78,861

MEGNFTFTGEFSGPAVAAVFTVEMILALIANGVVLSITLYQWKSFKQSSIIFFTSLILAHLVLNLLNLPFTMIPLAAG
EWIFGITNEEKRGTCHFAAWMNWSGSYVLSFTLAAISFDRFLFIVKPHLHTRFMRPCVALTLTIAIWILSAVLGTLPF
FDIGHYSYFDKLGLCSLVGVDIAAFVVILVIIFLVVGTIFITSLWTFFFTFKAQSVIAGENVYTSKKKRLFGIFGSML

IIYGTGYLIAALGFLLRIFIFLPYEFFITSHIVYSFVTVASPIIQSYFRPEIKSVLVSRCPLLFTCVCCSCIHAVC

Aq1 >PAC:15716337 ID:716337 scaffold:Aqu1:Contig13317:147680:148621:1 gene:Aqu1.217809

MEGNFTFTGKFSGQAVAAVFTVEMILALIANGVVLSITLYQKKSWKQSSTIFFTSLILAHLVLNLLHLPFAVIALAAG
EWIFGSTDEEKRGTCTFASYLYWYIVDVISMTLAAISFDRFLFIVKPHLHKWFMRPWVALTLTIAIWILCAVLGTLPF
INIGHYSYDVELGFCSIVGVDIAAFVLILVIIFLVVGTIFVTSLWTFCVTKSYFKDQSVIAGESVYASKKKRLFGIFG
SMLIVYGTAYLIAALGFLLQIFIFLPYEFYVTNYIVFCFVTIASPIIQSYFRPEIKSVLVSRCPLLFTCVCCSCVHSI
Y

→ Aq2.1 Gene: Aqu2.1.27387 Scaffold Contig13317: 147,680-148,621

MEGNFTFTGKFSGQAVAAVFTVEMILALIANGVVLSITLYQKKSWKQSSTIFFTSLILAHLVLNLLHLPFAVIALAAG
EWIFGSTDEEKRGTCTFASYLYWYIVDVISMTLAAISFDRFLFIVKPHLHKWFMRPWVALTLTIAIWILCAVLGTLPF
INIGHYSYDVELGFCSIVGVDIAAFVLILVIIFLVVGTIFVTSLWTFCVTKSYFKDQSVIAGESVYASKKKRLFGIFG
SMLIVYGTAYLIAALGFLLQIFIFLPYEFYVTNYIVFCFVTIASPIIQSYFRPEIKSVLVSRCPLLFTCVCCSCVHSI
Y

Aq1 >PAC:15716432 ID:716432 scaffold:Aqu1:Contig13321:99445:100431:-1 gene:Aqu1.217904

MERNYTFTGDFSPEAVAAVLSIEMILALIANGVVLVITIYQRKSWKQSSTILFTSLILAHLLIILVLPFCIAALAAGE
WIIGSTDEERKGTCDVSAYILLYSGNIMYMTLSLISIDRFLFIVKPHLHKRFMSPRVALVLVIIVWIATALFFSSGFI
NGSGTVFKYINNVGACYGYSASPIMAAIRFSIITILLSIIIITSVWTFCFTRKFINNQSMIVGESVYASKKKRLFGIF
GSMLLVYGICFAPGTFLSSFFAIIDAPDKLNISALVLYLLAFILSPVIQSYFRPEINSVIVNIIYHKMMKKLNQSYYQ
TTSSNTSKTNTNSLDL

→ Aq2.1 Gene: Aqu2.1.27518 Scaffold Contig13321: 99,445-100,431

MERNYTFTGDFSPEAVAAVLSIEMILALIANGVVLVITIYQRKSWKQSSTILFTSLILALLIILVLPFCIAALAAGEW
IIGSTDEERKGTCDVSAYILLYSGNIMYMTLSLISIDRFLFIVKPHLHKRFMSPRVALVLVIIVWIATALFFSSGFIN
GSGTVFKYINNVGACYGYSASPIMAAIRFSIITILLSIIIITSVWTFCFTRKFINNQSMIVGESVYASKKKRLFGIFG
SMLLVYGICFAPGTFLSSFFAIIDAPDKLNISALVLYLLAFILSPVIQSYFRPEINSVIVNIIYHKMMKKLNQSYYQT
TSSNTSKTNTNSLDL

Aq1 >PAC:15716433 ID:716433 scaffold:Aqu1:Contig13321:101088:102068:-1 gene:Aqu1.217905

MERNYTFTGDFSPQAVAGVLSIEMILALIANGVVLVITIYQRKSWKQSSTIFFTSLILAHLFMILYLPFTITALAAGE
WIIGSTDEEKEGTCDFTGFLIIYCVYVMLMILSLISIDRFLFIVKPHLHKRFMSPRVALVLVIIVWIVTAVLFSSGFI
NGSGIVYQYVENLGGCYVYTTGPIASVIRYLFASLILSIIIITSIWTFCFTRKFINNQSMIVGESVYASKKKRLFGIF
GSMILVYGICFAPSVFLSSFMTTIDLPDELHVFSLVLLFLVVTLSPVVQSYFRPEIKSVIVDVICHKMMRKPNSSDPG
VTQDTNINYNTIGP

→ Aq2.1 Gene: Aqu2.1.27516 Scaffold Contig13321: 95,043-102,092

LSKLAKERMERNYTFTGDFSPQAVAGVLSIEMILALIANAAGEWIIGSTDEEKEGTCDFTGFLIIYCVYVMLMILSLI
SIDRFLFIVKPHLHKRFMSPRVALVLVIIVWIVTAVLFSSGFINGSGIVYQYVENLGGCYVYTTGPIASVIRYLFASL
ILSIIIITSIWTFCFTLGESVYASKKKRLFGIFGSMLLVYGICFAPAKVNAPDKLNISALVLFLLALILSPVVQSYFR
PEINSVIVNIICRKIMKKRNRTTPSN

Aq1 >PAC:15716434 ID:716434 scaffold:Aqu1:Contig13321:103086:104042:-1 gene:Aqu1.217906

MERNYTFTGDFSPEAVAAVFSIEIILALIANGVVLVISIHQRKSWKESSTIFFTSLILAHLLMLLVIPFSITSLAAGE
WIIGSTDEEKEGSCGFTAYITYCSVYNTYVTLSLISIDRFLFIVKPHLHKRFMSPRVALVLVIIVWIVTAVLLSSGFI
DGSGLTYQYIDNIGGCYVGITSPIASIFRSSSASIILSIILITSVWTFCFTRKFINNQSMIVGESVYATKKKRLFGIF

GSMLLVYGMCFIPATFLQSFLTIIDAPDKLIVSSLVLFLLGIILSPVVQSYFRPEINRIIVDVICHKMMHTPTPTDPN
TSNNLA

→ Aq2.1 Gene: Aqu2.1.27519 Scaffold Contig13321: 103,086-104,042

MERNYTFTGDFSPEAVAAVFSIEIILALIANGVVLVISIHQRKSWKESSTIFFTSLILAHLLMLLVIPFSITSLAAGE
WIIGSTDEEKEGSCGFTAYITYCSVYNTYVTLSLISIDRFLFIVKPHLHKRFMSPRVALVLVIIVWIVTAVLLSSGFI
DGSGLTYQYIDNIGGCYVGITSPIASIFRSSSASIILSIILITSVWTFCFTRKFINNQSMIVGESVYATKKKRLFGIF
GSMLLVYGMCFIPATFLQSFLTIIDAPDKLIVSSLVLFLLGIILSPVVQSYFRPEINRIIVDVICHKMMHTPTPTDPN
TSNNLA

Aq1 >PAC:15716436 ID:716436 scaffold:Aqu1:Contig13321:108135:109154:-1
gene:Aqu1.217908

MERNYTFTGDFSPEAVAGVLSIEMILALIANGVVLVITIYQRKSWKQSSTIFFTSLILAHLVLTLYLPFSIAALAAHK
WIIGSTDEEKKGTCDFNGFVILFSAYVMFMTLSLISIDRFLFIVKPHLHKGFMSPRVALVLVIIVWIVNAVFFSSGFI
NGSGMVYQYIDSQGLCYANTTSPIMAATRFSMAMILLFIIIITSVWTFCFTRKFINNQSMIVGESVYATKKKRLFGIF
GSMILVYXXXXIFGSMILVYGICFTPGTFLSSFLAIIDVPDQLTISSVILFFLAIILSPVVQSYFRPEINSVIVNIIC
HKIMHKPDHNLTVKRDASCTNIASFDF

→ Aq2.1 Gene: Aqu2.1.27520 Scaffold Contig13321: 108,135-109,154

MERNYTFTGDFSPEAVAGVLSIEMILALIANGVVLVITIYQRKSWKQSSTIFFTSLILAHLVLTLYLPFSIAALAAHK
WIIGSTDEEKKGTCDFNGFVILFSAYVMFMTLSLISIDRFLFIVKPHLHKGFMSPRVALVLVIIVWIVNAVFFSSGFI
NGSGMVYQYIDSQGLCYANTTSPIMAATRFSMAMILLFIIIITSVWTFCFTRKFINNQSMIVGESVYATKKKRLFGIF
GSMILVYXXXXIFGSMILVYGICFTPGTFLSSFLAIIDVPDQLTISSVILFFLAIILSPVVQSYFRPEINSVIVNIIC
HKIMHKPDHNLTVKRDASCTNIASFDF

Aq1 >PAC:15716437 ID:716437 scaffold:Aqu1:Contig13321:111574:112554:-1
gene:Aqu1.217909

MERNYTFTGDFSPEAVAGVLSIEMILALIANGVVLVITIYQRKSWKQSSTIFFTSLILAHLVLTLYLPFSIATLAAGE
WIIGSTDEEKKGTCNFNGFIVLFSAYVMFMTLSLISIDRFLFIVKPHLHKRFMSPRVALVLVIIVWIVNAVFFSSGFI
NGSGMVYQYIDSQGLCFANTTSPIMAATRFSMAMILLCIIVVTSVCTFCFTRKFINNQSMIVGESVYASKKKRLFGIF
GSMLLIYGICFAPGTFLSSFLAIIDPPNQLIISAVILFFLAIILSPVVQSYFRPEINSVIVNIICHKIMDKPDHNLTV
KHDSSCTNTVSFDS

→ Aq2.1 Gene: Aqu2.1.27522 Scaffold Contig13321: 111,555-112,554

MERNYTFTGDFSPEAVAGVLSIEMILALIANGVVLVITIYQRKSWKQSSTIFFTSLILAHLVLTLYLPFSIATLAAGE
WIIGSTDEEKKGTCNFNGFIVLFSAYVMFMTLSLISIDRFLFIVKPHLHKRFMSPRVALVLVIIVWIVNAVFFSSGFI
NGSGMVYQYIDSQGLCFANTTSPIMAATRFSMAMILLCIIVVTSVCTFCFTRKFINNQSMIVGESVYASKKKRLFGIF
GSMLLIYGICFAPGTFLSSFLAIIDPPNQLIISAVILFFLAIILSPVVQSYFRPEINSVIVNIICHKIMDKPDHNLTV
KHDSSCTNTVSFDS

Aq1 >PAC:15716438 ID:716438 scaffold:Aqu1:Contig13321:112825:114269:-1
gene:Aqu1.217910

MERNYTFTGDFSPEAVAGVLSIEMILALIANGVVLVITIYQRKSWKQSSTIFFTSLILAHLVLTLYLPFSIAALAARE
WIIGSTDEEKQGTCDFIGFIVQFSAYVMYMTLSLISIDRFLFIVKPHLHKRFMSPRVALVLVIIVWIDLSMDLFGRFV
SLSMIVGESVYASKKKRLFGIFGSMILVYGICFTPATFLAAFLVIIGPPNQLIISALILFFLAIILSPVVQSYFRPEI
NSVIHLKPKSILEMIL

→ Aq2.1 Gene: Aqu2.1.27523 Scaffold Contig13321: 113,778-114,287

MFKLRKMERNYTFTGDFSPEAVAGVLSIEMILALIANAAREWIIGSTDEEKQGTCDFIGFIVQFSAYVMYMTLSLISI
DRFLFIVKPHLHKRFMSPRVALVLVIIVWIVNAVFFSSGFINGSGIWY

Aq1 >PAC:15716439 ID:716439 scaffold:Aqu1:Contig13321:117887:118867:-1 gene:Aqu1.217911

MERNYTFTGDFSPEAVAAVLSIEMILALIANGVVLVITIYQRKSWKQSSTIFFTSLILAHLTLTLYLPFSIAALAARE
WIIGDTDEEKKATCDFNGFMILYCAYNMLMTLSLISIDRFLFIVKPHLHKRFMSPRIALVLVNIVWIVNAAFFSSGFI
NGSGMVYQYIDNVGLCYAFTTSPIMAGTRFSMIMILLCIIIITSIWTFCFTRKFINNQSMIVGESVYASKKKRLFGIF
GSMILVYGICFAPATFLSSFLAIIDSPNQLIISAVILFFLAIILSPVVQSYFRPEINSVIVNIICHKMKHKPGHNLTV
KRDASCTNTASFDF

→ Aq2.1 Gene: Aqu2.1.27524 Scaffold Contig13321: 117,930-118,225

SMIVGESVYASKKKRLFGIFGSMILVYGICFAPATFLSSFLAIIDSPNQLIISAVILFFLAIILSPVVQSYFRPEINS
VIVNIICHKMKHKPGHNLTV

AND
→ Aq2.1 Gene: Aqu2.1.27525 Scaffold Contig13321: 118,390-118,867

MERNYTFTGDFSPEAVAAVLSIEMILALIANGVVLVITIYQRKSWKQSSTIFFTSLILAHLTLTLYLPFSIAALAARE
WIIGDTDEEKKATCDFNGFMILYCAYNMLMTLSLISIDRFLFIVKPHLHKRFMSPRIALVLVNIVWIDSSMDP

Aq1 >PAC:15716440 ID:716440 scaffold:Aqu1:Contig13321:119695:121318:-1 gene:Aqu1.217912

MERNYTFTGDFSPEAVAAIFSIEMILALIANGVVLVITIYQRKSWKQSSTIFFTSLILAHLLIILVLPFSITALAAGE
WIIGSTDEERKGTCDVSAYILLYSGNIMYITLSLISIDRFLFIVKPHLHKRFMSPRVALVLVIIVWIATALFFSSGFI
NGSGTVFEYINDIGACYAPVVQSYFRPEINSVIVNIICRKMRKKLNRTTPTAGEWIIGDTDKEKKGTCGFNGFMILYC
AYNMLMTLSLISIDRFLFIVKPHLHKRFMSPRVALVLVIIVLIVTAVLLSSGFINGSGIVYQYIDSQGLCYANTTSPI
MAATCFSMTMILLSIIVVTSIWTFCFTRKFIKIKKYKQQQVKTYKDYRNTTTVQYY

→ Aq2.1 Gene: Aqu2.1.27526 Scaffold Contig13321: 119,691-121,318

MERNYTFTGDFSPEAVAAIFSIEMILALIANGVVLVITIYQRKSWKQSSTIFFTSLILAHLLIILVLPFSITALAAGE
WIIGSTDEERKGTCDVSAYILLYSGNIMYITLSLISIDRFLFIVKPHLHKRFMSPRVALVLVIIVWIATALFFSSGFI
NGSGTVFEYINDIGACYAPVVQSYFRPEINSVIVNIICRKMRKKLNRTTPTAGEWIIGDTDKEKKGTCGFNGFMILYC
AYNMLMTLSLISIDRFLFIVKPHLHKRFMSPRVALVLVIIVLIVTAVLLSSGFINGSGIVYQYIDSQGLCYANTTSPI
MAATCFSMTMILLSIIVVTSIWTFCFTRKFIKIKKYKQQQVKTYKDYRNTTTVQYY

Aq1 >PAC:15716509 ID:716509 scaffold:Aqu1:Contig13324:109378:110541:1 gene:Aqu1.217981

MNVTEVPGFQKGLFVFFWLISIGLNTLCSALLMIAVHKRKSWANILLLSIALTGIAAVLLGMPLSIVSLFINDLLIDE
GALCQYQVIVFNWYILMSFFLVVFISIDQYLAVLYPFMYNTKILRHPKRSLKIIRILLFFVTSGTLSYSIIGSFLSTI
AVMTPSNICYYDFLAVDTPSKIFFIVNTIAMGLMILVILTCTILLAIKMYRMFLKAEDPKDTFQVQKANFGHFLLVII
IVFMAAASLFLVVHILYVAGYSVSVYLHYLSLCLVLSNSLLFPLIFILIRASVRQILKEIWKKLLYCREDKETTGKRQ
DKAVQLKQLKKSY

→ Aq2.1 Gene: Aqu2.1.27628 Scaffold Contig13324: 109,378-110,541

MNVTEVPGFQKGLFVFFWLISIGLNTLCSALLMIAVHKRKSWANILLLSIALTGIAAVLLGMPLSIVSLFINDLLIDE
GALCQYQVIVFNWYILMSFFLVVFISIDQYLAVLYPFMYNTKILRHPKRSLKIIRILLFFVTSGTLSYSIIGSFLSTI
AVMTPSNICYYDFLAVDTPSKIFFIVNTIAMGLMILVILTCTILLAIKMYRMFLKAEDPKDTFQVQKANFGHFLLVII
IVFMAAASLFLVVHILYVAGYSVSVYLHYLSLCLVLSNSLLFPLIFILIRASVRQILKEIWKKLLYCREDKETTGKRQ
DKAVQLKQLKKSY

Aq1 >PAC:15716749 ID: 716749 scaffold:Aqu1:Contig13334:6674:7678:-1 gene:Aqu1.218221

MSLLELNSTTFTATGEVNGPAFAAAFAIEGLIGLIANITVLAITLYQRKSWKQSSTVFFTSFLLANIITVMYMLVSSI
SIGAGEWIIGSTFEEKRATCTFTAYVIWLDSMALTMTIAAISFDRFLFIVKPHRHKYLMKSWIALIVTISLWVLSAVV
NIAPFYSHDEYKAGIYDSQIGICLKNFIILVTSLWTFCFTCRFMQTQSEMPGNNSVYVSKKWRLFGIFGTMLLAYIIA
VIPAYIFGVISIFYTLPPAVNVTAALAYGSIVISNPIIQSYFRPDIKIVLTAIIKKIKLKVTRRYQNTTQTDANMELE

→ Aq2.1 Gene: Aqu2.1.28010 Scaffold Contig13334: 6,674-7,678

MSLLELNSTTFTATGEVNGPAFAAAFAIEGLIGLIANITVLAITLYQRKSWKQSSTVFFTSFLLANIITVMYMLVSSI
SIGAGEWIIGSTFEEKRATCTFTAYVIWLDSMALTMTIAAISFDRFLFIVKPHRHKYLMKSWIALIVTISLWVLSAVV
NIAPFYSHDEYKAGIYDSQIGICLKNFIILVTSLWTFCFTCRFMQTQSEMPGNNSVYVSKKWRLFGIFGTMLLAYIIA
VIPAYIFGVISIFYTLPPAVNVTAALAYGSIVISNPIIQSYFRPDIKIVLTAIIKKIKLKVTRRYQNTTQTDANMELE

Aq1 >PAC:15716877 ID: 716877 scaffold:Aqu1:Contig13338:4914:5852:1
gene:Aqu1.218349

MDIHGLNYTLGEDVNGPLLAAALAVEMIAALIVNTFVLVATFSQCKSLKLPSTILFTSLIMIHYVIALIYIPSWLISA
AYGEWIFGSTIQVKEATCKFAGFILNYTLQVKSFTLAAISVDRWLFIVKPIFYKQYMKAKLAVVLAVIIWIGSCLSVA
TPFFGIGNYFFTEFGSCEPQFLGELGYSILMLIFVLTALCIIIVTSIWTCCFTRRFIREHAQLADESAYVSKNRRIIG
IFGAMLVAYGVCYTPGIIVIIASLFCDVNAAAFAAVLVFFLTVIIINPIIQSVFRPDVKKVIVKLCAMCRKFHHSPEA

→ Aq2.1 Gene: Aqu2.1.28206 Scaffold Contig13338: 4,914-5,943

MDIHGLNYTLGEDVNGPLLAAALAVEMIAALIVNTFVLVATFSQCKSLKLPSTILFTSLIMIHYVIALIYIPSWLISA
AYGEWIFGSTIQVKEATCKFAGFILNYTLQVKSFTLAAISVDRWLFIVKPIFYKQYMKAKLAVVLAVIIWIGSCLSVA
TPFFGIGNYFFTEFGSCEPQFLGELGYSILMLIFVLTALCIIIVTSIWTCCFTRRFIREHAQLADESAYVSKNRRIIG
IFGAMLVAYGVCYTPGIIVIIASLFCDVNAAAFAAVLVFFLTVIIINPIIQSVFRPDVKKVIVKLCAMCRKFHHSPEA

Aq1 >PAC:15717007 ID:717007 scaffold:Aqu1:Contig13341:69084:70064:1
gene:Aqu1.218479

MSFEHNFTTTGNVNGPALSAVLALETVTAFIANTVVLSITLYQRKSWKQSSTIFFTSLILSHLVMVFLYLPLCVIAVA
AEEWIFGSTFEQQIATCSFAAYVIWYCVLVIQMTLAIISFDRFLFIVKPRIHKRLMRPWTALAFTIGIWILAAILNSG
PFVGLGEYEYDDTYGSCVPLWQGNNGYVLYMLIISMISITIILITSIWTFCFTRRFINDQSQVSGDSVYVSRKKRLFG
IFGSMLLVYGICFLPSIISGGITTVVILPDPVYAVNMVTFQFITVASPLVQSYFRPDIKAAIISFFALFAPALKKWQG
LTSRSHETSTSLPA

→ Aq2.1 Gene: Aqu2.1.28353 Scaffold Contig13341: 69,040-70,228

MSFEHNFTTTGNVNGPALSAVLALETVTAFIANTVVLSITLYQRKSWKQSSTIFFTSLILSHLVMVFLYLPLCVIAVA
AEEWIFGSTFEQQIATCSFAAYVIWYCVLVIQMTLAIISFDRFLFIVKPRIHKRLMRPWTALAFTIGIWILAAILNSG
PFVGLGEYEYDDTYGSCVPLWQGNNGYVLYMLIISMISITIILITSIWTFCFTRRFINDQSQVSGDSVYVSRKKRLFG
IFGSMLLVYGICFLPSIISGGITTVVILPDPVYAVNMVTFQFITVASPLVQSYFRPDIKAAIISFFALFAPALKKWQG
LTSRSHETSTSLPA

Aq1 >PAC:15717374 ID:717374 scaffold:Aqu1:Contig13350:136964:139713:-1
gene:Aqu1.218846

MDEQRLEYLEFYNQSCNVSTINSSSSFGPPIAVIRIIGSILSILGSTSIIIAVFLTGKLSNMEIHPLFSLAVGDFILS
CLWLFGGSLWLDTYQDRYSDIHPGLGLCYILAISTTIVNMVTSFLTIVYSLHAFIVMRQLSKSRGHISRGRTCKQHLI
TAFVYLVAWSLPLLLVLPVTESMIGLDIANNACWCFIDFFNTRPGGGPNTEISDSDDLATIIAVYSGVTFVVTAIAII
ALYAGTLHMARKVLKHQKTLNSEINSSKVIKKLAGRAAAFIIIYLICGLPFLAGAIDVWKENTPLHLLEKPDQTILFW
QAIFGPLQGFLNAIVYGWSRPEFRRLFVTPCKKLRRRMRVKDTHK

→ Aq2.1 Gene: Aqu2.1.28814 Scaffold Contig13350: 136,884-139,801

MDEQRLEYLEFYNQSCNVSTINSSSSFGPPIAVIRIIGSILSILGSTSIIIAVFLTGKLSNMEIHPLFSLAVGDFILS
CLWLFGGSLWLDTYQDRYSDIHPGLGLCYILAISTTIVNMVTSFLTIVYSLHAFIVMRQLSKSRGHISRGRTCKQHLI
TAFVYLVAWSLPLLLVLPVTESMIGLDIANNACWCFIDFFNTRPGGGPNTEISDSDDLATIIAVYSGVTFVVTAIAII
ALYAGTLHMARKVLKHQKTLNSEINSSKVIKKLAGRAAAFIIIYLICGLPFLAGAIDVWKENTPLHLLEKPDQTILFW

QAIFGPLQGFLNAIVYGWSRPEFRRLFVTPCKKLRRRMRVKDTHVLIDSVEEYSYYH

Aq1 >PAC:15718112 ID:718112 scaffold:Aqu1:Contig13373:75281:76844:1 gene:Aqu1.219584

MFLPHYDYDHGNNLSADQGDVSPTQDIGFIIALNISVGIVCLLSILGSTSIIFSYILYKELRTTGRFILLNLSVADLI
VALANLLGAAMSPRFRNVNDPSDINDSSSLLALSCKIDAFFGLFATDSSILWTIVLLSYLYITLHCFRPSRSCFKVSM
TICMIICWGLPLAISVWFISKNFFGFQPGFSPGFCTFTGDNDGKDIYRPIVGYEMFLYPSIIFLTTISIAFFCHAKCK
KKPVENYHYIEHHLNSIKAAERKLLFVPIVFIFIRIWGLITDVTTFYLNDNQIATLRNSKISPALIFMAGIGDSSQGF
VNGIFFCLLTPPVRQRLLLLLFKCGRICRCYCCSRYETLVNDAYITPTTSRSLTISYTNSTASRGGGVEGEGDGDNST
LSRSLGTISADISLYGSNEIN

→ Aq2.1 Gene: Aqu2.1.29931 Scaffold Contig13373: 75,230-76,927

MFLPHYDYDHGNNLSADQGDVSPTQDIGFIIALNISVGIVCLLSILGSTSIIFSYILYKELRTTGRFILLNLSVADLI
VALANLLGAAMSPRFRNVNDPSDINDSSSLLALSCKIDAFFGLFATDSSILWTIVLLSYLYITLHCFRPSRSCFKVSM
TICMIICWGLPLAISVWFISKNFFGFQPGFSPGFCTFTGDNDGKDIYRPIVGYEMFLYPSIIFLTTISIAFFCHAKCK
KKPVENYHYIEHHLNSIKAAERKLLFVPIVFIFIRIWGLITDVTTFYLNDNQIATLRNSKISPALIFMAGIGDSSQGF
VNGIFFCLLTPPVRQRLLLLLFKCGRICRCYCCSRYETLVNDAYITPTTSRSLTISYTNSTASRGGGVEGEGDGDNST
LSRSLGTISADISLYGSNEIN

Aq1 >PAC:15718483 ID:718483 scaffold:Aqu1:Contig13383:203940:204863:-1 gene:Aqu1.219955

MSLNSTDFVLMGDLNSTTYAAVLGIEGVIGIIVNVAVLLMTLYQRKSWNQPSTIFFTSLLLSNLIIALWYFMSSIAVG
AEEWIFGNTFKEKNASCLILGYMFWNGGMIIVATLAALSFDRFLFVVKPYLHKRFMRPRVALILIIGVWLLCSLINTT
PFYGFGVYRYQSRSGICSPSYEASGFFYVLYLVVVYSIIYFVIAFTSIWTFCFTRRFIQNQVEIVGSSVYESRRKRLF
GIFGYMLLSYVIALLPSYITGIISMFYQLPANVHLGTVVANGSFIISNPIIQSYFRPEIVTVIKNLWNKIRNT

→ Aq2.1 Gene: Aqu2.1.30476 Scaffold Contig13383: 203,940-204,863

MSLNSTDFVLMGDLNSTTYAAVLGIEGVIGIIVNVAVLLMTLYQRKSWNQPSTIFFTSLLLSNLIIALWYFMSSIAVG
AEEWIFGNTFKEKNASCLILGYMFWNGGMIIVATLAALSFDRFLFVVKPYLHKRFMRPRVALILIIGVWLLCSLINTT
PFYGFGVYRYQSRSGICSPSYEASGFFYVLYLVVVYSIIYFVIAFTSIWTFCFTRRFIQNQVEIVGSSVYESRRKRLF
GIFGYMLLSYVIALLPSYITGIISMFYQLPANVHLGTVVANGSFIISNPIIQSYFRPEIVTVIKNLWNKIRNT

Aq1 >PAC:15718485 ID:718485 scaffold:Aqu1:Contig13383:210830:211729:-1 gene:Aqu1.219957

MMDIMSFNSTDFVLTGDINSPTYAAVLGIEAIIGMIANVAVLLMTLYQKKSWNQPSTIFFTSLLLSNLIIALWYLMSS
IAVGAEEWIFGSTFEQRNATCLFNAYLLWSGRMIATMTLATVSFDRFLFVAKPYSYKRFMRPRVALILVIGVWVMSSL
INTIPFSGFGVYGYFSLNGICTLLYAYSGLYLIILFVVYAIIFVIIAVTSFWTFCFTRRFMQDQAEIAGGSVYQSRKK
RLFGIFGYMLLSYVIALLPSYIIGTLNMFYHLPAYVHLGVVVAYGSFITSNPIXXXXKSYHSIIF

→ Aq2.1 Gene: Aqu2.1.30478 Scaffold Contig13383: 210,830-211,830

MMDIMSFNSTDFVLTGDINSPTYAAVLGIEAIIGMIANVAVLLMTLYQKKSWNQPSTIFFTSLLLSNLIIALWYLMSS
IAVGAEEWIFGSTFEQRNATCLFNAYLLWSGRMIATMTLATVSFDRFLFVAKPYSYKRFMRPRVALILVIGVWVMSSL
INTIPFSGFGVYGYFSLNGICTLLYAYSGLYLIILFVVYAIIFVIIAVTSFWTFCFTRRFMQDQAEIAGGSVYQSRKK
RLFGIFGYMLLSYVIALLPSYIIGTLNMFYHLPAYVHLGVVVAYGSFITSNPIXXXXKSYHSIIF

Aq1 >PAC:15721359 ID:721359 scaffold:Aqu1:Contig13450:70950:73394:-1 gene:Aqu1.222831

MTSSELLSLSSTSTEISSPTCTSIAVTVTPTVTQTLVCIGETVSSTSIAVSTTTSTPTITSTSTTSTTPTPTEPPFDC
NTCYDSEECNWCLSNDCEISGCEKYTTCCDDFKKRRRRDTQANETIILASSSIMDSLDCSSVQVTITPSVTVSLTCFQ
ASTFPSPSPSPSITECENEPCSNLICSPVVQSAVTSGSVTSTVVIPSSSSSLPSPSSTLRSSSSSSTSDQNSAFVGTPTP
TCTSLNVAQGSFFEEMKDFVSCSPGNDPFNPCTDLIDNNLLRAGMWLVIILSIGGNIVVLSATLLYFISRYLKNHKKP

HLMYFLYINLAMADLFMGIYLLTIAVVDLDTIGDYSRHAIEWQTSAGCRFAGFCAIFSSLLSIYTLLVITVERVYTIK
FALQHKRFHKQTVTVSILVGWVLTITLCILPMVGLSSYERVGICLPFEARETADQAYIVLILVLTGIASFAIMFCYVL
LFYLVVCNNRSITKTLSGREELKLALRMSLLVMTDFACWAPIALFGLTAVFQKPLINVTDSKILMVFVFPLNSCLNPI
LYSFSTRKFRSIVFSAFDRCNVCKKCCAKKQIKQNSSNEEYSTGKSNSRRSSEDVTVNGPFLNQRQAHRRGTELSLIS
GFTTSSRRGSTLSGGSDEETPPMHSLIHTGRPERSSQSSLGSDNSYLSEQGESVINTSTSSLGSLKDRRFSLASLSGH
VSQLTALPEEKEEDLPCEKHRESTHSSSDSFNENIRLEYKPVLDDRKLPTNPSFIPLNEGTTTIYINECIDDDDDDDD
DAKGHQRSEEEQTIVTMYNDAEGIEIQETELTFT

→ Aq2.1 Gene: Aqu2.1.34596 Scaffold Contig13450: 70,759-75,424

MQFLLVAFFLVLSSCTANNKCHDRGFNAQRCSCSTASIECIPRGSSSSGILSYFPVATKKYDSILSIHIKGATFTHLN
KSHLEAYPKLHTLQIQDSSLSSISAEAFSSSTYLNKLVMTGNRLTEFPNLSGSKRLVKLELQYNKIRDISYDKVKQCP
LLTYIDLKHNEITTLRDGTFLDSCILTTLNVSENPLHTIGDNFFEGVSMTNLDLSYTDISSFPTTGLRRYLKYLNLDR
CKNLKTFPPARTDYDPYLCTDLTFPLLKEVKFEYHALCCQFRTQKSLKEVTFSNNRCPSRTRSFLFRSADKVEKRNAN
MPSPSTTLLHTSLCWSNGTIIPSTTPSIVMTSSELLSLSSTSTEISSPTCTSIAVTVTPTVTQTLVCIGETVSSTSIA
VSTTTSTPTITSTSTTSTTPTPTEPPFDCNTCYDSEECNWCLSNDCEISGCEKYTTCCDDFKKRRRRDTQANETIILA
SSSIMDSLDCSSVQVTITPSVTVSLTCFQASTFPSPSPSITECENEPCSNLICSPVVQSAVTSGSVTSTVVIPSSSSS
LPSPSSTLRSSSSSSSTSDQNSAFVGTPTPTCTSLNVAQGSFFEEMKDFVSCSPGNDPFNPCTDLIDNNLLRAGMWLVI
ILSIGGNIVVLSATLLYFISRYLKNHKKPHLMYFLYINLAMADLFMGIYLLTIAVVDLDTIGDYSRHAIEWQTSAGCR
FAGFCAIFSSLLSIYTLLVITVERVYTIKFALQHKRFHKQTVTVSILVGWVLTITLCILPMVGLSSYERVGICLPFEA
RETADQAYIVLILVLTGIASFAIMFCYVLLFYLVVCNNRSITKTLSGREELKLALRMSLLVMTDFACWAPIALFGLTA
VFQKPLINVTDSKILMVFVFPLNSCLNPILYSFSTRKFRSIVFSAFDRCNVCKKCCAKKQIKQNSSNEEYSTGKSNSR
RSSEDVTVNGPFLNQRQAHRRGTELSLISGFTTSSRRGSTLSGGSDEETPPMHSLIHTGRPERSSQSSLGSDNSYLSE
QGESVINTSTSSLGSLKDRRFSLASLSGHVSQLTALPEEKEEDLPCEKHRESTHSSSDSFNENIRLEYKPVLDDRKLP
TNPSFIPLNEGTTTIYINECIDDDDDDDDDAKGHQRSEEEQTIVTMYNDAEGIEIQETELTFT

Aq1 >PAC:15721762 ID:721762 scaffold:Aqu1:Contig13456:263938:287969:-1
gene:Aqu1.223234

MASTRYLPGATSVEIPLRDTDEVIELDIDQLPEGDEVITILRDERAPLHTWVTLALHYYKQDKWSEFEKLLQTSRTDA
NISYDDHEKDQMMALDSLAAHYVQKAKKERDKETKKEYFSKATLLYSTADKIMMYDSRHLLGRAYLCLLEGDKMSQSE
AQFNFVLQQSPDNLPALLGKACVSFNRKDYKGALVCYKKALRSNPNCSGTVRLGMGHCFMKLGNIDKAQCVGALVGLA
ILELNNQQHDSIKRGVELLSKAYTIDSVNPMVLNHLANHFFFKKDYVKVQHLALHAFHGTEVETMRSESCYQLARAFH
VQGDYDQAFQYYYQATQFAAPGFVLPHFGLGQMYLARQDSENAAQCFEKVLASQPGNYETLKILGSLYANSPSSEKRA
TAVTHLKKVTEEFPDDVEAWIELGGILEATDTEGSLKAYEKASQLLTETVGTDIPPEILNNIGCLHFKLGQYNEAQSH
YDQSLDRCTQECMQDEEYYNSLMVTVRYNMARLHEALCEFEKAETLYKEILKEHPRYIDCYLRLGCIARDRQQIYEAS
DWFKEALQKNQDHADAWVLMGNLHLAKQEWGPGQKKFERILQNPKTKGDTYSLLSLGNVWLASIHQPHRDKTKDKRHL
DRALSYYKDVLHKDSHNLYAANGIGAVLGHKGFYREARDVFAQVREATAELPDVWLNLAHVYIEQKQYISAIQMYENC
LGKFYNFHNTEVLLYLARAYFKAGRILDCKTTLIKARHIAPHDSLLLFNLALVQRWSASSTLKNLQSTLADVLSAVRE
LEMAQRNFVFLSREGDRLKFDLQFASHEAKRCADLLSQAQHHVARARKSEDEERELREKQEKEMETLRQKQIEQEEKL
AKERERQLQLQEQARQEFIQKTQNLLHFTREASPPPKPSRKKRGTADVQSDDPENEASASGPQRKRRRRNNEEDTGDT
AASGGKGRKKKRFISQAFVSSSEDESGGEEKKTEETAVEEREGESHEGEEEVGERSLQSEDSSEESTSGSESESGSGS
NDEVRQEEEGKEEDEEEAKGEEPKEGSSPEPQEDTTAEQEVEEKVTQHNVSNRRRIESDDDETLAIDCRRLELTAVPE
FLQTFSNYSTFKQISMYLDYNNISGIGTNDLSLMKNLTRLTITHNQLSYIEDNAFSYQDFFNILDLSFNLLTSESFAN
DSFNSTGSRVATLKITDNLLTHPPRALAKKNRHMSMSTLFLDNNPMGPVLKSDAFEGYAQDIRELFLNNNDFNEIDVK
FLEGAHISDKLYLRNAGIREIRPRTFWKHTSLKHLYLDNNDLSRQESRNSFVKMDLKTLNLKGNFITRIFNETFRDLK
SIRELILDSNKINRIDAQPFRKMTLLRKLYLGNNEIDDSNPYFLGDLQSLTSLDISRNRLTEFPDVSGLPILTTVLAS
YNNIKELKEATFNTSEYLKKLQLSDNRNLEVIDRNAFKRNNQIEMVDVSFSGLKRLPSFETFPQLYELRLEHSKLTEL
PKDLCTIAPNLRLLYAYDNYLESIPDLSNCTKLIHIELYYNRIKAIPEFAFKGLSDLQTLKLYGNRITTVHRNAFRGL
NRLYELDLSHNQITYLPHGLFFNLTNLRKLELQFNAITNLPDNIFSDLNHLELLYINDNAIRTVGSVLFHGNMTWLQN
LSVSNNPEMDSFPIPTDGFPFLHSLGMSNLPLIFNVPTVINIPRIQTIDFTYSYHCCLFKDYAPPYRALTLEESENTG
DPDVLFTFQNTPAEEVTLAPEVSDTLLFHGSDPFTHDNAGDISPQQFIRELLEYQRRFNVTLVSTQNGQVIVVDNSNH
SIVARLKDEESRYLRTILPSFFVNREVYCTPPPSSLTPCDNLLDPQPLPVLVWVIWFTAVIANLAVLFVMIVSKEKLE
VPHFFICNLAFADFLLGVYLAFLGAVDIRTRGKGFYKSALTWQTGPGCQTAGFIAIFSAELSVFILTFLTLERLHTIA
YSFKSGRLRLRNATIIVFICWILAGILAALPLFDVNTYSEVAVCLPFRLSNIRDKLFIALILTVNLTAFFIILSSYLH
ILRLFCRSRASCTEQGNKREKIVISFKMGMLVLTNLICWLPLAVVGYAAIVDQHIINFTVAKFFIILIFPINACLNPF
IYSIFTKQFLSRVRGLCKRKDTMQHVSNNNSFYRLSFRRNSIASAASDTCSSTLRTNSPRFDIDLDMLSRRQSRRSFS
VQLASPPLPQPIVGLASPAPYMGRRYSSPAIFGMESGLPEAQGLEGRGEIPRSHSKCLSIVQEESDESDTEEAAASHS
PRRRRRSSDPELLNSALPGGLKDLLNARSNKVSDERRDSGLVPSFEGNSPLHYQSVSPPAQSSTLIRTSGEGHSRDDA
TSTNYRIPSLSSPAAASRVHFKEYIPRRSSPLSTHSIRIINPHTAKQSKHNVKESQV

→ Aq2.1 Gene: Aqu2.1.35178 Scaffold Contig13456: 263,896-275,092

MCSSYSQRWLSLILSLLCLFSLIKTSHAYLPFYCHGCSCVVTPPSALAIDCRRLELTAVPEFLQTFSNYSTFKQISMY
LDYNNISGIGTNDLSLMKNLTRLTITHNQLSYIEDNAFSYQDFFNILDLSFNLLTSESFANDSFNSTGSRVATLKITD
NLLTHPPRALAKKNRHMSMSTLFLDNNPMGPVLKSDAFEGYAQDIRELRLSGCQLKKIEPKAFHYEIFPALKTLFLNN
NDFNEIDVKFLEGAHISDKLYLRNAGIREIRPRTFWKHTSLKHLYLDNNDLSRQESRNSFVKMDLKTLSLTNCSLTRL
RRGFLRLTATSINLKGNFITRIFNETFRDLKSIRELILDSNKINRIDAQPFRKMTLLRKLYLGNNEIDDSNPYFLGDL
QSLTSLDISRNRLTEFPDVSGLPILTTVLASYNNIKELKEATFNTSEYLKKLQLSDNRNLEVIDRNAFKRNNQIEMVD
VSFSGLKRLPSFETFPQLYELRLEHSKLTELPKDLCTIAPNLRLLYAYDNYLESIPDLSNCTKLIHIELYYNRIKAIP
EFAFKGLSDLQTLKLYGNRITTVHRNAFRGLNRLYELDLSHNQITYLPHGLFFNLTNLRKLELQFNAITNLPDNIFSD
LNHLELLYINDNAIRTVGSVLFHGNMTWLQNLSVSNNPEMDSFPIPTDGFPFLHSLGMSNLPLIFNVPTVINIPRIQT
IDFTYSYHCCLFKDYAPPYRALTLEESENTGDPDVLFTFQNTPAEEVTLAPEVSDTLLFHGSDPFTHDNAGDISPQQF
IRELLEYQRRFNVTLVSTQNGQVIVVDNSNHSIVARLKDEESRYLRTILPSFFVNREVYCTPPPSSLTPCDNLLDPQP
LPVLVWVIWFTAVIANLAVLFVMIVSKEKLEVPHFFICNLAFADFLLGVYLAFLGAVDIRTRGKGFYKSALTWQTGPG
CQTAGFIAIFSAELSVFILTFLTLERLHTIAYSFKSGRLRLRNATIIVFICWILAGILAALPLFDVNTYSEVAVCLPF
RLSNIRDKLFIALILTVNLTAFFIILSSYLHILRLFCRSRASCTEQGNKREKIVISFKMGMLVLTNLICWLPLAVVGY
AAIVDQHIINFTVAKFFIILIFPINACLNPFIYSIFTKQFLSRVRGLCKRKDTMQHVSNNNSFYRLSFRRNSIASAAS
DTCSSTLRTNSPRFDIDLDMLSRRQSRRSFSVQLASPPLPQPIVGLASPAPYMGRRYSSPAIFGMESGLPEAQGLEGR
GEIPRSHSKCLSIVQEESDESDTEEAAASHSPRRRRRSSDPELLNSALPGGLKDLLNARSNKVSDERRDSGLVPSFEG
NSPLHYQSVSPPAQSSTLIRTSGEGHSRDDATSTNYRIPSLSSPAAASRVHFKEYIPRRSSPLSTHSIRIINPHTAKQ
SKHNVKESQV

AND
→ Aq2.1 Gene: Aqu2.1.35181 Scaffold Contig13456: 277,472-287,738

MASTRYLPGATSVEIPLRDTDEVIELDIDQLPEGDEVITILRDERAPLHTWVTLALHYYKQDKWSEFEKLLQTSRTDA
NISYDDHEKDQMMALDSLAAHYVQKAKKERDKETKKEYFSKATLLYSTADKIMMYDSRHLLGRAYLCLLEGDKMSQSE
AQFNFVLQQSPDNLPALLGKACVSFNRKDYKGALVCYKKALRSNPNCSGTVRLGMGHCFMKLGNIDKAQCVGALVGLA
ILELNNQQHDSIKRGVELLSKAYTIDSVNPMVLNHLANHFFFKKDYVKVQHLALHAFHGTEVETMRSESCYQLARAFH
VQGDYDQAFQYYYQATQFAAPGFVLPHFGLGQMYLARQDSENAAQCFEKVLASQPGNYETLKILGSLYANSPSSEKRA
TAVTHLKKVTEEFPDDVEAWIELGGILEATDTEGSLKAYEKASQLLTETVGTDIPPEILNNIGCLHFKLGQYNEAQSH
YDQSLDRCTQECMQDEEYYNSLMVTVRYNMARLHEALCEFEKAETLYKEILKEHPRYIDCYLRLGCIARDRQQIYEAS
DWFKEALQKNQDHADAWVLMGNLHLAKQEWGPGQKKFERILQNPKTKGDTYSLLSLGNVWLASIHQPHRDKTKDKRHL
DRALSYYKDVLHKDSHNLYAANGIGAVLGHKGFYREARDVFAQVREATAELPDVWLNLAHVYIEQKQYISAIQMYENC
LGKFYNFHNTEVLLYLARAYFKAGRILDCKTTLIKARHIAPHDSLLLFNLALVQRWSASSTLKNLQSTLADVLSAVRE
LEMAQRNFVFLSREGDRLKFDLQFASHEAKRCADLLSQAQHHVARARKSEDEERELREKQEKEMETLRQKQIEQEEKL
AKERERQLQLQEQARQEFIQKTQNLLHFTREASPPPKPSRKKRGTADVQSDDPENEASASGPQRKRRRRKKHSESSRR
KEERGEKRRQRKRESGEKRRKKQKSNEEDTGDTAASGGKGRKKKRFISQAFVSSSEDESGGEEKKTEETAVEEREGES
HEGEEEVGERSLQSEDSSEESTSGSESESGSGSNDEVRQEEEGKEEDEEEAKGEEPKEGSSPEPQEDTTAEQEVEEKV
TQHNVSNRRRIESDDDESD

Aq1 >PAC:15722199 ID:722199 scaffold:Aqu1:Contig13465:46025:47249:-1
gene:Aqu1.223671

MSNNSTDYSYPCGLPPDFPPSFGIPPAINYIQAGIALVTGIIGLLLNIFILFIIIKYRSLHQRLMFIAIQITIVEIAY
SLLVPPAIVVSGIARDWLLGEAMCNILGIVNDGFAYFRFMMTFILTLDRFVSVFLPFFYEKHSKRIIYSLLSLVYFTT
FFRVLLPVKGIMGCYIYAYRLRRATRRIVPENLSAESQRGNETKILKESFQFFTNITHGAFFAVVMLVGRTSFNSIPV
VDAIAIMKDKEFREAAKKLILSIRERLDEFHSLSVEEWKPKH

→ Aq2.1 Gene: Aqu2.1.35938 Scaffold Contig13465: 46,025-47,253

MSNNSTDYSYPCGLPPDFPPSFGIPPAINYIQAGIALVTGIIGLLLNIFILFIIIKYRSLHQRLMFIAIQITIVEIAY
SLLVPPAIVVSGIARDWLLGEAMCNILGIVNDGFAYFRFMMTFILTLDRFVSVFLPFFYEKHSKRIIYSLLSLVYFTT
FFRVLLPVKGIMGCYIYAYRLRRATRRIVPENLSAESQRGNETKILKESFQFFTNITHGAFFAVVMLVGRTSFNSIPV
VDAIAIMKDKEFREAAKKLILSIRERLDEFHSLSVEEWKPKH

Aq1 >PAC:15722200 ID:722200 scaffold:Aqu1:Contig13465:47402:48655:1
gene:Aqu1.223672

MNNSSLESSYACGLPEDFPPSLNIPPAINYIQAGIALVTGIIGLLLNIFILFIIIKYRSLRQRLMYIAIQISIVEIAY
SLLVPPAIFISGIAREWLLGEAMCNILGIVNDGFAYFRFMMTFILTLDRFISIFAPFFYERNSKKILFGLHGLVYCTT
FFRVLLPINGIMSCYVYVSTNKICTAFSGCSLGCYYFVFVSIILIIVVGALLPLSMYVILFIKAYRVKKATKKMVPAP
STPSEEMKSCTSQKGSVVYPELASAPSAFDDDLTDQKTKRQIRPSVTSSKSRHLSLSAENKKSLQVTITMFILLLSVI

GCTSPAFVLYMVQFLSLHDQKTFFILIMLLGRTSFNSIPVVDAIAIMRDQQFRKSIKLCFRR

→ Aq2.1 Gene: Aqu2.1.35939 Scaffold Contig13465: 47,391-48,654

MNNSSLESSYACGLPEDFPPSLNIPPAINYIQAGIALVTGIIGLLLNIFILFIIIKYRSLRQRLMYIAIQISIVEIAY
SLLVPPAIFISGIAREWLLGEAMCNILGIVNDGFAYFRFMMTFILTLDRFISIFAPFFYERNSKKILFGLHGLVYCTT
FFRVLLPINGIMSCYVYVSTNKICTAFSGCSLGCYYFVFVSIILIIVVGALLPLSMYVILFIKAYRVKKATKKMVPAP
STPSEEMKSCTSQKGSVVYPELASAPSAFDDDLTDQKTKRQIRPSVTSSKSRHLSLSAENKKSLQVTITMFILLLSVI
GCTSPAFVLYMVQFLSLHDQKTFFILIMLLGRTSFNSIPVVDAIAIMRDQQFRKSIKLCFRR

Aq1 >PAC:15722205 ID:722205 scaffold:Aqu1:Contig13465:58133:59373:1
gene:Aqu1.223677

MDANYTCGLPPDFPPSLNIPPAISYVQASIALVTGIVGLLLNAFILIIIIKYRSLHQRLMYIAIQIIVVEIAYSLLVP
PAIFVSGIARDWLLGEAMCNILGIINDGFAYFRFMMTFILTLDRFVSVFFPFVYERNSKKILIILLIMVYFTTFLRVI
LPLKGVMGCYTYVPTNKICTAYSDCSTGCYYFVLASVVTIVLIGAIIPFCMYMILFYKAYKVRKISSQAVTTHSPSPS
FSSIATVQVNMSTIKGSLPRGRESSICEHDLEKSRKSISGDENFQVTLTMFILLMSVIGCTSPAFVLYIVQFISLHNN
SAFFIVIMLLGRTSFNSIPIFDAIAIMRDKQFRSSAKTFLGTLRKCI

→ Aq2.1 Gene: Aqu2.1.35944 Scaffold Contig13465: 58,045-59,410

MDANYTCGLPPDFPPSLNIPPAISYVQASIALVTGIVGLLLNAFILIIIIKYRSLHQRLMYIAIQIIVVEIAYSLLVP
PAIFVSGIARDWLLGEAMCNILGIINDGFAYFRFMMTFILTLDRFVSVFFPFVYERNSKKILIILLIMVYFTTFLRVI
LPLKGVMGCYTYVPTNKICTAYSDCSTGCYYFVLASVVTIVLIGAIIPFCMYMILFYKAYKVRKISSQAVTTHSPSPS
FSSIATVQVNMSTIKGSLPRGRESSICEHDLEKSRKSISGDENFQVTLTMFILLMSVIGCTSPAFVLYIVQFISLHNN
SAFFIVIMLLGRTSFNSIPIFDAIAIMRDKQFRSSAKTFLGTLRKCI

Aq1 >PAC:15722301 ID:722301 scaffold:Aqu1:Contig13466:127368:127973:1
gene:Aqu1.223773

MESNSTFSEEFSGPAVAAVFTVEMILALIANGVVLSITLYQRKSLKQSSTIFFTSLILAHLVLNLLYLPLFIIGLAAG
EWIFGSTDEEKRGTCYFGAFIFWWMVLVISLTLAAISFDRFLFIVKPHLHKRFMRPWVALTLTIAIWILSAVLSIVHL
FGFRLYIDICLSSTADIGFVVYIILLSAIVLGIIFVTSFWTFFFH

→ Aq2.1 Gene: Aqu2.1.36056 Scaffold Contig13466: 127,368-127,973

MESNSTFSEEFSGPAVAAVFTVEMILALIANGVVLSITLYQRKSLKQSSTIFFTSLILAHLVLNLLYLPLFIIGLAAG
EWIFGSTDEEKRGTCYFGAFIFWWMVLVISLTLAAISFDRFLFIVKPHLHKRFMRPWVALTLTIAIWILSAVLSIVHL
FGFRLYIDICLSSTADIGFVVYIILLSAIVLGIIFVTSFWTFFFH

Aq1 >PAC:15722303 ID:722303 scaffold:Aqu1:Contig13466:143707:144618:1
gene:Aqu1.223775

MDGNLTFTGEFSGPAVAAVLTVEMILALIANGVVLSITLCRRKSLKQSSTIFFTSLILAHLVLNLLYLPFRIIALAAG
EWIFGSTDEEKRGTCRFFDVFIYIWMVLVISMTLAAISFDRFLFIVKPHLHKRFMRPWVALTLTVAIWILSAVLSSIY
SFGFQLKFDSHYNCSVTISISLGVSAIILAFIFNGIIFITSLWTFCFTRRFFKDQSVIAGESVYASKEKRLFGIFGCM
LLVYAICFVPGVLYFLLLLFINVPLWLNIVVIVCFLFITVASPLIQSYFRPDIKSVLVLYCPFHFCVCC

→ Aq2.1 Gene: Aqu2.1.36058 Scaffold Contig13466: 141,230-146,989

MFSVNTFIRICVILETLGSTDPHHCYLDTVCPIVLGIIFITSLWTFCFTRRFFKDQSVIAGESVYVSKKKRLFGIFGS
TLLIYGICFRPAVAPFEMTLALIANGVVLSITLYQRKSLNQQFTGTSCIESAVPFFDYHWTSCWFFKDQSVIAGEIAN
PVAQFYFRPETKSVLVTHCPLHIFILLVLSSAKLMYLMSMLNVSNVIIDQQYSREPSMDGNLTFTGEFSGPAVAAVLT
VEMILALIANGVVLSITLCRRKSLKQSSTIFFTSLILAHLVLNLLYLPFRIIALAAGEWIFGSTDEEKRGTCRFFDVF
IYIWMVLVISMTLAAISFDRFLFIVKPHLHKRFMRPWVALTLTVAIWILSAVLSSIYSFGFQLKFDSHYNCSVTISIS
LGVSAIILAFIFNGIIFITSLWTFCFTRRFFKDQSVIAGESVYASKEKRLFGIFGCMLLVYAICFVPGVLYFLLLLFI
NVPLWLNIVFTSYMESNSTFSDQFSGPAVAAVLIVEVILALIANGVVLSVTLYQRKSLKQPSTIFFTSLILAHLVFNL
LFVPFIIIGLAASEWISDSTDEEKRGTCYFFLYIYWWILPVISITLAAISFDRFLFIVKPHLHKQFMRPWVALILTIA
IWILSAVLSIVHLFGLRLYTDICLSSSADIGFVVYFILLLAIVLGIIFILSLWTFCFTRRFFKDQSVIAGESVYASKK
KRLFGIFGCMLLIHGICFIPGVLYFLLLLFIDVPLWLNFAAIICFHFVTVANPVMQSYFRPEIKSVLFRRPLHICVCC

Aq1 >PAC:15722304 ID:722304 scaffold:Aqu1:Contig13466:158017:158931:1
gene:Aqu1.223776

MNFNMESNSTFSDQFSGPALAGILIVEMILALIANGVVLSITLYQRKSLKQPSTMFFTSLILAHLVMNLLNLPFIIIA
LAAGEWIFGSTDEEKRGTCYFKDFIFWWMVVVMLTTLAAISFDRFLFIVKPHLHKQFMRPWVALTLTIAIWILSAVLS
IVHLFGFHSYTDICLPSTTGIGFSVYAILLSAIVLGIIFVTSLWTFCFTRRFFKDQSVIAGESVYISKKKRLFGIFGS
MLLIYGICFVPAVLYFLLRLFIDVPIWFIIVAIICFHFITVINPVMQSYFRPEIKSVIFTCCPLHICVCC

→ Aq2.1 Gene: Aqu2.1.36059 Scaffold Contig13466: 158,017-158,931

MNFNMESNSTFSDQFSGPALAGILIVEMILALIANGVVLSITLYQRKSLKQPSTMFFTSLILAHLVMNLLNLPFIIIA
LAAGEWIFGSTDEEKRGTCYFKDFIFWWMVVVMLTTLAAISFDRFLFIVKPHLHKQFMRPWVALTLTIAIWILSAVLS
IVHLFGFHSYTDICLPSTTGIGFSVYAILLSAIVLGIIFVTSLWTFCFTRRFFKDQSVIAGESVYISKKKRLFGIFGS
MLLIYGICFVPAVLYFLLRLFIDVPIWFIIVAIICFHFITVINPVMQSYFRPEIKSVIFTCCPLHICVCC

Aq1 >PAC:15722305 ID:722305 scaffold:Aqu1:Contig13466:174002:175763:-1
gene:Aqu1.223777

MVYSRETEGMESNYTYTSDFSGPAVAAVLTVEMILALIANGVVLSITLYQRKSLKQPSTIFFTSLILAHLVLNLLYFP
FFIIAFAAEKWIFGSTDEEKKGTCYFVDFTFWWILLVISITLAAISFDRFLFIVKPHLHKRFMRAWVALTLTIAIWIL
SAVLITILGIIFVTSLWTFCFTRRFFKDQSVIAGESVYASKEKRLFGIFGSMLLIITGCIKCILNLIA

→ Aq2.1 Gene: Aqu2.1.36060 Scaffold Contig13466: 174,194-175,096

MESNYTYTSDFSGPAVAAVLTVEMILALIANGVVLSITLYQRKSLKQPSTIFFTSLILAHLVLNLLYFPFFIIAFAAE
KWIFGSTDEEKKGTCYFVDFTFWWILLVISITLAAISFDRFLFIVKPHLHKRFMRAWVALTLTIAIWILSAVLSSVHV
FGFHLYVSICISYSESIGFGVYYFLLSVTILGIIFVTSLWTFCFTRRFFKDQSVIAGESVYASKEKRLFGIFGSMLLM
YGLCYIPGVLYFLFSLFFDVPLWFNTVATLCFLFITVGSPVIQSYFRPEIRSVLVSRFPLHFCVHC

Aq1 >PAC:15722306 ID:722306 scaffold:Aqu1:Contig13466:176289:177188:-1
gene:Aqu1.223778

MERNFTFTGNFSGPAVAAVLTVEMILALIANGVVLSITLYQKKSWKQSSTIFFTSLILAHLVLNLLYLPFIIIGLAAE
EWIFGSTDEEKRGTCYFNDFIYWWMVIVISITLAAISFDRFLFIVKPHLHKQFMRPWVALILTIAIWILSAVLSSVHV
FGFHLYIDICFSYSDNIGFRVYFVLLSAIVLMIIFITSLWTFCFTRRFFKDQSVIAGESVYASKKKRLFGIFGAMLLI
YGICFVPGVLYFLLLLFIDVPIWFIIVAIICFLFITIASPVIQSYFRPEIKSVLVSCFLLCNCKC

→ Aq2.1 Gene: Aqu2.1.36061 Scaffold Contig13466: 176,141-177,321

MERNFTFTGNFSGPAVAAVLTVEMILALIANGVVLSITLYQKKSWKQSSTIFFTSLILAHLVLNLLYLPFIIIGLAAE
EWIFGSTDEEKRGTCYFNDFIYWWMVIVISITLAAISFDRFLFIVKPHLHKQFMRPWVALILTIAIWILSAVLSSVHV
FGFHLYIDICFSYSDNIGFRVYFVLLSAIVLMIIFITSLWTFCFTRRFFKDQSVIAGESVYASKKKRLFGIFGAMLLI
YGICFVPGVLYFLLLLFIDVPIWFIIVAIICFLFITIASPVIQSYFRPEIKSVLVSCFLLCNCKC

Aq1 >PAC:15722307 ID:722307 scaffold:Aqu1:Contig13466:179848:181571:1
gene:Aqu1.223779

MEDNYTLTDQFSGPAVAAVLTVEMILALIANGVVLSITLYQRKSLKQPSTVFFTSLILAHLVLNLLYLPFYIISLSAG
EWIFNGSDEEKRGTCYFALYIFWWMVLVISTTLAAISFDRFLFIVKPHLHNRYMKPWVALTLTIAIWILSAVYTTFYI
FGFQFDHHQFDSQYCSLPSSSSRGFFGLFAALIILGIIFVFSIWTFCFTRRFFRDQSVIAGESAYASKKKRLFGIFGS
MLLIYGICLVPAVVYSLLYLFIDVPIWFIIIGTISFLFFTIANPVIQSYFRPEIMGVLSWLDNKHTVFGRFFMDVAQK
ISEVKTDPRSNNPYDEIKVI

→ Aq2.1 Gene: Aqu2.1.36062 Scaffold Contig13466: 179,848-181,688

MEDNYTLTDQFSGPAVAAVLTVEMILALIANGVVLSITLYQRKSLKQPSTVFFTSLILAHLVLNLLYLPFYIISLSAG
EWIFNGSDEEKRGTCYFALYIFWWMVLVISTTLAAISFDRFLFIVKPHLHNRYMKPWVALTLTIAIWILSAVYTTFYI

FGFQFDHHQFDSQYCSLPSSSSRGFFGLFAALIILGIIFVFSIWTFCFTRRFFRDQSVIAGESAYASKKKRLFGIFGS
MLLIYGICLVPAVVYSLLYLFIDVPIWFIIIGTISFLFFTIANPVIQSYFRPEIMGVLSWLDNKHTVFGRFFMDVAQK
ISEVKTDPRSNNPYDEIKVI

Aq1 >PAC:15722410 ID:722410 scaffold:Aqu1:Contig13467:295897:296859:1
gene:Aqu1.223882

MSFNDSFIAMGTINGPAVATVLAIEGLIGFIANIIVLGITIYRKKSWKQSSTIIFTSIILAHLIMIIMFLPLFVIAIG
AEEWIFGSDDMQKRQMCLFLNFVEWTMKLLIQIMIAEVSFDRFLFLYKPHHHKKYMRWWVTLTLTTVLWIVAIILNVL
PLFVDDNDFSYIFTYRPCIPRRQVSSYYFLVDLASYILIYVFIFITSVWTLCFTYRFIHNLSEVAGESVYASKKKRLF
GIFGSMLLAYFIGFAPFTIAIIIRSFYGVPIEVFATTEVTFQMTHIATPLIQSYFRPEITNTLISFKAFLRQKIHSAR
YDVRNPEI

→ Aq2.1 Gene: Aqu2.1.36175 Scaffold Contig13467: 295,897-296,988

MSFNDSFIAMGTINGPAVATVLAIEGLIGFIANIIVLGITIYRKKSWKQSSTIIFTSIILAHLIMIIMFLPLFVIAIG
AEEWIFGSDDMQKRQMCLFLNFVEWTMKLLIQIMIAEVSFDRFLFLYKPHHHKKYMRWWVTLTLTTVLWIVAIILNVL
PLFVDDNDFSYIFTYRPCIPRRQVSSYYFLVDLASYILIYVFIFITSVWTLCFTYRFIHNLSEVAGESVYASKKKRLF
GIFGSMLLAYFIGFAPFTIAIIIRSFYGVPIEVFATTEVTFQMTHIATPLIQSYFRPEITNTLISFKAFLRQKIHSAR
YDVRNPEI

Aq1 >PAC:15722868 ID:722868 scaffold:Aqu1:Contig13474:41881:42879:-1
gene:Aqu1.224340

MERNYTFTGDFSPEAVAAVLSIEMILALIANGVVLVITIYQRKSWKQSSTIFFTSLILAHLVLTLYLPFTISALAARE
WIIGSTDEEKQGTCGFTGFVNLLSIYVMLMTLCLVSIDRFLFIVKPHLHKRFMTPRIALVLVIIVWSFDTAFFSSGFI
DGSGVEFRYINYLGVCYAFTTSPVMAIFRFFFASLIFIIIIITSVWTFCFTRKFINNQSMIVGEGVYASKKKRLFGIF
GSMMLVYGIFFTPSIFFSALLAIVDFPGVLMITAVIFFFLVITLSPVIQAYFRPEINSVIANIVCCKILKNKSYPLAI
SSTCNSSSAVLDIRVSSLDV

→ Aq2.1 Gene: Aqu2.1.36740 Scaffold Contig13474: 41,881-42,879

MERNYTFTGDFSPEAVAAVLSIEMILALIANGVVLVITIYQRKSWKQSSTIFFTSLILAHLVLTLYLPFTISALAARE
WIIGSTDEEKQGTCGFTGFVNLLSIYVMLMTLCLVSIDRFLFIVKPHLHKRFMTPRIALVLVIIVWSFDTAFFSSGFI
DGSGVEFRYINYLGVCYAFTTSPVMAIFRFFFASLIFIIIIITSVWTFCFTRKFINNQSMIVGEGVYASKKKRLFGIF
GSMMLVYGIFFTPSIFFSALLAIVDFPGVLMITAVIFFFLVITLSPVIQAYFRPEINSVIANIVCCKILKNKSYPLAI
SSTCNSSSAVLDIRVSSLDV

Aq1 >PAC:15723144 ID:723144 scaffold:Aqu1:Contig13478:344429:345397:1
gene:Aqu1.224616

MNYSSDEVNGPAVAAILIIEMALGLVANSIVLVITFTQRQSWKQPSTIFFTSLILAHLVLLLLYLPFFIVSSIAGEWV
FGKTLQEKRDSCSVAAYIIWYSALVITLTLSVISFDRFLFIVKPNQYKAFMKQRVALCLTITIWLLAALLNSTPLYGL
GEFGYRSYGTCVPLWEDNRGYVFYMLIVFALAVSVIIITSVWTMCFTRNFLSHQSEIAGNSVYTSKKKRLFGIFGTML
IVYVICLSPGFIVGFLSQFVDMPEEFYLTMIFCFVAITFVNPLVQSYFRVDTVMCIKCIHKKIERNKSSTCYINDDTV
STRFKSKSEP

→ Aq2.1 Gene: Aqu2.1.37171 Scaffold Contig13478: 344,429-345,397

MNYSSDEVNGPAVAAILIIEMALGLVANSIVLVITFTQRQSWKQPSTIFFTSLILAHLVLLLLYLPFFIVSSIAGEWV
FGKTLQEKRDSCSVAAYIIWYSALVITLTLSVISFDRFLFIVKPNQYKAFMKQRVALCLTITIWLLAALLNSTPLYGL
GEFGYRSYGTCVPLWEDNRGYVFYMLIVFALAVSVIIITSVWTMCFTRNFLSHQSEIAGNSVYTSKKKRLFGIFGTML
IVYVICLSPGFIVGFLSQFVDMPEEFYLTMIFCFVAITFVNPLVQSYFRVDTVMCIKCIHKKIERNKSSTCYINDDTV
STRFKSKSEP

Aq1 >PAC:15723376 ID:723376 scaffold:Aqu1:Contig13481:356027:357182:-1
gene:Aqu1.224848

MDYNFTTTGNINGPVLAAVFAVEAVVGFIANIIVLSITLHQRKSFKQPSTIFFTSLILSNLLNVLVYLPMTTVATGAE
EWILGSTFEQRRATCVFAGIISWINVYVITGVLAAISFDKCLFITKPYFYKRFMKPWVALAITVTLWIIVALMFTLPS
LGFGEYDYLFDYGPCYIAFRRNRGYVIFCISLLLSFIIIIIITSIWTFCFIRRFIRDHSAMEGDSVYNTQRLKLFGIF
GSMLLAYGTCYGPVIIIGFISFAIQIPEELDAATIVAFHAIPTMTALIQSYFRPDITKPLASFKNAICSKLNAAHSSS
N

→ Aq2.1 Gene: Aqu2.1.37502 Scaffold Contig13481: 356,157-357,098

MDYNFTTTGNINGPVLAAVFAVEAVVGFIANIIVLSITLHQRKSFKQPSTIFFTSLILSNLLNVLVYLPMTTVATGAE
EWILGSTFEQRRATCVFAGIISWINVYVITGVLAAISFDKCLFITKPYFYKRFMKPWVALAITVTLWIIVALMFTLPS
LGFGEYDYLFDYGPCYIAFRRNRGYVIFCISLLLSFIIIIIITSIWTFCFIRRFIRDHSAMEGDSVYNTQRLKLFGIF
GSMLLAYGTCYGPVIIIGFISFAIQIPEELDAATIVAFHAIPTMTALIQSYFRPDITKPLASFKNAICSKLNAAHSSS
N

Aq1 >PAC:15723377 ID:723377 scaffold:Aqu1:Contig13481:357495:358439:-1
gene:Aqu1.224849

MDYNFTATGSINGPVLAAVFAVEAVVGFIANIIVLSITLHQRKSFKQPSTIFFTSLILSNLLDALIYLPMTAIATGAE
EWILGSTFEQRRATCVFAGIISWINVYIITGVLAAISFDKCLFITKPYFYKRFMKPWVALAIIVVLSIIVTLMFTLPS
LGFGEYDYLFDYGPCYVAFRRNRGYVIFCISLLLSFIIIIMVTSIWTFCFTHRFIRDHSEKGGDSVYNTQRLKLFGIF
GSMLLAYGTCYGSVIVTAFISYGIQIPEEVDAATIVAFHANPIVTALIQSYFRPDITKPLKSFKNAICSKLKKMACSF
SN

→ Aq2.1 Gene: Aqu2.1.37503 Scaffold Contig13481: 357,410-358,439

DYNFTATGSINGPVLAAVFAVEAVVGFIANIIVLSITLHQRKSFKQPSTIFFTSLILSNLLDALIYLPMTAIATGAEE
WILGSTFEQRRATCVFAGIISWINVYIITGVLAAISFDKCLFITKPYFYKRFMKPWVALAIIVVLSIIVTLMFTLPSL
GFGEYDYLFDYGPCYVAFRRNRGYVIFCISLLLSFIIIIMVTSIWTFCFTHRFIRDHSEKGGDSVYNTQRLKLFGIFG
SMLLAYGTCYGSVIVTAFISYGIQIPEEVDAATIVAFHANPIVTALIQSYFRPDITKPLKSFKNAICSKLKKMACSFS
N

Aq1 >PAC:15724271 ID:724271 scaffold:Aqu1:Contig13491:40566:41698:1
gene:Aqu1.225743

MSLNSTDFLLTGDINAPVFSAILGIEAVIGIIANIGVLLVTLYEKKSWTKSSTMFFTSHLLANLNVSFSLLLFSIAIG
AKEWIFGITPQGKNQICIIVAYTYWNSALIIAMTLSAISFDRFLFIVKPHFHKEFMTPRVALILTITLWLLGSMINTT
PFYGIGQYGYFSSYGICTNIFQNTSMFYLTLNVSIYSAIYLTIAVMTIWTFCYTRGFIKEQATMVGSDGVYLSKEKRL
FGIFGSMLIAFIITVLPGYTIGFISAIYPLQDNVYLVNIAFNDSIIMTNPFIQLYFRPEIKSLIGSLTKKIRNKFKMK
QHSTRIRVVPINN

→ Aq2.1 Gene: Aqu2.1.38593 Scaffold Contig13491: 40,578-41,561

MSLNSTDFLLTGDINAPVFSAILGIEAVIGIIANIGVLLVTLYEKKSWTKSSTMFFTSHLLANLNVSFSLLLFSIAIG
AKEWIFGITPQGKNQICIIVAYTYWNSALIIAMTLSAISFDRFLFIVKPHFHKEFMTPRVALILTITLWLLGSMINTT
PFYGIGQYGYFSSYGICTNIFQNTSMFYLTLNVSIYSAIYLTIAVMTIWTFCYTRGFIKEQATMVGSDGVYLSKEKRL
FGIFGSMLIAFIITVLPGYTIGFISAIYPLQDNVYLVNIAFNDSIIMTNPFIQLYFRPEIKSLIGSLTKKIRNKFKMK
QHSTRIRVVPINN

Aq1 >PAC:15724373 ID:724373 scaffold:Aqu1:Contig13492:85968:87038:-1
gene:Aqu1.225845

MEMDEMTILEENKNFTLSEDINGPLLAAVIGLEMITGFLTNSFILVLTICYLKTWKQPSNIFLTNMLLNNLVIVVIVT
PFSIVTCASGEWIFGNTLSEKVAICEASAYFFTFSTTVAIESLVLISFDRFFFIVMSFEYKKYMTVNRAIIIVIISWM
LAAILISTPLYGFGNYRFSNSYGMCVPNFRNLGYAAYGSTIILVLILSIIVTSTWTYCFTRRYIKRRSSASSLSYNSN
EYQSQNRKLVGLFGMLIVIHILCYTLFVAVSALRPFYTAPRQLWATTFVLVILMTSLSPLAHAYFRYDIRSFLHRLYM
KIKGTCICSCVGTSATQKEATQSFDTSIMKTNGIHEHKTQLSIS

→ Aq2.1 Gene: Aqu2.1.38713 Scaffold Contig13492: 85,920-87,038

MEMDEMTILEENKNFTLSEDINGPLLAAVIGLEMITGFLTNSFILVLTICYLKTWKQPSNIFLTNMLLNNLVIVVIVT

PFSIVTCASGEWIFGNTLSEKVAICEASAYFFTFSTTVAIESLVLISFDRFFFIVMSFEYKKYMTVNRAIIIVIISWM
LAAILISTPLYGFGNYRFSNSYGMCVPNFRNLGYAAYGSTIILVLILSIIVTSTWTYCFTRRYIKRRSSASSLSYNSN
EYQSQNRKLVGLFGMLIVIHILCYTLFVAVSALRPFYTAPRQLWATTFVLVILMTSLSPLAHAYFRYDIRSFLHRLYM
KIKGTCICSCVGTSATQKEATQSFDTSIMKTNGIHEHKTQLSIS

Aq1 >PAC:15724923 ID:724923 scaffold:Aqu1:Contig13499:218831:219745:1
gene:Aqu1.226395

MDGVNYTLSEDINGPLLAAVLTLQMIGALVANGIVLIATLSQYKSLKLPSTILFTSLIIMHLVMALLYIPSWILSAAS
GGWIFGRTMQVKEGTCKFAGFILWYIILVIYMTLAAISVDRWLFIVKPQLYKQFMKPKVALTIIASIWILAALINSTP
FYGIGRFRYAIYGSCVPTFEEQFVYVVLMMAIFLAISAVILVTSVWTYIFTKKFIQEHSELEESSVYVSRKRRLIGIF
GVMLIAYFVCFSPGYIFGFLSQIYDLHDLSYAIALVFFMMITIINPIIQSYFRPDVKKAFVKLKIKCLNI

→ Aq2.1 Gene: Aqu2.1.39643 Contig13499: 218,364-219,804

MDGVNYTLSEDINGPLLAAVLTLQMIGALVANGIVLIATLSQYKSLKLPSTILFTSLIIMHLVMALLYIPSWILSAAS
GGWIFGRTMQVKEGTCKFAGFILWYIILVIYMTLAAISVDRWLFIVKPQLYKQFMKPKVALTIIASIWILAALINSTP
FYGIGRFRYAIYGSCVPTFEEQFVYVVLMMAIFLAISAVILVTSVWTYIFTKKFIQEHSELEESSVYVSRKRRLIGIF
GVMLIAYFVCFSPGYIFGFLSQIYDLHDLSYAIALVFFMMITIINPIIQSYFRPDVKKAFVKLKIKCLNI

Aq1

>PAC:15724924 ID:724924 scaffold:Aqu1:Contig13499:220902:221882:-1
gene:Aqu1.226396

MEKSNFTLSEDINGPLLAAALAIQMIGALVANGIVLIATLSQYKSLKLPSTMFFTSLIIIHLLMALLFIPFYMISVAA
GEWIFGRTVEEKEGTCKYVAYIFWYIILVIYMTLAAISVDRWLFIVKSQLYKQFMKPKVALTIIVSIWILAALINSTP
FYGLGEFRYSAYGCCVPVWEGQFGYLLFIASFNFIVLCVIIATSVWTFIFTRKFIQEQAALADSCVYVSRNRRLIGIF
GAMLSGYAVCFAPGFIVAVLSQIFDLELSAYATVLSLFINVIIINPMIQSYFRPEIKKVLTAFYAKCKRFCIRSSVHN
NTEAGKSFTVTSYK

→ Aq2.1 Gene: Aqu2.1.39644 Scaffold Contig13499: 220,902-222,049

MEKSNFTLSEDINGPLLAAALAIQMIGALVANGIVLIATLSQYKSLKLPSTMFFTSLIIIHLLMALLFIPFYMISVAA
GEWIFGRTVEEKEGTCKYVAYIFWYIILVIYMTLAAISVDRWLFIVKSQLYKQFMKPKVALTIIVSIWILAALINSTP
FYGLGEFRYSAYGCCVPVWEGQFGYLLFIASFNFIVLCVIIATSVWTFIFTRKFIQEQAALADSCVYVSRNRRLIGIF
GAMLSGYAVCFAPGFIVAVLSQIFDLELSAYATVLSLFINVIIINPMIQSYFRPEIKKVLTAFYAKCKRFCIRSSVHN
NTEAGKSFTVTSYK

Aq1 >PAC:15725490 ID:725490 scaffold:Aqu1:Contig13504:239296:240444:-1
gene:Aqu1.226962

MDLDSSFLDYANCSDQNDTTPLFINLTVVSNVVIAFHATFISLVIVTGLLANSTVLVLVAKDKRLRHRSIVVGLNIVF
VDILLTIFYHGVILTNCLSKGWSYNEQPEPDLICRAYGVLTTVLLNIRWFGIAVLTTDRFLTVKFPFRYEKYSRRFLI
VASLLTWTIPPGLASLLSLSLVSYSFRANIPTCLPSCINATYRMACALINTGIVMIMFIIGCIIPSGMYIWMYRKARK
MRTKYTLGELALNVTTSVAVKNIRKAQNTRSQYDRQAMVTVFLIFLSILFTSSPSFLFLIVRQISICIFFKIPVVIHF
IVTDIFIVSTALDPLVLMKNHDFRTVIYELFHKKCWCRHHVTDGRTSSTEAETQSSSANINDMHETNLKN

→ Aq2.1 Gene: Aqu2.1.40381 Scaffold Contig13504: 239,196-240,467

MDLDSSFLDYANCSDQNDTTPLFINLTVVSNVVIAFHATFISLVIVTGLLANSTVLVLVAKDKRLRHRSIVVGLNIVF
VDILLTIFYHGVILTNCLSKGWSYNEQPEPDLICRAYGVLTTVLLNIRWFGIAVLTTDRFLTVKFPFRYEKYSRRFLI
VASLLTWTIPPGLASLLSLSLVSYSFRANIPTCLPSCINATYRMACALINTGIVMIMFIIGCIIPSGMYIWMYRKARK
MRTKYTLGELALNVTTSVAVKNIRKAQNTRSQYDRQAMVTVFLIFLSILFTSSPSFLFLIVRQISICIFFKIPVVIHF
IVTDIFIVSTALDPLVLMKNHDFRTVIYELFHKKCWCRHHVTDGRTSSTEAETQSSSANINDMHETNLKN

Aq1 >PAC:15726227 ID:726227 scaffold:Aqu1:Contig13509:476758:479483:-1
gene:Aqu1.227699

MEEEYIENDNFTLSEEINGPLLAAVIAVEMVAGLIANIFVLALSCFHCEIYKKPSTVFLTNMLVANLIMIVVVMPVPI

ATCVSGEWIFGSTVSSKLASCEAMGTLFAWSTLIATESLVLLSFDRFFFIVKAGKYNEHMTVKKALIIVVASWILATI
LVSPPQYGFGGFNFAESYGLCGPSFRSVGFSVLIIPLPVTFMLHLMLNLPQVL

→ Aq2.1 Gene: Aqu2.1.41279 Scaffold Contig13509: 476,049-477,390

ALIPVPPISVDDDDEENPQIIQNENDSRSSNSREPLVFTQPQFTHDFHLIYCRNQSPSDNFLMLAIIQLFLCSLIGVL
ALFCIIPALYFAVKARNAEMNGDIIRMYSKRQLALIFNFLGFFVGFINIISHYPATSNIYVTPHAKLTSSFNDSRSSN
NREIVFTRPQFAHDFHLVYQRNQSQ

AND
→ Aq2.1 Gene: Aqu2.1.41280 Scaffold Contig13509: 478,873-479,483

MEEEYIENDNFTLSEEINGPLLAAVIAVEMVAGLIANIFVLALSCFHCEIYKKPSTVFLTNMLVANLIMIVVVMPVPI
ATCVSGEWIFGSTVSSKLASCEAMGTLFAWSTLIATESLVLLSFDRFFFIVKAGKYNEHMTVKKALIIVVASWILATI
LVSPPQYGFGGFNFAESYGLCGPSFRSVGFSVYGSVIIGSLIISIVV

Aq1 >PAC:15726228 ID:726228 scaffold:Aqu1:Contig13509:480547:481672:-1
gene:Aqu1.227700

MEEEYIENNNFTLSEDINGPLLAAVIGVEMVAGLIANIFVLTLSCFHCETYKKPSTIFLTNMLVANLIIIVVVMPFSI
ATCVSGEWIFGSTVSSKLASCEAMGTLFGWSTLIATESLVLLSFDRFFFIVKAGKYNEHMTVKKALIIVAASWILAAI
LVSPPQYGFGGFVFANSYGLCGPNFRSVGFSVYGFFIIGSLIISIVVTSLWTYCYTKKFIKEMNSRMLRESVYFSQHT
KLVGIFGTLIIVHIFCYSLFLSVSVVRPFVAIPRQLWATTLVFLLLITILSPLVQSYFRSETRNFIQNLLLKIRLSKS
HPTLPTRMTVTSLIKLKAPSQTLSE

→ Aq2.1 Gene: Aqu2.1.41281 Scaffold Contig13509: 480,514-481,768

MWSSCLQTEACIISQAKATQLTVNLTPSTLYRMEEEYIENNNFTLSEDINGPLLAAVIGVEMVAGLIANIFVLTLSCF
HCETYKKPSTIFLTNMLVANLIIIVVVMPFSIATCVSGEWIFGSTVSSKLASCEAMGTLFGWSTLIATESLVLLSFDR
FFFIVKAGKYNEHMTVKKALIIVAASWILAAILVSPPQYGFGGFVFANSYGLCGPNFRSVGFSVYGFFIIGSLIISIV
VTSLWTYCYTKKFIKEMNSRMLRESVYFSQHTKLVGIFGTLIIVHIFCYSLFLSVSVVRPFVAIPRQLWATTLVFLLL
ITILSPLVQSYFRSETRNFIQNLLLKIRLSKSHPTLPTRMTVTSLIKLKAPSQTLSE

Aq1 >PAC:15727588 ID:727588 scaffold:Aqu1:Contig13518:220905:222439:1
gene:Aqu1.229060

MEDNFTFIGEVGGPAVATILIIEMILALIANGVVLSITLYQRKSWKQPSTIFFTSLILAHFVLNLLYLPFIIVTFVAR
EWVVGSTDEEKKWTCYYITFIFWWMVLVIFMTLAAVSFDRFLFIVKPHLHKRFMRPWVALTLTIAIWIFSAIYSIVQI
FGFHLYIAICLTPAAGIGVFFFLLSVTSIILVIIFVTSLWTFCFTKSFFKDQSLIAGESVYASKKKRLFGIFGSMLLI
YGICFVPVIVLPLWFDIFATICFLFITVANPVIQSYFRPEIKIS

→ Aq2.1 Gene: Aqu2.1.43059 Scaffold Contig13518: 220,905-221,807

MEDNFTFIGEVGGPAVATILIIEMILALIANGVVLSITLYQRKSWKQPSTIFFTSLILAHFVLNLLYLPFIIVTFVAR
EWVVGSTDEEKKWTCYYITFIFWWMVLVIFMTLAAVSFDRFLFIVKPHLHKRFMRPWVALTLTIAIWIFSAIYSIVQI
FGFHLYIAICLTPAAGIGVFFFLLSVTSIILVIIFVTSLWTFCFTKSFFKDQSLIAGESVYASKKKRLFGIFGSMLLI
YGICFVPGVIYFFLSLVIVLPLWFDIFATICFLFITVANPVIQSYFRPEIKSVLVSYFPFLFCVCC

Aq1 >PAC:15727589 ID:727589 scaffold:Aqu1:Contig13518:223413:224838:1
gene:Aqu1.229061

MESNFTLTDGISGPVLATILIVQIILALIANGVVLSITFYQWKSLKLSSTIFFTSVILAHLVLNLLYLPFRIIALAAG
EWVFGSTNEEKKVTCTFAAFFFWWMVLVILMTVAAISFDRFLFIVKPQLHKRFMRSWVALTLTIAIWILSAALSNVHI
FAFHLYIAICIRPSNSIGLITILGIIFVTSLWTFCFTRSFFKNQSVIAAGSVYASKKKRLFGVFGSMLLIYGICFVPG
FLYFFLSLFIDVPVWFETFAIICFDFITVANPIIQSYFRPEIKTLALIAYGAVVTIYPTLSKEILFKQSLLYHYLLPL
HLLE

→ Aq2.1 Gene: Aqu2.1.43060 Scaffold Contig13518: 223,413-224,315

MESNFTLTDGISGPVLATILIVQIILALIANGVVLSITFYQWKSLKLSSTIFFTSVILAHLVLNLLYLPFRIIALAAG
EWVFGSTNEEKKVTCTFAAFFFWWMVLVILMTVAAISFDRFLFIVKPQLHKRFMRSWVALTLTIAIWILSAALSNVHI
FAFHLYIAICIRPSNSIGLSIYFVLLSVTILGIIFVTSLWTFCFTRSFFKNQSVIAAGSVYASKKKRLFGVFGSMLLI
YGICFVPGFLYFFLSLFIDVPVWFETFAIICFDFITVANPIIQSYFRPEIKSVLVPYFPFHICVCC

Aq1 >PAC:15728243 ID:728243 scaffold:Aqu1:Contig13521:18775:19746:-1
gene:Aqu1.229715

MSFNSTDFVLTGDINSPTYAAVLGIEGVIGIIANVAVLLMTLYQRKSWNQPSTIFFTSLLLPNLIIALCYLMSSIAVG
AEEWIFGNTFEQKNGSCMFVGYILWNGIMIIAATLAAISFDRFLFIVKPHLHKQFMRPRVALILIIGVWLLSSLINTI
PFYSTGGYGYFPPNGICSFLNETSPYYFVVLILVYAISYSIMAFTSIWTFFFTRSFIKRQGRSVDSGVYQSKNKKLFG
IFGSMLLSYILSFFPSYIVGFFVYFGLPDPDGLYAFTFCTYGLVVIINPLIQSYFRPEIKEVLASFANKIGLKSNKIL
PVRIQVEPKPD

→ Aq2.1 Gene: Aqu2.1.43851 Scaffold Contig13521: 18,775-19,746

MSFNSTDFVLTGDINSPTYAAVLGIEGVIGIIANVAVLLMTLYQRKSWNQPSTIFFTSLLLPNLIIALCYLMSSIAVG
AEEWIFGNTFEQKNGSCMFVGYILWNGIMIIAATLAAISFDRFLFIVKPHLHKQFMRPRVALILIIGVWLLSSLINTI
PFYSTGGYGYFPPNGICSFLNETSPYYFVVLILVYAISYSIMAFTSIWTFFFTRSFIKRQGRSVDSGVYQSKNKKLFG
IFGSMLLSYILSFFPSYIVGFFVYFGLPDPDGLYAFTFCTYGLVVIINPLIQSYFRPEIKEVLASFANKIGLKSNKIL
PVRIQVEPKPD

Aq1 >PAC:15728244 ID:728244 scaffold:Aqu1:Contig13521:24043:25020:-1
gene:Aqu1.229716

MDSMSFNSTDFVLTGDINSPTYAAALGIEGVIGIIANVAVLLMTLYQRKSWNQPSTIFFTSLLLSSLIIALVYLMSSF
AVGAEEWIFGNTFEEKNGSCIFFGFFLYYGGMLNAATLAVISFDRFLFIVKPHLHKQFMRPRVALILIIGVWLLCSLI
NTIPFYGFGVFGYLYDNGVCSLIYEHSGLYLLVLLVVYPMIYCIIASTSIWTFFFTRSFIKRQGRSVDSGVYQSKNKK
LFGIFGSMLLSYILTLLPSYFVALLVFIFGQLDSAFAFGIGTYGLISIMNPLIQSYFRPEIKETLVLFAKKIGLKSNK
IHPIEPQVAVTAD

→ Aq2.1 Gene: Aqu2.1.43854 Scaffold Contig13521: 23,886-25,128

MMDSMSFNSTDFVLTGDINSPTYAAALGIEGVIGIIANVAVLLMTLYQRKSWNQPSTIFFTSLLLSSLIIALVYLMSS
FAVGAEEWIFGNTFEEKNGSCIFFGFFLYYGGMLNAATLAVISFDRFLFIVKPHLHKQFMRPRVALILIIGVWLLCSL
INTIPFYGFGVFGYLYDNGVCSLIYEHSGLYLLVLLVVYPMIYCIIASTSIWTFFFTRSFIKRQGRSVDSGVYQSKNK
KLFGIFGSMLLSYILTLLPSYFVALLVFIFGQLDSAFAFGIGTYGLISIMNPLIQSYFRPEIKETLVLFAKKIGLKSN
KIHPIEPQVAVTAD
