# Supplementary material for: Potential for host-symbiont communication via neurotransmitters and neuromodulators in an aneural animal, the marine sponge Amphimedon queenslandica
Source: Front Neural Circuits. 2023 Sep 29;17:1250694. doi: 10.3389/fncir.2023.1250694 (PMC10570526; doi:10.3389/fncir.2023.1250694)
Supplement: Supplementary file 6 [file Image_2.pdf]

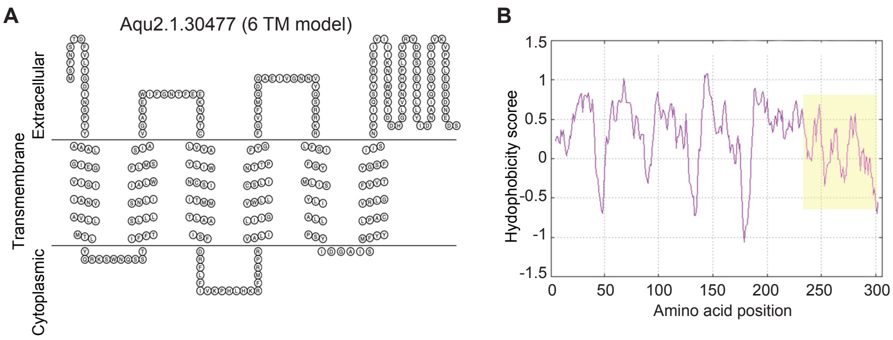

C

```
DRD1_Sponge      -MSFNS-----TDFVLTGDINS----PTYAAALGIEGVIGIIANVAVLLMTLYQRKSWNQ   50
DRD1_Human       MRTLNTSAMDGTGLVVERDFSVRILTACFLSLLILSTLLG---NTLVCAAVIRFRHLRSK   57
DRD1_Rat         -MAPNTSTMDEAGLPAERDFSFRILTACFLSLLILSTLLG---NTLVCAAVIRFRHLRSK   56
                   : *:      :.:    *:.        : : * :. ::*    *. *    .:   *:   .:

DRD1_Sponge      SSTIFFTSLLLSNLIIALWFL-MSSIAVGAEEWIFGNTFEEKNATCLVVAYLIWNCSIIT   109
DRD1_Human       VTNFFVISLAVSDLLVAVLVMPWKAVAEIAGFWPFGS-------FCNIWVAFDIMCSTAS   110
DRD1_Rat         VTNFFVISLAVSDLLVAVLVMPWKAVAEIAGFWPLGP-------FCNIWVAFDIMCSTAS   109
                  :.:*. ** :*:*::*: .:   .::*  *  * :*          * : . :    **  :

DRD1_Sponge      MMTLAAISFDRFLFIVKPHLHKRFMRPRVALILIIGVWLLCSLINTTPFYGFGVFMQDQA   169
DRD1_Human       ILNLCVISVDRYWAISSPFRYERKMTPKAAFILISVAWTLSVLISFIPVQLSWHKAKPTS   170
DRD1_Rat         ILNLCVISVDRYWAISSPFQYERKMTPKAAFILISVAWTLSVLISFIPVQLSWHKAKPTW   169
                 ::.*..**.**:  * .*. ::* * *:.*:***  .* *. **.  *.        :

DRD1_Sponge      EIVGN---------NVYQSRRKRLFGIFGYMLISYLIALLPSYIDGAISMFYYLPACVRL   220
DRD1_Human       PSDGNATSLAET-IDNCDSSLSRTYAISSSV-----------------ISFYIPVAIM-   210
DRD1_Rat         PLDGNFTSLEDTEDDNCDTRLSRTYAISSSL-----------------ISFYIPVAIM-   210
                   **          :  ::  .* :.* . :                  :  :*:*..:

DRD1_Sponge      GFVVTYGSF-IISNPIIQSYFRPEI-VIIIKNLWNKIDDHGLVNFHPLDVRVDE------   272
DRD1_Human       --IVTYTRIYRIAQKQIRRIAALERAAVHAKNCQ-----TTTGNGKPVECSQPESSFKMS   263
DRD1_Rat         --IVTYTSIYRIAQKQIRRISALERAAVHAKNCQ-----TTAGNGNPVECAQSESSFKMS   263
                   :***  :  *::  *:       *   .:  **           * :*::     *

DRD1_Sponge      ------------------------------------------------------------   272
DRD1_Human       FKRETKVLKTLSVIMGVFVCCWLPFFILNCILPFCGSGETQPFCIDSNTFDVFVWFGWAN   323
DRD1_Rat         FKRETKVLKTLSVIMGVFVCCWLPFFISNCMVPFCGSEETQPFCIDSITFDVFVWFGWAN   323

DRD1_Sponge      ------------------------------SLETLL------------------------   278
DRD1_Human       SSLNPIIYAFNADFRKAFSTLLGCYRLCPATNNAIETVSINNNGAAMFSSHHEPRGSISK   383
DRD1_Rat         SSLNPIIYAFNADFQKAFSTLLGCYRLCPTTNNAIETVSINNNGAVVFSSHHEPRGSISK   383
                                               ::**:

DRD1_Sponge      ----L-YIDNAIQYGEDIDV--------------------------KVPKLEDDDN--E   304
DRD1_Human       ECNLVYLIPHAVGSSEDLKKEEAAGIARPLEKLSPALSVILDYDTDVSLEKIQPITQNGQ   443
DRD1_Rat         DCNLVYLIPHAVGSSEDLKKEEAGGIAKPLEKLSPALSVILDYDTDVSLEKIQPVTHSGQ   443
                     :  * :*:  .**:.                          .: *::   :  :

DRD1_Sponge      DS-     306
DRD1_Human       HPT     446
DRD1_Rat         HST     446
```

D

```
DRD1_Sponge      ------------------------------------------------------------   0
DOPR1_DROME      MYTPHPFGFLIILVPMTNAMRAIAAIAAGVGSVAATVATSTTSSISSSTTIINTSSATTI   60

DRD1_Sponge      -----------------------------------------------------MSFNSTD   7
DOPR1_DROME      GGNHTSGSTGFSTNSTLLDADHLPLQLTTAKVDLDIEIDIQLLTNGYDGTTLTSFYNESS   120
                                                                      :*.:.

DRD1_Sponge      FV-------LTGDINSPTYAAALGIEGVIGIIA---------NVAVLLMTLYQRKSWNQS   51
DOPR1_DROME      WTNASEMDTIVGEEPEPLSLVSI---VVVGIFLSVLIFLSVAGNILVCLAIYTERSLRRI   177
                 :.        :.*:  .*   .::     *:**:         .  :: ::* .:* .:

DRD1_Sponge      STIFFTSLLLSNLIIALW-FLMSSIAVGAEEWIFGNTFEEKNATCLVVAYLIWNCSIITM   110
DOPR1_DROME      GNLFLASLAIADLFVASLVMTFAGVNDLLGYWIFGAQFCDTWV------AFDVMCSTASI   231
                 ..:*::** ::*::*   : ::.:      ****  * :. .         :   **  ::

DRD1_Sponge      MTLAAISFDRFLFIVKPHLHKRFMRPRVALILIIGVWLLCSLINTTPFYGFGVFMQDQAE   170
DOPR1_DROME      LNLCAISMDRYIHIKDPLRYGRWVTRRVAVITIAAIWLLAAFVSFVPI-SLGIHRPDQPL   290
                 :.*.***:**::.* .*  : *::  ***:* * .:***.:::. .*: .:*:.  **

DRD1_Sponge      IVGNNVYQSRRKRLFGIFGYMLISYLIALLPSYIDGAISMFYYLPACVRLGFVVTYGSFI   230
DOPR1_DROME      IFEDN-------------GKKYPTCALDLTPTYAVVSSCISFYFPCVVMIGIYCRLYCYA   337
                 *. :*             *    :  : * *:*   : .: :*:*. * :*:       .:

DRD1_Sponge      ISN-PIIQSYFRPEIVIIIKNLWNKID-------DHGLVNFHP----LDV-------RVD   271
DOPR1_DROME      QKHVKSIKAVTRPGEVAE-KQRYKSIRRPKNQPKKFKVRNLHTHSSPYHVSDHKAAVTVG   396
                  .:   *::  **  *   *: ::.*          .. : *:*       .*        *.

DRD1_Sponge      ESLETLLL-----------------------------Y-------------------   280
DOPR1_DROME      VIMGVFLICWVPFFCVNITAAFCKTCIGGQTFKILTWLGYSNSAFNPIIYSIFNKEFRDA   456
                   : .:*:                                 *

DRD1_Sponge      ------IDNAIQYGEDIDVKVPKLEDDDNEDS----------------------   306
DOPR1_DROME      FKRILTMRNPWCCAQDVGNIHPRNSDRFITDYAAKNVVVMNSGRSSAELEQVSAI   511
                       : *    .:*:.   *: .*    *
```

Supplementary figure 2. Evidence for AquDRD1-like being a dopamine-like receptor. (A-C) Determination of a 7th transmembrane region in AquDRD1-like. (A) Snake plot of Aqu2.1.30477 yields only 6 TM domains, 3 intracellular and 2 extracellular loops. (B) Hydrophobicity plot of Aqu2.1.30477 (Eisenberg et al., 1984) identifies a putative determination of the 7th TM region in AquDRD1-like; putative 7th TM region highlighted in yellow. (C) Sequence alignment of A. queenslandica putative DRD1-like (Aqu2.1.30477), with human [sp|P21728|DRD1_HUMAN D(1A)] and rat [sp|P18901|DRD1_RAT D(1A)] D1-like, D1 receptor sequences. Highlighted in yellow is the most conserved region. (D) Sequence alignment of A. queenslandica putative DRD1-like (Aqu2.1.30477), with the Drosophila (sp|P41596|DOPR1_DROME Dopamine receptor 1) Dop1R1 sequence. Highlighted in yellow is the most conserved region.
