# Supplementary material for: Potential for host-symbiont communication via neurotransmitters and neuromodulators in an aneural animal, the marine sponge Amphimedon queenslandica
Source: Front Neural Circuits. 2023 Sep 29;17:1250694. doi: 10.3389/fncir.2023.1250694 (PMC10570526; doi:10.3389/fncir.2023.1250694)
Supplement: Supplementary file 7 [file Image_3.pdf]

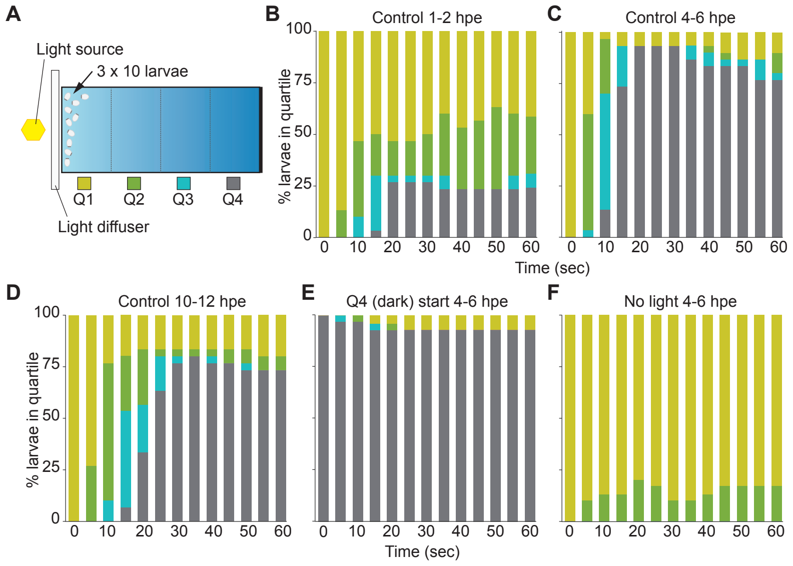

Supplementary figure 3. Normal larval phototactic swimming behaviour. (A) Schematic of larval phototaxis assay chamber. Triplicate 1 min assays with 10 larvae loaded into Q1 (bright) were performed [see Materials and methods, and Wong et al. (2022) for details]. (B-F) Distribution of larvae across the chamber (Q1-Q4) at 5 sec intervals. Colour scheme follows legend in A. (B) 1-2 hpe larvae. (C) 4-6 hpe larvae. (D) 10-12 hpe larvae. (E) 4-6 hpe larvae placed in Q4 (dark end of the assay chamber) at start of experiment, showing that do not swim toward the light (i.e. exhibit positive phototaxis). (F) 4-6 hpe larvae placed in Q1 without a light gradient, showing that the process of adding larvae to the assay chamber does not cause them to swim to the other end of the chamber (see Materials and methods).
