# Supplementary material for: Potential for host-symbiont communication via neurotransmitters and neuromodulators in an aneural animal, the marine sponge Amphimedon queenslandica
Source: Front Neural Circuits. 2023 Sep 29;17:1250694. doi: 10.3389/fncir.2023.1250694 (PMC10570526; doi:10.3389/fncir.2023.1250694)
Supplement: Supplementary file 8 [file Image_4.pdf]

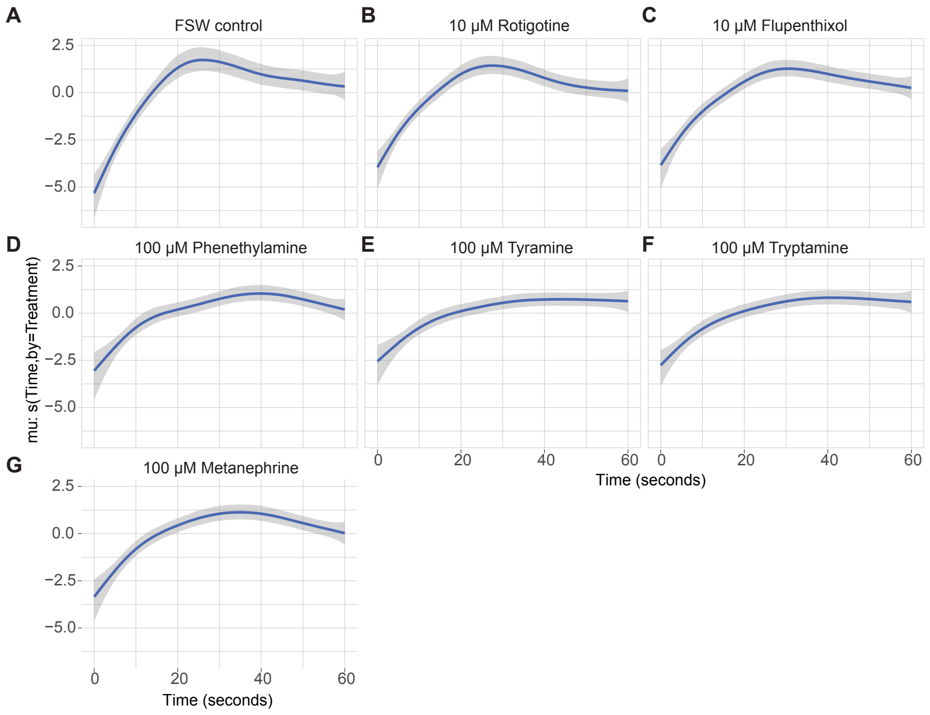

Supplementary figure 4. Mean swimming velocity of control and treated larvae at each time for one minute. Shaded areas are 95% credibility regions. Time courses for the drug treatments. (A) FSW control. (B) DRD agonist 10 μM rotigotine hydrochloride. (C) DRD antagonist 10 μM flupenthixol dihydrochloride. Trace amines and TAAR agonists: (D) 100 μM phenethylamine; (E) tyramine; and (F) tryptamine. (G) Non-specific TAAR agonist 100 μM D,L-metanephrine hydrochloride.
