# Supplementary material for: Potential for host-symbiont communication via neurotransmitters and neuromodulators in an aneural animal, the marine sponge Amphimedon queenslandica
Source: Front Neural Circuits. 2023 Sep 29;17:1250694. doi: 10.3389/fncir.2023.1250694 (PMC10570526; doi:10.3389/fncir.2023.1250694)
Supplement: Supplementary file 9 [file Image_5.pdf]

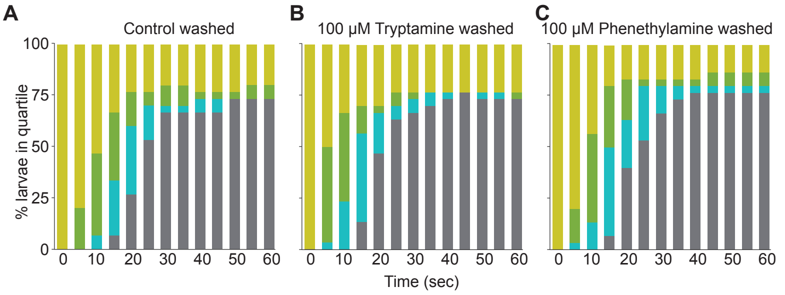

Supplementary figure 5. Normal negative phototaxis in larvae initially treated with tryptamine and phenethylamine and then washed and re-assayed in FSW. Trace amines and TAAR agonists tryptamine and phenethylamine markedly inhibited larval negative phototaxis compared to FSW controls (Figure 4). After the completion of these and control (FSW) phototaxis assays, larvae were washed in FSW (see Materials and methods) and larvae were re-assayed in FSW. FSW controls (A), tryptamine (B) and phenethylamine (C) phototaxis assays are similar, with larvae previously assayed in tryptamine and phenethylamine being mildly different from FSW controls, indicating that the effect of these trace amines is reversible.
