# Supplementary material for: Potential for host-symbiont communication via neurotransmitters and neuromodulators in an aneural animal, the marine sponge Amphimedon queenslandica
Source: Front Neural Circuits. 2023 Sep 29;17:1250694. doi: 10.3389/fncir.2023.1250694 (PMC10570526; doi:10.3389/fncir.2023.1250694)
Supplement: Supplementary file 5 [file Image_1.pdf]

```
AqS1v2_02414            GAFVTGATMASFTALAAARRRVLLAHG-HDVDRDGLAKAPPITVAVGESAHATVFKALGL   204
sp|P16453|DCHS_RAT      GVLQRTVSESTLIALLAARKNKILEMKAHEPNADESSLNARLVAYASDQAHSSVEKAGLI   206
sp|P19113|DCHS_HUMAN    GVLQSTVSESTLIALLAARKNKILEMKTSEPDADESCLNARLVAYASDQAHSSVEKAGLI   203
sp|Q5EA83|DCHS_BOVIN    GVLQSTVSESTLIALLAARKNKILEMKASEPEADESFLNARLVAYASDQAHSSVEKAGLI   203
sp|Q05733|DCHS_DROME    GVLQTTASEATLVCLLAGRTRAIQRFHERHPGYQDAEINARLVAYCSDQAHSSVEKAALI   202
sp|P05031|DDC_DROME     GVIQGTASESTLVALLGAKAKKLKEVKELHPEWDEHTILGKLVGYCSDQAHSSVERAGLL   236
sp|P14173|DDC_RAT       GVIQGSASEATLVALLAARTKMIRQLQAASPELTQAALMEKLVAYTSDQAHSSVERAGLI   201
sp|P27718|DDC_BOVIN     GVIQGTASEATLVALLAARTKVTRHLQAASPELMQAAIMEKLVAYASDQAHSSVEKAGLI   201
sp|P20711|DDC_HUMAN     GVIQGSASEATLVALLAARTKVIHRLQAASPELTQAAIMEKLVAYSSDQAHSSVERAGLI   201
AqS1v2_02410            GVIQDTASSATLAAVLTMRERALDGKGNSR----GLAGQPALRIYASNEVHSSIDRALWF   189
AqS2v2_00368            GVIQDSASSGTLAAVLTARERATGWQGNEA----GLAGGPPLRMYYSRHAHASVPKAIML   189
                        *.:   .: .:: .:   : .                    :    .  .*::: :*  :
                              ▲▲                                          ▲

AqS1v2_02414            LGLGRAQVVKVEADS-QGRMRPDALSRL--------QGPAIVCAQAGNVNTGAIDPIRRI   255
sp|P16453|DCHS_RAT      SLVK---IKFLPVDD-NFSLRGEALQKAIEEDKQQGLVPVFVCATLGTTGVCAFDKLSEL   262
sp|P19113|DCHS_HUMAN    SLVK---MKFLPVDD-NFSLRGEALQKAIEEDKQRGLVPVFVCATLGTTGVCAFDCLSEL   259
sp|Q5EA83|DCHS_BOVIN    SLVK---MKFLPVDE-NFSLRGEALQKAIKEDRERGLVPIFVCATLGTTGVCAFDCLSEL   259
sp|Q05733|DCHS_DROME    GLVR---MRYIEADD-DLAMRGKLLREAIEDDIKQGLVPFWVCATLGTTGSCSFDNLEEI   258
sp|P05031|DDC_DROME     GGVK---LRSVQSE--NHRMRGAALEKAIEQDVAEGLIPFYAVVTLGTTNSCAFDYLDEC   291
sp|P14173|DDC_RAT       GGVK---IKAIPSDG-NYSMRAAALREALERDKAAGLIPFFVVVTLGTTSCCSFDNLLEV   257
sp|P27718|DDC_BOVIN     GGVR---LKAIPSDG-KFAMRASALQEALERDKAAGLIPFFVVATLGTTSCCSFDNLLEV   257
sp|P20711|DDC_HUMAN     GGVK---LKAIPSDG-NFAMRASALQEALERDKAAGLIPFFMVATLGTTTCCSFDNLLEV   257
AqS1v2_02410            SGIGADNLVRIPTAGPMRGMILERLRDAIAADRAAGFLPAGIVAAVGGTSTGACDDIAAV   249
AqS2v2_00368            AGLGRANAVAIDLDA-DGAMDAGALERAIEADRAAGMKPAGVVATVGATSTGDADGLAAT   248
                          :       :        :    *             *    .  * .     * :
                                                                ▲

AqS1v2_02414            CAHVPAADIWVHVDGAFGLWVRALRSADALDIDPARKAKASMIADQGAGIEDADSWATDA   315
sp|P16453|DCHS_RAT      GPICAREGLWLHVDAAYAGTAF---------LRP-------ELRGFLKGIEYADSFTFNP   306
sp|P19113|DCHS_HUMAN    GPICAREGLWLHIDAAYAGTAF---------LCP-------EFRGFLKGIEYADSFTFNP   303
sp|Q5EA83|DCHS_BOVIN    GPICAREGLWLHIDAAYAGTAF---------LCP-------EFRGFLKGIEYADSFTFNP   303
sp|Q05733|DCHS_DROME    GIVCAEHHLWLHVDAAYAGSAF---------ICP-------EFRTWLRGIERADSIAFNP   302
sp|P05031|DDC_DROME     GPVGNKHNLWIHVDAAYAGSAF---------ICP-------EYRHLMKGIESADSFNFNP   335
sp|P14173|DDC_RAT       GPICNQEGVWLHIDAAYAGSAF---------ICP-------EFRYLLNGVEFADSFNFNP   301
sp|P27718|DDC_BOVIN     GPICHEEGLWLHVDAAYAGSAF---------ICP-------EFRHLLNGVEFADSFNFNP   301
sp|P20711|DDC_HUMAN     GPICNKEDIWLHVDAAYAGSAF---------ICP-------EFRHLLNGVEFADSFNFNP   301
AqS1v2_02410            SQVAQEESLYLHVDAAWAGSAM---------ICP-------EFRSLWHGAEHADSIVLNA   293
AqS2v2_00368            GAVVRRHGLYGHVDAAWAGSAA---------LCP-------EHRGLLDGLEQWDSYLFNP   292
                                :: *:*.*:.  .          : *              * *  **   :
                                                                          ▲

AqS1v2_02414            HKWLNVPYDCGIALVRDAEALRGAMSVQADYLPQASRG----EP--F-EHTPEASRRMRS   368
sp|P16453|DCHS_RAT      SKWMMVHFDCTGFWVKDKYKLQQTFSVNPIYLRHA--N--SGVATDFMHWQIPLSRRFRS   362
sp|P19113|DCHS_HUMAN    SKWMMVHFDCTGFWVKDKYKLQQTFSVNPIYLRHA--N--SGVATDFMHWQIPLSRRFRS   359
sp|Q5EA83|DCHS_BOVIN    SKWMMVHFDCTGFWVKDKYKLQQTFSVDPVYLRHA--D--SGVATDFMHWQIPLSRRFRS   359
sp|Q05733|DCHS_DROME    SKWLMVHFDATALWVRDSTAVHRTFNVEPLYLQHE--N--SGVAVDFMHWQIPLSRRFRA   358
sp|P05031|DDC_DROME     HKWMLVNFDCSAMWLKDPSWVVNAFNVDPLYLKHDMQ----GSAPDYRHWQIPLGRRFRA   391
sp|P14173|DDC_RAT       HKWLLVNFDCSAMWVKKRTDLTEAFNMDPVYLRHSHQD--SGLITDYRHWQIPLGRRFRS   359
sp|P27718|DDC_BOVIN     HKWLLVNFDCSAMWVKKRTDLTGAFRLDPVYLRHSHQD--SGLITDYRHWQLPLGRRFRS   359
sp|P20711|DDC_HUMAN     HKWLLVNFDCSAMWVKKRTDLTGAFRLDPTYLKHSHQD--SGLITDYRHWQIPLGRRFRS   359
AqS1v2_02410            HKWLGAQLECSIHLLKDENALRNTLTITREYLGTHGQQSARGELRNLSDLSLQLGRRFRA   353
AqS2v2_00368            HKWLGTNFDLCAHYLKDPAAQIRTFGAQPDYLQTAGVEQ----PADFSSWTAPLGRRFRA   348
                         **: .  :     ::.      ::     **                      .**:*:
                         ▲                                                    ▲
```

Supplementary figure 1. Partial alignment of aromatic amino acid decarboxylase and histidine decarboxylase. Aqs1v2_02410 and aqs2v2_00368 are putative aromatic amino acid decarboxylases in bacterial symbionts AqS1 and AqS2. Aqs1v2_02414 is a putative histidine decarboxylase in AqS1. Rattus norvegicus (sp|P14173|DDC_RAT, sp|P16453|DCHS_RAT), Homo sapiens (sp|P20711|DDC_HUMAN, sp|P19113|DCHS_HUMAN), Bos taurus (sp|P27718|DDC_BOVIN, sp|Q5EA83|DCHS_BOVIN) and Drosophila melanogaster (sp|P05031|DDC_DROME, sp|Q05733|DCHS_DROME) aromatic amino acid decarboxylase and histidine decarboxylase are aligned to the symbiont sequences. The colour triangles indicate catalytic residues: red, conserved PLP binding sites; blue, decarboxylation residue; green, substrate specificity; and black, PLP binding sites.
